# Efficacy and safety of different antimicrobial DURATions for the treatment of Infections associated with Osteosynthesis Material implanted after long bone fractures (DURATIOM): Protocol for a randomized, pragmatic trial

**DOI:** 10.1371/journal.pone.0286094

**Published:** 2023-05-22

**Authors:** Carmen Garrigós, Clara María Rosso-Fernández, Irene Borreguero, Patricia Rodríguez, Raquel García-Albea, Jose María Bravo-Ferrer, Jesús Rodríguez-Baño, María Dolores del Toro, on behalf of the DURATIOM team

**Affiliations:** 1 Infectious Diseases and Microbiology Clinical Unit, University Hospital Virgen Macarena, Sevilla, Spain; 2 Department of Medicine, School of Medicine, University of Sevilla, Seville, Spain; 3 Biomedicine Institute of Sevilla (IBiS)/CSIC, Seville, Spain; 4 Unidad de Investigación Clínica y Ensayos Clínicos (CTU), Hospital Virgen del Rocío, Sevilla, Spain; 5 Orthopaedic and Trauma Unit, University Hospital Virgen Macarena, Seville, Spain; 6 Centro de Investigación en Red de Enfermedades Infecciosas (CIBERINFEC), Instituto de Salud Carlos III, Madrid, Spain; Universita degli Studi di Milano, ITALY

## Abstract

**Background:**

Infection associated with osteosynthesis material (IOM) is one of the most feared and challenging complications of trauma surgery and can cause significant functional loss, requiring multiple interventions and excessive consumption of antimicrobials. Evidence is needed about the best surgical procedure and the duration of antibiotic treatment according to the age of the implant or onset of infection symptoms, as it considers the biofilm formation and the state of fracture healing. There were not clinical trials evaluating the optimal duration of antibiotic therapy in IOM when implant is retained. Because there are antibiotics that have proven to be effective for the treatment of infection associated to implant, mainly in PJI, these antibiotics could be used in these infections. Investigating whether shorter duration of treatment is a priority in infectious diseases, as a way to reduce the exposure to antibiotics and help in controlling antimicrobial resistance and avoiding unnecessary adverse events and cost. We aim to describe the hypothesis, objectives, design, variables and procedures for a pragmatic randomized controlled trial comparing different durations of antibiotic treatment in IOM after long bone fractures treated with debridement and implant retention.

**Methods and design:**

This is a multicenter, open-label, non-inferiority, randomized, controlled, pragmatic phase 3 trial, comparing different durations of antibiotic treatment in IOM after long bone fractures treated with debridement and implant retention. Patients with microbiologically confirmed IOM will be included. Eligible patients are those older than 14 years, with early IOM (up to 2 weeks after the implant surgery) and delayed IOM (between 3 and 10 weeks after the implant surgery) with stabilized fracture and absence of bone exposure who sign the informed consent. Randomization will be 1:1 to receive a short-term antibiotic treatment (8 weeks in early IOM and 12 weeks in delayed IOM) or a long-term antibiotic treatment (12 weeks in early IOM or until fracture healing or implant removal in delayed IOM). The antibiotic treatment will be that used in routine practice by the specialist in infectious diseases. The primary outcome is the composited variable "cure" that includes clinical cure, radiological healing, and definitive soft tissue coverage, which will be evaluated in the test of cure at 12 months after the end of antibiotic therapy. Adverse events, resistance development during therapy and functional status will be collected. A total of 364 patients are needed to show a 10% non-inferiority margin, with 80% power and 5% one-sided significance level.

**Discussion:**

If the hypothesis of non-inferiority of short vs. long antibiotic treatments is demonstrated, and the efficacy of antibiotics with less ecological impact in long treatments, the impact on reduction of bacterial resistance, toxicity and health costs will be observed.

**Trial registration:**

This trial is registered at ClinicalTrials.gov (NCT05294796) on Jan 26^th^ 2022 and at the European Union Drug Regulating Authorities Clinical Trials (EUDRACT) (2021-003914-38) on Jul 16^th^ 2021. The Sponsor Study Code is DURATIOM.

## Introduction

Orthopedic implants for fracture fixation or osteosynthesis material are used for internal fixation of fractures, allowing their stability and consolidation. They are only temporarily needed and can be removed after healing of the bone fracture. Infections associated with osteosynthesis material (IOM) are among the most feared and challenging complications of trauma surgery and can lead to limb function loss or even amputation. A recent systematic review focusing on late onset IOM stated that recurrence of disease occurs in 6–9% patients, leading to amputation of the affected limb in 3–5% [1]. The risk of infection after internal fixation is between 1–30% [2]. This wide range is due to variation in host related factors, fracture type and location, open vs. closed fracture, type of fixation, delayed debridement in open fractures, and perioperative factors (antibiotic prophylaxis, NNIS index, asepsis, surgical technique, perioperative wound care) [3]. A particularly vulnerable population is the elderly, in whom fractures of the lower limbs due to falls are frequent. A study performed in patients with staphylococcal orthopaedic device-related infections found that at the end of follow-up, older patients had significantly worse functional outcome and impaired physical quality of live, as well as more frequently higher rates of persistent infections, and multidrug resistance than those associated with infections in younger patients [4]. In addition to increased patient morbidity and mortality, they entail a significant economic burden. Recent studies show that median costs per patient are six to seven times higher in infected than in uninfected patients, being increased length-of-stay the most important driver for total healthcare costs [5, 6].

The treatment of IOM, as well as the treatment of other osteoarticular infections with prolonged treatment, involves excessive consumption of antimicrobials, with inadequate prescriptions and prolonged durations that favors superinfections by multidrug-resistant microorganisms, in addition to drug related toxicities, especially diarrhea due to *Clostridioides difficile* [7]. To the best of our knowledge, there are no well-designed studies comparing the best antibiotic treatment regimen and duration in IOM when implant is retained. The management of this infection is mainly based on extrapolations from prosthetic joint infections (PJI), tradition and personal experiences, which cause a substantial heterogeneity among institutions and countries. In IOM, most studies published so far involved few patients, mixed different orthopedic devices, and included a wide variety of fractures. A retrospective case-control study performed in patients with PJI and IOM after fracture failed to demonstrate that a longer duration of antibiotic treatment is associated with a lower rate of reinfection one year after DAIR (debridement, antibiotic and implant retention) [8]. A meta-analysis including one clinical trial and 9 observational studies concluded that when treating PJI patients following DAIR, an 8-week course of antibiotic therapy for total hip arthroplasty and a 75-day course for total knee arthroplasty may be a safe approach [9]. In a later randomized clinical trial [10] comparing 6 weeks oral vs. 6 weeks intravenous therapy in osteoarticular infections, global failure rate in the 247 patients managed with implant retention (including prostheses) was 19.5%. However, prolonged antibiotic regimens are used in IOM, usually 3 months or more. In two papers published by the same group, the success rate when DAIR was performed under optimal conditions was high, with a duration of antibiotic therapy of about 12 weeks [11, 12]. This was endorsed by a recent meta-analysis including 276 patients with IOM which found a cure rate of 86% when DAIR was performed <3 weeks after material implantation, among 82–89% when DAIR was performed in infections between 3–10 weeks, and 67% in infections of more than 10 weeks. But their heterogeneity was high, with a higher duration of antibiotic therapy, always above 12 weeks, regardless of the time of infection [13].

In conclusion, there were not clinical trials evaluating the optimal duration of antibiotic therapy in IOM when implant is retained. Because there are antibiotics that have proven to be effective for the treatment of infection associated to implant, mainly in PJI, these antibiotics could be used in these infections. Investigating whether shorter duration of treatment is a priority in infectious diseases, as a way to reduce the exposure to antibiotics and help in controlling antimicrobial resistance and avoiding unnecessary adverse events and cost.

The objective of this article is to describe the hypothesis, objectives, design, variables and procedures for a pragmatic randomized controlled trial comparing different durations of antibiotic treatment in IOM after long bone fractures treated with debridement and implant retention.

## Materials and methods

### Hypothesis and objectives of the trial

The hypothesis of the study is that in IOM of long bone fracture, a short antibiotic treatment is equally effective as a long antibiotic treatment in patients selected for debridement and implant retention. Antibiotic duration can be selected according to the time of diagnosis of IOM as it takes into account the biofilm formation and the state of fracture consolidation:

- In patients with early infections (those that occur in the first 2 weeks after implantation of the osteosynthesis material) in whom early debridement is performed, it is possible to shorten the duration of antibiotic treatment to 8 weeks (vs. 12 weeks);
- In patients with delayed infections (those that occur between 3 and 10 weeks after implantation of the osteosynthesis material) in whom it is not possible to remove the implant due to instability/lack of fracture healing, it is possible, after surgical debridement, to shorten the duration of antibiotic treatment to 12 weeks (vs. maintaining antibiotic treatment until fracture healing or implant removal).
- In patients with late infections (those that occur more than 10 weeks after implantation of the osteosynthesis material) healing is not possible until the implant is removed, but when removal is not possible, antibiotic therapy can be maintained until fracture healing or implant removal.

The main objective of this trial is to evaluate whether, after performing surgical debridement in patients with IOM due to a large bone fracture, a short antibiotic treatment is as effective as a longer treatment. Secondary objectives are: a) to evaluate the efficacy and safety of different antimicrobials used in IOM, b) to evaluate the development of antimicrobial resistance during antibiotic treatment, c) to evaluate the need for new surgeries during follow-up, d) to provide information about the functional prognosis and quality of life of the patient according to each of the treatment strategies, e) to evaluate the consumption of health resources with each type of strategy, and f) to evaluate the different reconstruction strategies (bone and soft tissue) carried out in order to recover lost functionality (degree of mobility and autonomy).

### Trial design, sites and study period

DURATIOM is designed as a prospective, multicenter, non-inferiority, open labelled, pragmatic randomized clinical trial comparing different durations of antibiotic therapy in IOM after long bone fractures treated with debridement and material retention. A 36-month recruitment period is planned. The study is coordinated from Hospital Universitario Virgen Macarena and sponsored by Fundación Pública Andaluza para la gestión de la Investigación en Sevilla (FISEVI). Twenty-six public Spanish hospitals where trauma surgery is performed will participate (Table 1). Each hospital has a research team that must include at least one trauma surgeon and a specialist in infectious diseases or internal medicine. Currently, the management of osteoarticular infections is very similar in all the participating centers, since the researchers work together in study groups and consensus documents.

**Table 1:** Spanish hospitals participating in DURATIOM.

N.	Region	Principal Investigator	Hospital
1	ANDALUCÍA	Dr. Mª Dolores del Toro	Hospital Universitario Virgen Macarena, Sevilla
2	ANDALUCÍA	Dr. José Manuel Lomas	Hospital Universitario Virgen del Rocío, Sevilla
3	ANDALUCÍA	Dr. Juan E. Corzo	Hospital Universitario Virgen de Valme, Sevilla
4	ANDALUCÍA	Dr. Beatriz Sobrino	Hospital Regional Universitario de Málaga, Málaga
5	ANDALUCÍA	Dr. Alfonso del Arco	Hospital Universitario Costa del Sol, Marbella, Málaga
6	ANDALUCÍA	Dr. Enrique Nuño	Hospital Universitario Virgen de la Victoria, Málaga
7	ANDALUCÍA	Dr. Alberto Romero	Hospital Universitario de Puerto Real, Cádiz
8	ARAGÓN	Dr. José Ramón Paño	Hospital Clínico Universitario Lozano Blesa, Zaragoza
9	BALEARES	Dr. Helem H. Vílchez	Hospital Universitario Son Espases, Palma de Mallorca, Mallorca
10	CANTABRIA	Dr. Marta Fernández	Hospital Universitario Marqués de Valdecilla, Santander
11	CASTILLA Y LEÓN	Dr. Alberto Bahamonde	Hospital Universitario del Bierzo, Ponferrada, León
12	CATALUÑA	Dr. Óscar Murillo	Hospital Universitari de Bellvitge, Barcelona
13	CATALUÑA	Dr. Natividad de Benito	Hospital Universitari de la Santa Creu i Sant Pau, Barcelona
14	CATALUÑA	Dr. Mª Dolores Rodríguez	Hospital Universitari Vall d’Hebron, Barcelona
15	CATALUÑA	Dr. Luisa Sorlí	Hospital del Mar, Barcelona
16	CATALUÑA	Dr. Eva Van den Eynde	Hospital Universitari Parc Taulí, Sabadell, Barcelona
17	CATALUÑA	Dr. Esteban Alberto Reynaga	Hospital Universitario Germans Trias i Pujol, Badalona, Barcelona
18	CATALUÑA	Dr. Laura Morata	Hospital Clinic i Provincial de Barcelona, Barcelona
19	GALICIA	Dr. María José García	Hospital Universitario Lucus Augusti, Lugo
20	LA RIOJA	Dr. José Ramón Blanco	Hospital San Pedro, Logroño, La Rioja
21	MADRID	Dr. Jaime Lora-Tamayo	Hospital Universitario 12 de Octubre, Madrid
22	MADRID	Dr. José Mª Barbero	Hospital Universitario Príncipe de Asturias, Alcalá de Henares, Madrid
23	MADRID	Dr. Javier Cobo	Hospital Universitario Ramón y Cajal, Madrid
24	MADRID	Dr. Antonio Blanco	Hospital Universitario Fundación Jiménez Díaz, Madrid
25	MADRID	Dr. Alicia Rico	Hospital Universitario La Paz, Madrid
26	PAÍS VASCO	Dr. Laura Guio	Hospital Universitario de Cruces, Baracaldo, Vizcaya

The trial obtained competitive, public funding from the Spanish Ministry of Health (Instituto de Salud Carlos III, reference ICI21/00014, www.isciii.es). Funding for the trial was communicated on November 2021, available for study expenses in January 2022. The funder will not have a role in study design, data collection and analysis, decision to publish, or preparation of the manuscript. The study is supported by the Spanish Clinical Research Network (SCReN) from the Spanish Ministry of Health (Instituto de Salud Carlos III, PT20/ 00123) with the internal study code 21.009. The study is registered at ClinicalTrials.gov (identifier, NCT05294796) on Jan 26^th^ 2022 and at the European Union Drug Regulating Authorities Clinical Trials (EUDRACT) (identifier, 2021-003914-38) on Jul 16^th^ 2021 (S1 File).

The study protocol adheres to the SPIRIT 2013 guidelines for clinical trials and the Statement Consolidated Standards of Reporting Trials (CONSORT) for pragmatic designs (S2 File). Trial authorization by the Spanish Agency for Medicines (AEMPS) was obtained on Jul 27th 2022 (version 2.0 dated Jun 8th 2022) and any modifications to the protocol will be reported to the clinical research ethics committee, AEMPS, and Health Products and ClinicalTrials.gov, and will be stated in the final manuscript. We plan to grant public access to the full protocol, participant level data, and the statistical code if required.

### Study population

The study target population is formed by patients older than 14 years, with IOM implanted after a long bone fracture diagnosed during the study inclusion period, and who are treated by DAIR.

IOM is considered when at least one microbiological and one clinical criterion are met:

- Microbiological criterion: isolation of the same microorganism (same species and antibiogram) in ≥2 tissue samples taken intraoperatively (≥1 in the presence of virulent microorganisms such as *Staphylococcus aureus*) or from the combination of cultures taken preoperatively by deep aspirate plus intraoperative cultures.
- Clinical criteria:
  - Presence of clinical syndrome comprising any of the following: localized pain, localized redness or edema, temperature >38.0°C, or discharging wound.
  - Presence of a sinus tract communicating with the osteosynthesis material.
  - Presence of pus around the implant.
  - Presence of histopathological signs of acute inflammation in the peri-implant tissues taken at the time of debridement.

#### Inclusion criteria

Patients are eligible for enrollment in the study if they meet all the following inclusion criteria:

1. Age ≥ 14 years at the time of IOM diagnosis.
2. Has a stabilized fracture, even if unconsolidated.
3. Controlled infection, that is, absence of signs or symptoms of sepsis. Sepsis is defined as the presence of more than 2 points on the qSOFA or SOFA scale [14].
4. Early IOM (those that occur in the first 2 weeks from the implant surgery) or delayed IOM (those that occur between 3 and 10 weeks from the implant surgery).
5. Availability of antibiotics active against the isolated microorganism.
6. Absence of bone exposure. Patients who initially had bone exposure but during the debridement surgery bone coverage was performed by any method (skin approximation, grafting, vacuum therapy) can be included in the criteria.
7. Have signed the informed consent form; in the case of minors, signature of the legal guardians and assent of the minor.
8. If there is a possibility of pregnancy (in women of childbearing age) or paternity, to accept the use of a highly effective birth control method recommended by the Clinical Trial Facilitation Group (CTFG): https://legemiddelverket.no/Documents/Godkjenning/Klinisk%20utpr%C3%B8ving/2014_09_HMA_CTFG_Contraception_guidance%20Version%201.1.pdf) during the treatment phase of the trial.

#### Exclusion criteria

A patient will be excluded from the study if any of the following scenarios are present:

1. Late infection (those that occur more than 10 weeks from implant surgery).
2. IOM in a fracture of a bone other than long.
3. Infection of revision osteosynthesis material or occurring after previous surgeries.
4. Unlikely to complete follow-up for at least 1 year after antibiotic treatment ends.
5. Pregnant or breastfeeding women.
6. There may be drug interactions or contraindications described in the data sheets of the drugs used in this trial.
7. Infections caused by mycobacteria, fungi and parasites (since these infections are treated with different drugs and different durations).
8. Debridement involves replacement of all the osteosynthesis material in the same surgical time (since these patients require a duration of antibiotic treatment of less than 8 weeks in all cases).
9. Infection associated with external fixation.

### Patient selection and enrolment

Candidate patients will be detected by daily review of admissions to the Traumatology Units both from the Emergency Departments and from outpatients, as well as in the daily ward rounds of the Traumatology wards and outpatient clinics. Screening procedures to select the accurate candidate for the study will involve the Traumatology team, the nursing team, and the Infectious Diseases/Internal Medicine team. Patients will be considered for inclusion after review by the principal investigators of the team.

Informed consent will be obtained from each participant by good clinical practice (GCP) trained research staff after assessing the patient information sheet. Participation in the study is voluntary and patients can give up their willingness to participate in the study at any time with no changes in the clinical attendance after the decision. Otherwise, the sponsor decision of subject withdrawal from the study are the following; if a major protocol violation occurred, in case of clinical failure, or according to safety criteria.

### Randomization

Patients will be randomized once inclusion and exclusion criteria are checked, and informed consent is signed; randomization must be performed no later than 72 h after knowing the microorganism causing the IOM and the susceptibility results, preferably from samples obtained in DAIR.

Patients signing the informed consent form will be centrally randomized using an automated online system with a 1:1 randomization. Randomization will be stratified according to whether they are early IOM (those that occur in the first 2 weeks from the implant surgery) or delayed IOM (those that occur between 3 and 10 weeks from the implant surgery), and using blocks of 20 patients. The randomization list will be obtained using Epidat 4.0 software. The automatic randomization system is integrated in the electronic case report form (e-CRF) of the study. One written copy of the randomization list will be stored at the Clinical Trials Unit of the Hospital Universitario Virgen del Rocío (UEC-HUVR) in the event that technical issues avoid the automatic system working, and the assignation of study intervention will be obtained by phone call.

### Interventions and study treatment

Patients will be classified according to the age of the osteosynthesis material implantation, i.e., according to the time from implant surgery to the diagnosis of IOM, as it takes into account the biofilm formation and the state of fracture consolidation [2]:

- Early IOM: those that occur and are diagnosed in the first 2 weeks from the implant surgery.
- Delayed IOM: those that occur and are diagnosed between 3–10 weeks from the implant surgery.

Patients with early or delayed infections who meet the inclusion criteria and were managed with DAIR will be allocated to one of the following arms (S3 File):

- Experimental arm: short-term antibiotic treatment; 8 weeks in early IOM or 12 weeks in delayed IOM.
- Control arm: long-term antibiotic treatment; 12 weeks in early IOM or antibiotic until fracture healing or implant removal in delayed IOM.

The limit to consider an IOM as early or delayed is given by the moment in which the DAIR is performed. In other words, an IOM is considered early when 14 days or less have elapsed between the date of the implant surgery and the DAIR, and delayed when 3 to 10 weeks have elapsed between the date of the implant surgery and the DAIR.

Antibiotics administered after DAIR will be carried out empirically according to the protocols of each hospital. The duration of intravenous treatment will be at the discretion of the investigator, but it is advised that in the absence of sepsis or post-surgical complications it should not exceed 7 days. Antibiotics administered before DAIR will be permitted in both groups but will not be governed by the trial protocol. Once intravenous treatment is considered to be discontinued, the antibiotic treatment administered orally will be agreed upon by the Traumatology-Infectious Diseases/Internal Medicine-Microbiology team. Given the absence of controlled studies carried out specifically on IOM, it will be performed according to the Spanish prosthetic joint infection guidelines [15]. Adjunctive oral agents such as rifampin will be permitted at the infection specialist’s discretion, reflecting usual practice. In the switch to the oral route, antibiotics with good bioavailability should be used [15], and if no options are available outpatient parenteral antimicrobial therapy may be used. In case of intercurrent infection unrelated to IOM, up to 7 days of concomitant antibiotic treatment is allowed.

Rescue medication is not foreseen. Since the antibiotics to be used are those of standard practice, if the antibiotic initially administered is not effective or an adverse effect occurs, it can be changed according to clinical guidelines or standard practice, without this being considered a failure or violation of the protocol. The alternative may be intravenous treatment on an outpatient or inpatient regimen. If there is no alternative and the antibiotic treatment must be withdrawn, the patient will be dropped from the study, but will continue to be followed according to the protocol and will be included in the intention-to-treat analysis. Changes in treatment, the reason, and adverse reactions will be recorded in the CRF.

Adherence to oral antibiotic treatment will be measured by registering the medication given with expiration day and official batch number in each delivery of drugs.

### Minimizing bias

In order to inform about the generalizability of the results, all patients diagnosed of IOM in the participating sites will be evaluated and the reasons for not being included will be collected as “screening failures”. The study is conceived as open-labelled due to the logistic and budget limitation for masking a great group of drugs needed for this study. Blinding will not be used; to avoid bias in the assessment of endpoints, a blinded investigator from the coordinating hospital (HUVM) will also evaluate the outcomes based on data collected.

To control selection bias, it has been developed: a) a randomization procedure that will be carried out when the patient is evaluated by means of an automatic system, so investigator is blinded to the assignment of treatment arm until the procedure is completed; b) stratification according to infection type (early/delayed); c) central randomization distributed in 1:1 ratio using blocks into participating sites.

The potential effect of residual confounders will be controlled by performing a multivariate logistic regression analysis; because the type of surgical treatment and the promptness with which it is performed may vary across sites, the sites will be classified into those with high and low cures rates as a potential confounder. Antibiotic therapy may also vary, such as the tendency to prolonged intravenous treatment. In order to standardize practice and variables collection, investigating teams will be trained at each center.

### Outcome variables

Endpoints will be identified by prospective follow-up of patients for at least 12 months after antibiotic therapy withdrawal.

- **Primary outcome**

The primary outcome will be the composited variable "cure” including:

- clinical cure in the test of cure (TOC) (see definition below);
- radiological healing;
- definitive soft tissue coverage at TOC.

The TOC will be performed 12 months after the end of antibiotic therapy prescribed to treat IOM following DAIR.

Clinical cure is defined by the presence of any of the following:

- The absence of clinical signs and symptoms of infection (persistence of symptoms of infection, relapse of infection after a period without symptoms, or superinfection by a different microorganism) without antibiotic therapy and with CRP <10 mg/L (unless another cause justifies a higher CRP value);
- No need for chronic suppressive antibiotic therapy to "control" of the infection.

Persistence of infection is defined as no disappearance of clinical signs and symptoms of IOM, produced by the same micro-organism that produced the initial infection.

Relapse of infection is defined as the reappearance of signs or symptoms of IOM after a period without symptoms, by the same micro-organism that produced the original infection.

Superinfection is defined as a re-infection produced with a different micro-organism than the one that caused the initial infection.

Radiological healing is defined as the presence of radiological signs of fracture consolidation (plain radiographic or computerized tomography) of the infected bone. Non-union or absence of consolidation of a fractured bone is defined when it has not completely healed within 9 months after osteosynthesis surgery or when it has not shown progression towards fracture callus formation in 3 consecutive months on serial radiographs [16]. Given the subjectivity that can sometimes occur in the assessment of bone healing criteria, a blinded investigator will evaluate the concordance of clinical and radiological healing criteria performed by the local investigator. The REBORNE bone healing score will be used to perform a detailed evaluation of long bone non-union consolidation in radiographs and CTs if it is necessary [17].

Clinical failure occurs when any of the following is present:

1. Absence of clinical and microbiological cure of the IOM.
2. Absence of radiological healing.
3. Exposure of bone and/or osteosynthesis material.
4. Death related to the infection.

If the patient dies from a cause unrelated to the infection, it will be considered lost to follow-up or censored.

The main outcome variable will also be evaluated by subgroups:

- Type of infection: early and delayed.
- Type of fracture: open (Gustilo and Anderson classification) or closed.
- Type of osteosynthesis material: endomedullary nail or needle, plates or screws.
- According to the etiology of the infection: infections by different microorganisms, infections by multidrug-resistant microorganisms.
- According to type of patient: comorbidities, elderly, renal insufficiency, gender.

- **Secondary outcomes**

**a) Clinical endpoints:** efficacy of each group of antibiotics, secondary infections, infection recurrence rate (relapses and reinfections), need for new surgeries (debridement, material removal, covering, amputation), reconstructive surgery strategies (bone and soft tissue), functional status (defined as restoration of limb function prior to fracture) and state of health.

The assessment of the patient’s functional status is complex given the number of fractures and bones involved. To facilitate it, the following classification of functional status (normal o reduced with respect to the situation prior to fracture) has been chosen to be performed at TOC:

- For lower limbs mobility functional status will be classified as walks unaided, with 1 cane, with 2 canes, with walker, "home alone ambulation" and no ambulation.
- For upper limbs mobility functional status will be used the Quick-DASH questionnaire [18].

To assess generic health outcomes from the patient’s perspective we will used the SF-12 questionnaire or Barthel in people over 70 years of age [19, 20].

**b) Microbiological endpoints:** Resistance development to antibiotic used during therapy and *C*. *difficile* infection during therapy and at 30-day.
**c) Adverse events (AE) and complications:** Adverse events and severity, including death (that is, all-cause mortality).
**d) Consumption of health resources:** Consumption of health resources will be evaluated for each type of strategy: days of antibiotic treatment, length of hospital stay, readmissions, number and type of surgeries performed.

### Visits and follow-up of participants

**Fig 1:**
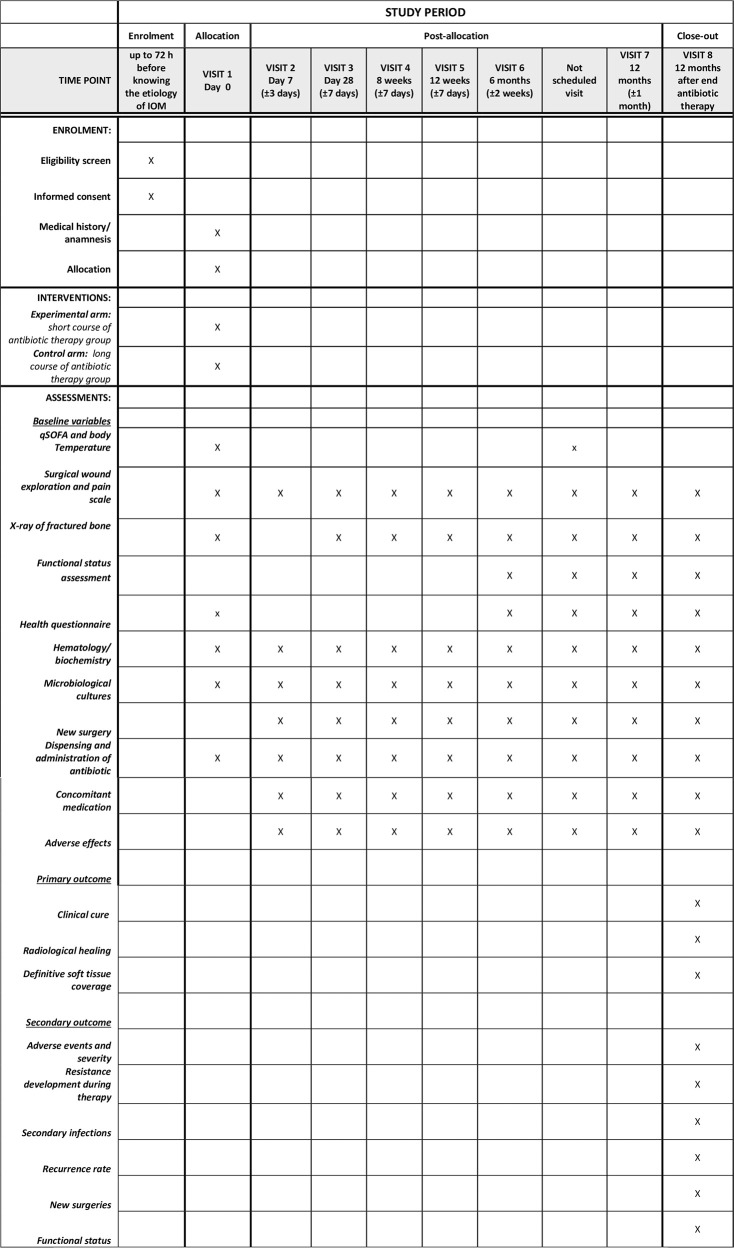
Visits and follow-up of participants of DURATIOM trial. The total follow-up time will be one year after completing the antibiotic treatment prescribed after randomization. Patients with delayed infections randomized to the long-treatment regimen (until fracture union) who had undergone 6 months or more of antibiotic treatment will be evaluated 18 months after the start of treatment (or randomization).

- All visits and procedures to be performed at each one are specified in Fig 1.
- All patients will be assessed:
- The day of recruitment/randomization (day 0, visit 1).
- The day of switching from intravenous to oral treatment (day 7±3, visit 2) or at discharge if the patient goes home with oral antibiotic.
- At 1 month (day 28±7, visit 3), for safety of treatment.
- At 8 weeks (±7 days) (visit 4) for withdrawal of antibiotic treatment in the experimental arm of early infections.
- At 12 weeks (±7 days) (visit 5) for withdrawal of antibiotic treatment in the experimental arm of delayed infections and in the control arm of early infections.
- At 6 months (±14 days) (visit 6), for evaluation of patients who completed treatment and for assessed the control arm of delayed infection who are still on antibiotic treatment.
- At 12 months (±1 month) (visit 7), for evaluation only of patients in control arm with delayed infections.
- At 1 year after completion of antibiotic treatment (±1 month) (TOC, visit 8), all patients.
- If any event occurs between visits (relapses, new hospitalization or surgery, adverse effects, antibiotic changes), additional visits will be performed and collected.

### Microbiological studies

Preoperative wound aspirate and intraoperative tissues specimen will be sent to microbiology laboratory. For intraoperative will be required at least three biopsies from bone- or peri-implant tissue. Tissue specimens will be cultured for 7–10 days at 35°C on aerobic and anaerobic blood agar as well as in thioglycolate broth. Samples may be incubated for up to 14 days in agreement with the clinician and the microbiologist in case of suspected infection by fastidious micro-organisms or in patients with previous anti-microbial treatment. In case of any orthopaedic devices is removal (e.g. a loose screw from a plate), it will be additionally sonicated and sonication fluid will be cultured [21]. Standard microbiological techniques will be used to identify microorganisms recovered and to determine antimicrobial susceptibility. In case of diarrhea during the antibiotic treatment and up to one month after its completion, stool culture and detection of *C*. *difficile* toxin in stool shall be performed.

### Radiological and laboratory studies

Blood tests and radiological studies will be performed at the initial visit and subsequently as specified in the visit schedule (Fig 1). Laboratory tests will be performed at each center in accordance with the usual clinical practice standards. The following tests will be performed:

- Blood count including leukocyte, neutrophil, haemoglobin and platelet counts.
- Blood biochemistry including sodium, potassium, creatinine, C-reactive protein, AST (GOT), ALT (GPT) and total bilirubin (if altered also direct bilirubin).

### Sample size

To our knowledge, no previous trials on IOM treatment had been performed; a meta-analysis of observational studies found a clinical cure of IOM when DAIR was performed (regardless antimicrobial therapy duration) of 86–100% in early IOM, and 82–89% in delayed IOM. Although our primary outcome is a composite outcome including clinical cure, radiological healing and soft tissue coverage which might reduce the cure rate, but the fact that in this randomized trial we will only include patients in whom a debridement is performed, we estimated that 85% of patients in both arms will reach that primary outcome. With a 10% non-inferiority margin, a 5% level of significance (α value) set at 5%, 80% power, and 15% loss rate, we would need to recruit 182 patients per arm (364 in total). Subgroups analyses for the primary outcome as defined above will be exploratory, as demonstration of non-inferiority in each of them is unfeasible.

Interim analysis will be conducted when 75 patients have been included to assess the frequency of the event rate and inclusion rate to adjust the intended size of the study population.

### Statistical analysis

The populations for analysis will be:

- Intention-to-treat (ITT) population: all appropriately-included and randomized patients.
- Per protocol population (PP): all patients who received the complete antibiotic treatment regimen according to the inclusion group.
- Clinically evaluable (CE) population: all patients with evaluation of success in the cure test at 12 months after completion of treatment, or who have clinical failure before TOC.

The primary analysis will be the absolute comparison with one-sided 95% confidence interval of the proportion of patients reaching the composited primary outcome variable "cure" in the ITT population. Secondary analysis will include subgroups analyses for the primary outcome using the same procedure, and the comparison of the composite variable components (clinical cure, radiological healing and soft tissue coverage) and secondary variables in the ITT, PP and CE populations, after excluding incorrectly assigned patients. For continuous secondary outcomes, parametric and nonparametric tests will be used as appropriate.

Finally, a multivariate analysis will be performed in order to control the potential residual effect of variables other than duration of antibiotic therapy on the primary outcome by logistic regression. Potential confounders will be identified by bivariate analysis of their associations with the primary outcome, considering a *p* value <0.15, and will be entered into the multivariate model to check if the effect of the duration of therapy is significantly modified. Expected potential confounders include age, Charlson, delay in DAIR, type and location of fracture, type of osteosynthesis material, microorganism, and antibiotic group. The effect of study sites will be explored by classifying the sites as those with higher and lower cure rates, and including it as a potential confounder. A formal statistical planning has been prepared before the initiation of the study, with all the details, variables, and statistical test to be used during the study (S4 File).

### Safety and adverse event reporting

Safety for all antibiotic used will be carefully followed according to Regulation (EU) No. 536/2014 and Royal Decree 1090/2015. For this objective a clear definition of adverse events, serious adverse events and serious and unexpected adverse events will be included in the final protocol for the study; the accomplishment of serious criteria will be followed by additional information to be completed and sent to the Pharmacovigilance department of UICEC-HUVR which will be acting in representation of the sponsor for the pharmacovigilance activities that includes the evaluation of casualty and the expedited notification to regulatory Authorities depending on the final classification of the event. Cases of relapse of infection that meets seriousness criteria and cases of readmission for relapse of infection has been approved for a waiver for expedited reporting. Yearly reports on safety will be issued in a data safety updated report to be send to Ethics committees and AEMPS.

The antibiotics to be used are standard practice in osteoarticular infections, but due to alerts from the Food and Drug Administration (FEA) and the AEMPS, especially with quinolones and even for the specific considerations of pharmacovigilance activities within the performance of a clinical trial, all the Summary of Characteristic Products will be used as a reference information, taken specially consideration for regulatory agencies safety alerts.

### Data and safety monitoring

An external data safety monitoring board formed by 3 expert members not participating as investigators in the project will independently evaluate the data when the first 75 patients have been enrolled and monitored to ensure that there are no safety or efficacy reasons to stop the trial. The safety committee will make recommendations after the interim analyses. A charter with the formal activities to be performed will be included in the sponsor documents. A report elaborated with data on recruitment, adverse events notified to the pharmacovigilance center for the study, and data of primary and secondary outcomes will be provided to the committee before the meetings. The Data and Safety Monitoring Board can recommend that the current study continue without modification, continue with specified modifications, discontinue the study or halt or modify the study until more information is available.

### Quality assessment

Clinical Trials Unit of the Hospital Universitario Virgen del Rocío (UEC-HUVR) and the study team will coordinate the activities in collaboration with the ISCIII Clinical Research Network (PT20) for authorization of the study, sites activation and training and for the performance of monitoring activities for the entire duration of the study. A monitoring planning will be approved for the follow-up of correct data collected according to approved protocol and based in the source data available for each patient. Local investigators will need to have accredited training in Good Clinical Practice or Good Clinical Laboratory Practice, as appropriate; the local teams will be adequately trained before the study initiation in a formal site initiation visit performed by the UICEC-HUVR-PT team, on which all the procedures for the study, timelines, eCRF functioning and Pharmacovigilance tasks will be documented. On site monitoring will be performed by trained monitors. A specific monitoring plan will be developed with the indication of the number of on-site visits to be performed during the study and with the percentage of source document verification to be performed in total, the time for completing the data on the eCRF and even for the monitoring report to be issued.

The coordination team of the study will establish a quality system of follow-up, with the continuous surveillance of events happening during the study to be discussed for resolution in their formal meetings. Standard operating procedures for activities as monitoring or pharmacovigilance will be used during the complete study.

To ensure the participation and follow-up of the centers during the development of the trial, a weekly reminder will be sent with the cases included and the screening failures, and a new monthly letter with a summary of the progress of the trial.

### Ethical issues and dissemination

Trial approval by the Provincial Ethic Committee of Seville was obtained on Jul 7th 2022 (version 2.0 dated Jun 8th 2022). An approved informed consent (version 2.0, dated Jun 8^th^ 2022) form must be signed before any study specific procedures is performed. As patients with 14–18 years old can participate, a specific information sheet adapted for minors is included, approved with the same version and date. The study will be carried out under the ethical principles regarding human experimentation developed for the medical community by the World Medical Association (WMA), declaration of Helsinki and following all the applicable legislation regarding the realization of clinical trials in the European Union (EU No 536/2014) and Spanish Royal Decree 1090/2015. The study will not start until the approval of the AEMPS and the Ethic committees give their authorization. The results of the study will be submitted for publication to a scientific journal following the CONSORT recommendations.

## Discussion

There are no clinical trials evaluating the optimal duration of antibiotic therapy in IOM when implant is retained. Because there are antibiotics that have proven to be effective for the treatment of infection associated to implant, mainly in PJI, these antibiotics could be used in these infections. Investigating whether shorter duration of treatment is a priority in infectious diseases, as a way to reduce the exposure to antibiotics and help in controlling antimicrobial resistance and avoiding unnecessary adverse events and costs.

A noninferiority approach is justified because the use of shorter courses of treatment would benefit patients by reducing the selective pressure of broad-spectrum antibiotics commonly used in these infections. A superiority trial could be done by using a composite primary outcome including for example colonization and/or superinfection by multidrug-resistant bacteria or severe adverse effects, but it would need a very high sample size that would make it unfeasible.

It is well known that classical trials may not adequately inform practice as they are typically performed at selected sites with highly experienced investigators and selected participants not really representing most patients to whom the results would be extrapolated, so they could be overestimating benefits and underestimating harm. This led to the idea that more pragmatic trials showing the real-world effectiveness of the intervention in broader patient groups, are required [22]. This may be particularly important in the evaluation of antibiotics as the outcome of the infection do not only depends on the treatment itself but on features of the patients, the severity of the infection, the type of the fracture, the microorganism and different aspect of the surgical management (debridement, implant removal, soft tissue coverage…). Therefore, we propose a pragmatic trial. We used the PRECIS-2 tool to evaluate the level of pragmatism of our design [23] and followed the recommendations of extension of the CONSORT document for this type of trials [24].

To determine the duration of antibiotic treatment we decided to classify IOM according to the age of the implant or onset of infection symptoms, as it takes into account the biofilm formation and the state of fracture consolidation [2]. Briefly, this IOM classification includes: a) Early infection (occurs in the first 2 weeks after implant surgery): bacteria may already have formed a biofilm, although this biofilm may still be in an “immature” phase, the bone does not show signs of osteomyelitis or osteolysis despite the presence of bacteria, bone healing is in the “inflammatory or soft callus stage”; b) Delayed infection (occurs between 3 and 10 weeks after the implant surgery): biofilms is mature and more resistant to antibiotic therapy, although normal bone healing takes up to 10 weeks (“hard callus stage” is situated between 3 and 16 weeks), presence of bacteria weakens callus formation. In this phase often occurs bacterial bone invasion and inflammation (“osteomyelitis”); c) Late infection (occurs after 10 weeks of implant surgery): primarily caused by micro-organisms of low virulence like *S*. *epidermidis*, although bone healing may have taken place in some cases, severe inflammation and osteolysis with osteomyelitis lead to instability of the osteosynthesis. In contrast to PJI, in IOM the primary objective is not the complete eradication of the infection, but the healing and stabilization of the fracture, as the material can be removed once the fracture has healed. Successful management of IOM includes fracture consolidation, restoration of the soft tissue envelope, return to function, prevention of residual chronic infection and eradication of infection. The surgical strategy will therefore depend on the onset of symptoms and the healing and stabilization of the fracture, although the type of implant (plate, nail…), the location of the fracture (diaphyseal, articular), the condition of the skin and soft tissues, and the patient’s situation play an important role. In early infections, consolidation can be achieved without removal the material if the osteosynthesis is stable. In these cases, a thorough surgical debridement should be performed, with sampling for microbiology, followed by empirical antibiotic therapy and then targeted at the isolated microorganisms. The duration of antibiotic treatment is unclear, and although 12 weeks is recommended based on a low-quality clinical trial (18), it will depend on early diagnosis and debridement, the severity of the infection, the patient’s condition, and the skin and soft tissue status. Based on PJI studies, 8 weeks of antibiotic treatment seems to be sufficient time in case of debridement [25]. In delayed infections, when removal of the material is not possible because the fracture is not healed, and provided the fracture is stable and debridement is performed to remove necrotic tissue, haematomas and decrease the bacterial load, 12 weeks of treatment may be sufficient. Late infections without removal the implant are likely to require suppressive treatment until the fracture has healed or until the material can be removed [3].

Given the absence of controlled clinical trials with antibiotics specifically designed for IOM, the antibiotics used in this study will be those used in routine practice for osteoarticular infections, with the Spanish consensus document on the management of PJI serving as a guide [15]. The fact that antibiotic treatment is chosen by the treating physician introduces a challenge for the analysis. However, there is already experience in such approach, e.g., in oral switch in osteomyelitis or endocarditis (OVIVA and POET trials) [10, 26]. Another critical aspect of this study is close communication with the microbiology laboratory in order to target antimicrobial therapy.

We decided to use a composite primary endpoint to include very relevant variables (clinical cure, radiological healing, definitive soft tissue coverage) which address overall recovery from infection, but we excluded the variable functional status because it may not always be related to infection.

### Strengths and limitations of this study

The design of this randomized study has limitations, including the open design, the heterogeneity of patients, type and fracture location, the antibiotic treatment, the clinical practice between centers, and the subjectivity in the assessment of healing criteria and functional status. The sample size will not allow to demonstrate the non-inferiority of shorter treatment in subgroups of patients. Other risks include reaching the sample size and loss to follow-up due to the long evaluation time.

Some strengths are its pragmatic design which we hope will allow the appropriate representation of patients with the target infections, the multicenter participation, and previous experience in trials in which the antibiotic used is that of standard practice.

## Conclusions

Research into this manage of IOM has not been given much importance for years despite the great impact on health and the health system and has hindered the development of well-designed clinical trials. The expected impact of this study can be a change in clinical practice; if the hypothesis of non-inferiority of short vs. long antibiotic treatments is demonstrated, and the efficacy of antibiotics with less ecological impact in long treatments, the impact on reduction of bacterial resistance, toxicity and health costs will be observed. The project also aims to promote quality and multidisciplinary care, homogenizing clinical practice in the management of IOM based on scientific knowledge. Early and personalized patient care with IOM allows for faster recovery, both clinically and functionally, enabling earlier mobility recovery.

## Supporting information

S1 File Protocol version 2.1 dated Aug 4^th^ 2022. This document contains the updated version of the protocol approved by the AEMPS and the Provincial Ethic Committee of Seville.
(PDF)

S2 File SPIRIT 2013 checklist. This document contains recommended items to address in a clinical trial protocol and related documents.
(DOC)

S3 File Design algorithm, inclusion criteria, randomization, and outcome of infection associated with osteosynthesis material (IOM). This document contains the algorithm with the inclusion criteria, randomization and outcomes of infection associated with osteosynthesis material.
(PDF)

S4 File Statistical analysis version 3 dated Feb 16th 2023. This document contains the statistical analysis plan.
(PDF)

S1 Fig Design algorithm, inclusion criteria, randomization and outcome of infections associated with osteosynthesis material (IOM). (DOCX)
