# Supplementary material for: Efficacy and safety of different antimicrobial DURATions for the treatment of Infections associated with Osteosynthesis Material implanted after long bone fractures (DURATIOM): Protocol for a randomized, pragmatic trial
Source: PLoS One. 2023 May 22;18(5):e0286094. doi: 10.1371/journal.pone.0286094 (PMC10202272; doi:10.1371/journal.pone.0286094)
Supplement: S3 File — This document contains the algorithm with the inclusion criteria, randomization and outcomes of infection associated with osteosynthesis material. (PDF) [file pone.0286094.s003.pdf]

STATISTICAL ANALYSIS PLAN

EFFICACY AND SAFETY OF DIFFERENT ANTIMICROBIAL DURATIONS FOR THE TREATMENT OF INFECTIONS ASSOCIATED WITH OSTEOSYNTHESIS MATERIAL IMPLANTED AFTER LONG BONE FRACTURES (DURATIOM): PROTOCOL FOR A RANDOMIZED, PRAGMATIC TRIAL.

ACRONIMUM: DURATIOM

Nº EUDRACT: 2021-003914-38

VERSION 3.0. 16 February 2023

1. INTRODUCTION

This is the Statistical Analysis Plan (SAP) for the “DURATION” trial, a multicenter, non-inferiority, open labelled, pragmatic randomized clinical trial comparing different durations of antibiotic therapy in infection of osteosynthesis material (IOM) implanted after long bone fractures and treated with debridement and material retention.

This SAP was developed before the initiation of the study.

1.1 Study hypothesis

The hypothesis of the study is that in IOM of long bone fracture, a short antibiotic treatment is equally effective as a long antibiotic treatment in patients selected for debridement and implant retention. Antibiotic duration can be selected according to the time of diagnosis of IOM as it takes into account the biofilm formation and the state of fracture consolidation:

a) In patients with early infections (those that occur in the first 2 weeks after implantation of the osteosynthesis material) in whom early debridement is performed, it is possible to shorten the duration of antibiotic treatment to 8 weeks (versus 12 weeks);

b) In patients with delayed infections (those that occur between 3 and 10 weeks after implantation of the osteosynthesis material) in whom it is not possible to remove the implant due to instability/lack of fracture healing, it is possible, after surgical debridement, to shorten the duration of antibiotic treatment to 12 weeks (vs. maintaining antibiotic treatment until fracture healing or implant removal).

c) In patients with late infections (those that occur more than 10 weeks after implantation of the osteosynthesis material) healing is not possible until the implant is removed, but when removal is not possible, antibiotic therapy can be maintained until fracture healing or implant removal.

1.2 Primary Study Objective

To evaluate whether, after performing surgical debridement in patients with IOM due to a large bone fracture, a short antibiotic treatment is as effective as a longer treatment.

1.3 Secondary Study Objectives

- To evaluate the efficacy and safety of different antimicrobials used in IOM
- To evaluate the development of antimicrobial resistance during antibiotic treatment
- To evaluate the need for new surgeries during follow-up
- To provide information about the functional prognosis and quality of life of the patient according to each of the treatment strategies
- To evaluate the consumption of health resources with each type of strategy
- To evaluate the different reconstruction strategies (bone and soft tissue) carried out in order to recover lost functionality (degree of mobility and autonomy).

1.4 Primary endpoint

The primary endpoint is the variable "cure” composed by all of the following: a) clinical cure in the test of cure (TOC) (see definition below); b) radiological healing; c) definitive soft tissue coverage at TOC.

The definitions for all the outcomes are specified in the study protocol. The TOC will be performed 12 months after the end of antibiotic therapy prescribed to treat IOM following DAIR, except in patients with delayed infections randomized to the long-treatment regimen (until fracture union) who receive 6 months or more of antibiotic treatment, which will be evaluated 18 months after the start of the study (or randomization).

1.5 Secondary endpoints

a) **Clinical endpoints:** efficacy of each group of antibiotics, secondary infections, infection recurrence rate (relapses and reinfections), need for new surgeries (debridement, material removal, covering, amputation), reconstructive surgery strategies (bone and soft tissue), functional status (defined as restoration of limb function prior to fracture) and state of health.

b) **Microbiological endpoints:** Resistance development to antibiotic used during therapy and *C. difficile* infection during therapy and at 30-day.

c) **Adverse events (AE) and complications:** Adverse events and severity, including death (that is, all-cause mortality).

d) **Consumption of health resources:** Consumption of health resources will be evaluated for each type of strategy: days of antibiotic treatment, length of hospital stay, readmissions, number and type of surgeries performed.

1.6 Interim Analyses

Interim analysis will be conducted when 75 patients have been included to assess the frequency of the event rate and inclusion rate to adjust the intended size of the study population.

2. STUDY POPULATIONS

The populations for analysis will be:

2.1 Intent-to-treat (ITT) population (ITT): all appropriately-included and randomized patients.

2.2 Per protocol population (PP): all patients who received the complete antibiotic treatment regimen according to the inclusion group.

2.4 Clinically evaluable population (CEP): all patients with evaluation of success at the test of cure at 12 months after completion of treatment, or who have clinical failure before TOC.

3. ASSIGNMENT TO POPULATIONS AND PROTOCOL VIOLATIONS

To avoid a possible bias due to the open nature of the trial, the evaluation of the results will be double-check by an independent committee blinded (not part of the study as investigators) with respect to the assignment of treatment. This committee will be made up of 3 expert researchers who are not participating as researchers in this study and who will reach their conclusions by consensus after reviewing the consistency of outcome data as included in the CRF by local investigators, once these data has been verified by the monitoring team according to source data. All protocol violations occurring after randomisation will be collected. The final assignment of participants to the ITT, PP and CEP will be made after reviewing the protocol violations prior to database lock, with assessors blinded to the outcomes.

4. DATABASE AND MISSING DATA

4.1 Database archive and validation

The study database will be exported from the electronic CRF to SPSS software, and will be maintained at FISEVI files. The Spanish Clinical Research Network (SCReN) team will monitor the data for coherence, missing values and outliers, according to their standard monitoring procedures. All corrections will be performed by the study team together with the external monitoring team before database lock.

4.2 Missing Data

Missing data for key study variables, if any, will be presented and compared across study arms. Missing data for secondary exposure or variables will be considered as such and presented. No imputation of missing data is planned.

5. STATISTICAL ANALYSIS

5.1 Primary endpoint

The primary endpoint will be collected as a dichotomus variable (yes/no). The primary analysis will be the absolute difference in the proportion of patients reaching the composited primary outcome variable “cure” in the study arms (with one-sided 95% confidence interval) in the ITT population, with the long antibiotic treatment as the reference group. The specific reasons for not reaching overall success (failure, withdrawals, death, etc.) will be specified in both study arms.

A secondary analyses will include subgroups analysis for the primary outcome using the same procedure, and the comparison of the composite variable components (clinical cure, radiological healing and soft tissue coverage) in the PP and CEP, after excluding incorrectly assigned patients.

5.2 Secondary endpoints

The secondary endpoints will be collected as dichotomus variables (yes/no) or continuous variables. The ITT, PP and CEP approaches will be used for the analyses of secondary endpoints. The absolute difference with one-side 95% CI proportion will be used for dichotomus variables and absolute difference with one-side 95% CI mean/median (as appropriate) for continuous variables.

5.2.1 Clinical endpoints:

Efficacy of each group of antibiotics used, secondary infections, infection recurrence rate (relapses and reinfections), need for new surgeries (debridement, material removal, covering, amputation), reconstructive surgery strategies (bone and soft tissue), and functionality of the lower limbs will be reported as proportion. Risk ratios (RRs) and the absolute difference for achieving each endpoint (with 95% CIs), with the long antibiotic treatment arm as the reference group, will be calculated.

Functionality of the upper limbs (Quick-DASH score) and health outcomes (SF-12 or Barthel scores) will be reported as median. The median of each score will be compared between the two treatment groups using parametric or nonparametric tests as appropriate.

5.2.2 Microbiological endpoints: Resistance development to antibiotic used during therapy and *C. difficile* infection during therapy and at 30-day will be reported as proportion. Risk ratios (RRs) and the absolute difference for achieving each endpoint (with 95% CIs), with the long antibiotic treatment arm as the reference group, will be calculated.

5.2.3 Adverse events (AE) and complications: Adverse events and severity, including death (that is, all-cause mortality) will be reported as proportion. Risk ratios (RRs) and the absolute difference for achieving each endpoint (with 95% CIs), with the long antibiotic treatment arm as the reference group, will be calculated.

5.2.4 Consumption of health resources will be evaluated for each type of strategy: days of antibiotic treatment, length of hospital stay, readmissions, and number of surgeries performed will be reported as median. The median of each variable will be compared between the two treatment groups using parametric or nonparametric tests as appropriate.

5.3 Subgroup analyses

The primary and secondary endpoint will be analysed in the following subgroups:

- Type of infection: early and delayed.

- Type of fracture: open (Gustilo and Anderson classification) or closed.
- Type of osteosynthesis material: endomedullary nail or needle, plates or screws.
- According to the etiology of the infection: infections by different microorganisms, infections by multidrug-resistant microorganisms.
- According to type of patient: comorbidities, elderly, renal insufficiency, gender.

5.4 Multivariate analysis

A multivariate analysis will be performed in order to control the potential residual effect of variables other than duration of antibiotic therapy on the primary outcome by logistic regression. Potential confounders will be identified by bivariate analysis of their associations with the primary outcome, considering a *p* value <0.15, and will be entered into the multivariate model to check if the effect of the duration of therapy is significantly modified. Expected potential confounders include age, Charlson, delay in DAIR, type and location of fracture, type of osteosynthesis material, microorganism, and antibiotic group. The effect of study sites will be explored by classifying the sites as those with higher and lower cure rates, and including it as a potential confounder.

5.5 DOOR/RADAR analysis

In order to provide additional information of the different components of the primary endpoint, an analysis with desirability of outcome ranking (DOOR) and response adjusted for duration of antibiotic risk (RADAR) will be performed. First, patients will be classified according to the following ordinal system:

Category	Failure	No bone healing	No soft tissue coverage	Severe adverse event
1	No	No	No	No
2	No	No	No	Yes
3	No	No	Yes	Yes or no
4	No	yes	Yes or no	Yes or no
5	Yes	Yes or no	Yes or no	Yes or no

Second, in case of tie, the duration of total antibiotic duration will be considered; patients with a shorter duration will be given a better category. Finally, the probability of having a worse ranking among patients treated with a short antibiotic regimen than among patient with a long antibiotic regimen will be calculated; the upper bound of the 95% CI of the probability should be >50% to suggest non-inferiority of short antibiotic regimen.
