# Supplementary material for: Efficacy and safety of different antimicrobial DURATions for the treatment of Infections associated with Osteosynthesis Material implanted after long bone fractures (DURATIOM): Protocol for a randomized, pragmatic trial
Source: PLoS One. 2023 May 22;18(5):e0286094. doi: 10.1371/journal.pone.0286094 (PMC10202272; doi:10.1371/journal.pone.0286094)
Supplement: S4 File — This document contains the statistical analysis plan. (PDF) [file pone.0286094.s004.pdf]

CLINICAL TRIAL PROTOCOL

A MULTICENTER RANDOMIZED OPEN AND PRAGMATIC PHASE 3 CLINICAL STUDY TO EVALUATE THE EFFICACY AND SAFETY OF DIFFERENT DURATIONS OF ANTIMICROBIAL TREATMENT OF INFECTIONS ASSOCIATED WITH OSTEOSYNTHESIS MATERIAL AFTER LONG BONE FRACTURE (DURATIOM).

CODE: DURATIOM

EUDRACT No.: 2021-003914-38

VERSION: 2.1 of 04/Aug/2022

The information contained in this document is confidential and may not be disclosed to other people without the written authorization of the researchers, except for the use that may be made of it to obtain the informed consent of the people who are going to receive the investigational drug, as well as in communications to health authorities, clinical trial committees or those who are going to carry out the study.

1.- SUMMARY

1.1 Type of request

A Phase 3 clinical study to evaluate the efficacy and safety of short regimens versus long regimens of different authorized antimicrobials in infections associated with osteosynthesis material (IOM) implanted after long bone fractures and treated with surgical debridement without implant removal.

1.2 Identification of the promoter

Fundación Pública Andaluza para la Gestión de la Investigación en Salud de Sevilla (FISEVI).
Hospital Universitario Virgen del Rocío.
Laboratory building, 6th floor.
Avda. Manuel Siurot S/N
41013/Seville
Tel. 600 162 458/ 955013284 (Macarena site)
Fax: 955008016 (Macarena site)

1.3 Title of the clinical trial

A multicenter randomized open and pragmatic phase 3 clinical study to evaluate the efficacy and safety of different durations of antimicrobials for the treatment of infections associated with osteosynthesis material after long bone fractures (DURATIOM).

1.4 Protocol code

DURATIOM

1.5 Coordinating researcher

Dr. María Dolores del Toro López
Hospital Universitario Virgen Macarena
Clinical Unit of Infectious Diseases and Clinical Microbiology
Avda. Dr. Fedriani, 3
41009 Seville

1.6 Centers in which the trial is planned

Multicenter study involving Spanish hospitals listed in Annex II

1.7 Clinical Research Ethics Committee(s)

Reference committee: Provincial CEIm of Seville.

1.8 Responsible for monitoring

Clinical Research and Clinical Trials Support Unit, Clinical Research and Clinical Trials Unit, Hospital Universitario Virgen del Rocío-SCReN (Spanish Research Network).

1.9 Experimental treatment and control

The antimicrobial agent selected to treat IOM will be at the discretion of the physician responsible for the inclusion of the patient; the choice depends on the isolated microorganism and the susceptibility pattern, so any of the antibiotics commonly used in routine clinical practice may be used.

In all cases, the indications, doses, and instructions for administration used are those recommended in the technical sheet or by local and international scientific societies.

In all cases, it will be verified that the antimicrobial treatment is appropriate for the type of microorganism isolated and the type of patient's infection, and that the dose is correct based on the recommendations mentioned.

The duration of treatment will depend on the arm in which the patient is assigned.

Experimental arm; Short targeted (appropriate) antimicrobial therapy:

In early infection (<2 weeks from osteosynthesis material implantation): 8 weeks of treatment.
In delayed infection (2-10 weeks from osteosynthesis material implantation): 12 weeks of treatment.

Control arm; Long targeted (appropriate) antimicrobial therapy:

In early infection (<2 weeks from osteosynthesis material implantation): 12 weeks of treatment.
In delayed infection (2-10 weeks from osteosynthesis material implantation): Until fracture consolidation

1.10 Phase of the clinical trial

Phase 3; drugs in clinical practice, with unconfirmed duration.

1.11 Objectives

Main objective:
To evaluate whether, after surgical debridement in patients with infection of the osteosynthesis material (IOM) implanted after a long bone fracture, a short-term antimicrobial treatment is as effective as a prolonged treatment according to the technical data sheets authorized for each product.

Secondary objectives:

- To evaluate the efficacy and safety of different antimicrobials used in clinical practice and according to guidelines, in the IOM.
- To evaluate the development of antimicrobial resistance during antibiotic treatment.
- To evaluate the need for new surgeries during follow-up.
- To provide information on the functional prognosis and patients´ quality of life according to each of the therapeutic strategies.
- To evaluate the consumption of healthcare resources with each type of strategy.
- Evaluation of different reconstruction strategies (bone and soft tissue) performed in order to recover lost functionality (degree of mobility and autonomy).

1.12 Design

Pragmatic, multicenter, open-label, randomized, non-inferiority trial comparing different durations of antibiotic therapy in infections of osteosynthesis material (IOM) implanted after long bone fractures treated with debridement, antibiotic and implant retention (DAIR). The CONSORT (Consolidated Standards of Reporting Trials) guidelines for pragmatic designs will be used.

1.13 Study disease or disorder

Infections associated with osteosynthesis material (IOM) implanted after long bone fracture.

1.14 Primary endpoint

The primary outcome variable is a composite variable that includes:

a) clinical cure in the test of cure;

b) radiological healing;

c) definitive soft tissue coverage at the test of cure.

The test of cure will be performed 12 months after the end of antibiotic therapy.

1.15 Study population and total number of patients

The study population will be consisting of patients (over 14 years of age) with an infection of the osteosynthesis material implanted after a long bone fracture and who are treated by surgical debridement with implant retention plus targeted antimicrobial therapy. The sample size needed to achieve the objectives is estimated at 364 patients (182 per arm).

1.16 Duration of treatment

The duration of targeted antibiotic treatment will be 8 weeks for early infections and 12 weeks for delayed infections in the experimental arm (short course treatment); 12 weeks for early infections and until fracture consolidation in delayed infections in the control arm (long course treatment).

1.17 Schedule and expected completion date

The duration of the clinical trial is expected to be four years from the start of the recruitment period. A period of 3 months is included for submitting the documentation

to the Spanish Agency for Medicines and Health Products (AEMPS) and to the Ethics Committees, as well as initial training in the participating centres. The patient recruitment period will be 26 months and another 6 months are estimated for the analysis and subsequent dissemination of results.

2. -INDEX

1.- SUMMARY……………………………………………………………………...2
1.1 Type of request…………………………………………………………….. 2
1.2 Identification of the promoter………………………………………………2
1.3 Title of the clinical trial……………………………………………………...2
1.4 Protocol code……………………………………………………………..2
1.5 Coordinating researcher……………………………………………………2
1.6 Centers in which the trial is planned……………………………………...2
1.7 Clinical Research Ethics Committee(s)…………………………………..3
1.8 Responsible for monitoring………………………………………………...3
1.9 Experimental treatment and control……………………………………… 3
1.10 Phase of the clinical trial……………………………………………………3
1.11 Objectives…………………………………………………………………… 4
1.12 Design………………………………………………………………………4
1.13 Study disease or disorder…………………………………………………...5
1.14 Primary endpoint…………………………………………………………..5
1.15 Study population and total number of patients…………………………5
1.16 Duration of treatment……………………………………………………….. 5
1.17 Schedule and expected completion date………………………………5
2. - INDEX…………………………………………………………………..7
3.- GENERAL INFORMATION…………………………………………............10
3.1 Trial identification…………………………………………………………...10
3.2 Type of clinical trial………………………………………………………… 10
3.3 Data relating to the promoter……………………………………………10
3.4 Description of the products under study…...……………………………...11
3.5 Data of the study investigators……………………………………………13
3.6 Laboratories, Medical Department or Related Institutions……………….. 13
4.- BACKGROUND……………………………………………………………..14
5.- HYPOTHESIS AND OBJECTIVES OF THE TRIAL……………………………19
5.1 Hypothesis………………………………………………………………….. 19
5.2 Objectives…………………………………………………………………20
6.- TRIAL DESIGN………………………………………………………………20
6.1 Trial variables…………………………………………………………… 20

6.2 Design……………………………………………………………………………..22
6.3 Randomization procedure……………………………………………………23
6.4 Masking………………………………………………………………………………24
6.5 Trial treatments……………………………………………………………….. 24
6.6 Follow-up of patients………………………………………………………… 25
6.7 Criteria for termination or discontinuation of the study………………………… 25
6.8 Permitted Medications and Treatments…………………………………………. 26
6.9 Storage and dispensing of medication………………………………………... 26
6.10 End of trial…………………………………………………………………………26
7.- SELECTION CRITERIA……………………………………………………27
7.1 Inclusion criteria……………………………………………………………… 27
7.2 Exclusion criteria……………………………………………………………… 28
7.3 Withdrawal criteria…………………………………………………………… 29
7.4 Definitions……………………………………………………………………………30
8.- TREATMENT OF SUBJECTS…………………………………………………..32
8.1 Treatment duration and settings…………………………………………….... 33
8.2 Rescue medication……………………………………………………………… 33
8.3 Calendar of visits and evaluations…………………………………………….. 34
8.4 Procedures per visit………………………………………………………….. 35
9.- ASSESSMENT OF EFFICACY………………………………………………46
9.1 Primary efficacy variable…………………………………………………….. 46
9.2 Laboratory tests………………………………………………………………..47
9.3 Microbiological studies……………………………………………………..47
10.- SECURITY ASSESSMENT…………………………………………………. 48
10.1 Security evaluations………………………………………………………… 48
10.2 Laboratory tests……………………………………………………………48
10.3 Definitions…………………………………………………………………… 48
10.4 Reporting and collection of serious adverse events………………………….. 50
10.4.1 Exceptions to the collection of standard AE………………………………… 52
10.5 Reference security information…………………………………………..53
10.6 Expedited notification of SUAR…………………………………………… 53
10.7 Expedited notification of other relevant safety information…………………... 54
10.8 Notification to investigators………………………………………………… 54

10.9 Pregnany……………………………………………………………………...54
11.- STATISTICS……………………………………………………………………..55
11.1 Calculation of the sample size……………………………………………...55
11.2 Statistical analysis……………………………………………………………55
11.3 Intermediate analysis …………………………………………………………..56
11.4 Definitions of Study Analysis Populations………………………………………56
11.5 Independent Evaluation Committee……………………………………………. 56
12.- ETHICAL ASPECTS……………………………………………………………57
12.1 Informed consent…………………………………………………………………58
12.2 Data protection…………………………………………………………………58
12.3 Responsibilities of study participants……………………………………………59
12.4 Monitoring and auditing……………………………………………………….. 59
12.5 Premature termination or suspension of the study……………………………60
12.6 Study Documentation…………………………………………………………..60
13.- FINANCING AND INSURANCE………………………………………………… 60
13.1 Financing…………………………………………………………………………...60
13.2 Insurance……………………………………………………………………….. 61
14.- PUBLICATION POLICY…………………………………………………………..61
ANNEX I. BIBLIOGRAPHY……………………………………………………………63
ANNEX II. LIST OF PARTICIPATING CENTERS…………………………………..66
ANNEX III .Executive summary of management of prosthetic joint infections. Clinical practice guidelines by the Spanish Society of Infectious Diseases and Clinical Microbiology (SEIMC)……………………………………………………….68
ANNEX IV. QUICK DASH QUIZ……………………………………………………...75
ANNEX V. SF-12 QUESTIONNAIRE AND BARTHEL INDEX……………….... 76
ANNEX VI. CALCULATION OF qSOFA AND CALCULATION OF SOFA……...79
ANNEX VII. VISUAL ANALOGUE SCALE (VAS) OF PAIN................................81
ANNEX VIII. S NOTIFICATION FORM... 82
ANNEX IX. PREGNANCY NOTIFICATION FORM...85
ANNEX X. HELSINKI DECLARATION OF THE WORLD MEDICAL ASSOCIATION..89

3.- GENERAL INFORMATION

3.1 Clinical trial identification

Study code: DURATIOM
EudraCT: 2021-003914-38

3.2 Type of clinical trial

Pragmatic, multicenter, open-label, randomized, non-inferiority trial comparing different durations of antibiotic therapy in infections of osteosynthesis material (IOM) implanted after long bone fractures treated with debridement, antibiotic and implant retention (DAIR). The CONSORT (Consolidated Standards of Reporting Trials) guidelines for pragmatic designs will be used.

3.3 Data relating to the promoter

Fundación Pública Andaluza para la Gestión de la Investigación en Salud de Sevilla (FISEVI).

Hospital Universitario Virgen del Rocío

Laboratory building, 6th floor.

Avda. Manuel Siurot S/N. 41013 Seville.

Tel. 955013645 / 955013284 (Hospital Universitario Virgen Macarena)

Fax: 955008016 (Hospital Universitario Virgen Macarena)

3.3.1 Person authorized by the promoter, medical monitor

Dr. Clara M. Rosso Fernandez

Clinical Research Unit and support for clinical trials

Clinical Research and Clinical Trials Unit

Virgen del Rocío University Hospital-SCReN (Spanish Research Network).

Avda. Manuel Siurot s/n. 41013 Seville

Tel.:955313414. Fax: 954232992

3.3.2 Responsible for monitoring

Clinical Research Unit and support for clinical trials

Clinical Research and Clinical Trials Unit

Virgen del Rocío University Hospital-SCReN (Spanish Research Network).

Avda. Manuel Siurot S/N. 41013 Seville

Tel.:955313414

Fax: 955095338

3.4 Description of the products under study

Experimental Arm: Short Targeted (appropriate) antimicrobial therapy:

In early infection (<2 weeks from osteosynthesis material (OM) implantation): 8 weeks of treatment.

In delayed infection (2-10 weeks from OM implantation): 12 weeks of treatment.

Control arm: Targeted long (appropriate) antimicrobial therapy:

In early infection (<2 weeks from OM implantation): 12 weeks of treatment.

In delayed infection (2-10 weeks from OM implantation): until fracture consolidation.

The antimicrobial agent selected to treat IOM will be at the discretion of the physician responsible for the inclusion of the patient; the choice depends on the isolated microorganism and the susceptibility pattern, so any of the antibiotics commonly used in routine clinical practice may be used. These antibiotics include the following active ingredients for parenteral administration during the first week after surgery: ampicillin, amoxicillin/clavulanic acid, cefazolin, cefepime, ceftazidime, ceftriaxone, cloxacillin, daptomycin, meropenem, teicoplanin, vancomycin; and for sequential oral administration until the end of treatment: amoxicillin, amoxicillin/clavulanic acid, ciprofloxacin, clindamycin, co-trimoxazole, levofloxacin, linezolid, moxifloxacin, rifampin. Exceptionally, other parenteral or oral antibiotics marketed could be used, which are not described exhaustively because the list is considerably long. The

indications, doses, and instructions for administration used are those recommended in the technical sheet or by local and international scientific societies (see Annex III).

In all cases, it will be verified that the antimicrobial treatment is appropriate for the isolated microorganism and the patient's infection, and that the dose is correct based on the recommendations mentioned.

The duration of antibiotic treatment will depend on the arm to which the patient is assigned:

a) In the experimental arm, the duration of antibiotic treatment will be 8 weeks in the case of early infections (<2 weeks from OM implantation), and 12 weeks in the case of delayed infections (2-10 weeks from OM implantation), counting from the surgical debridement performed to treat the infection. The antibiotic administered after debridement will be empirical according to the protocols of each hospital, and intravenous. The duration of intravenous treatment will be carried out at the discretion of the investigator, taking into account the symptoms and signs of infection, but in the absence of sepsis or post-surgical complications, it should not exceed 7 days. Once the discontinuation of intravenous treatment is considered, oral treatment will be administered as mentioned in the previous paragraph.

b) In the control arm, the duration of antibiotic treatment will be 12 weeks in the case of early infections (<2 weeks from the implantation of the OM), and until fracture healing in the case of delayed infections (2-10 weeks from the implantation of the OM), counting from the surgical debridement performed to treat the infection. The antibiotic administered after debridement will be empirical according to the protocols of each hospital, and intravenous. The duration of intravenous treatment will be carried out at the discretion of the investigator, taking into account the symptoms and signs of infection, but in the absence of sepsis or post-surgical complications, it should not exceed 7 days. Once the discontinuation of intravenous treatment is considered, oral treatment will be administered as mentioned in the previous paragraph.

3.5 Data of the study investigators

See annex II.

3.6 Laboratories, Medical Department or Related Institutions

Infectious Diseases and Microbiology Clinic Unit.

Virgen Macarena University Hospital.

Avenida del Doctor Fedriani s/n

41009 Seville

Contact phone: 600162310

Fax: 955926552

Clinical Unit of Orthopedic Surgery and Traumatology.

Virgen Macarena University Hospital.

Avenida del Doctor Fedriani s/n

41009 Seville

Pharmacy Clinic Unit

Virgen Macarena University Hospital

Avenida Doctor Fedriani s/n

41009 Seville

Clinical Research Unit and support for clinical trials

Clinical Research and Clinical Trials Unit

Virgen del Rocío University Hospital-SCReN (Spanish Research Network).

Avda. Manuel Siurot S/N

41013 Seville

Phone: 955313414

Fax: 955095338

4.- BACKGROUND

Burden of infection associated with osteosynthesis material.

Orthopedic implants for the fixation of fractures or osteosynthesis material are used for the internal fixation of fractures, allowing their stability and consolidation. Internal fixation devices are only temporarily needed and can be removed after the bone fracture has healed. In contrast, the joint prosthesis replaces the original joint when it has been irreversibly damaged by trauma, osteoarthritis, or other inflammatory joint pathology, and thus has a permanent function.

Infections associated with osteosynthesis material (IOM) are among the most feared and challenging complications of trauma surgery and can lead to total function loss or limb amputation, when complete functional recovery is expected without the infection. But in addition to increased patient morbidity and mortality, it entails a significant economic burden. Recent studies show that the average cost per patient is six to seven times higher in infected patients than in uninfected ones. Length of stay is the most important driver for total healthcare cost [1,2]. A recent systematic review focused on late IOM found a disease recurrence of 6-9%, with a 3-5% amputation rate of the affected limb [3].

The risk of infection after internal fixation is between 1% and 30% [4]. This wide range is due to variability in patient-related factors, fracture type and location, open versus closed fracture, type of fixation, late debridement of open fractures, and perioperative factors (antibiotic prophylaxis, NNIS index, asepsis, surgical technique, perioperative wound care) [5].

A particularly vulnerable population is the elderly, in whom fractures of the lower limbs due to falls are frequent. A study performed in patients with staphylococcal orthopedic device-related infections found that at the end of follow-up, older patients had a significantly worse functional outcome and impaired physical quality of life, as well as more frequently higher rates of persistent infections, and multidrug resistance than those associated with infections in younger patients [6].

Furthermore, IOM involve excessive consumption of antimicrobials (see below), with inappropriate prescriptions and prolonged durations that favour superinfections by

multidrug-resistant microorganisms, in addition to drug related toxicities, especially diarrhea due to *Clostridioides difficile*. In an international multicenter study conducted in patients with prosthetic joint infections (PJIs) treated with suppressive antibiotic therapy, 104 adverse effects were found in 81/302 patients (26.8%) during the follow-up period; most of them gastrointestinal (16.9%) and skin (5.3%) [7].

Previous results in clinical research

This is a neglected area for clinical research in drug therapy. In fact, there is a lack of well-designed studies with a sufficient follow-up period investigating the best treatment of IOM. Therefore, the management of this infection is mainly based on extrapolations from PJI, tradition and personal experiences, which cause a substantial heterogeneity among institutions and countries.

The first and only randomized clinical trial in IOM treated with debridement, antibiotics and implant retention (DAIR) was published in 1998 by Zimmerli et al [8], evaluating the clinical and microbiological efficacy of levofloxacin plus rifampicin versus levofloxacin alone in early staphylococcal PJI and IOM after fractures. The duration of infection symptoms had to be less than 3 weeks and the implant had to be stable. Fourteen and 15 patients were included in each group, respectively, of which 10 and 8, respectively, were IOM. Cure was significantly higher in the combination therapy group. Hip PJI and IOM were treated for 3 months and knee PJI for 6 months. Despite the low number of patients included, this study laid the basis for the treatment of PJI and was also extended to IOM. Subsequent studies done in PJI have tried to show that short treatments are as effective as long treatments when DAIR is performed. A recent meta-analysis, including one clinical trial and 9 observational studies, concludes that when treating PJI patients following DAIR, an 8-week course of antibiotic treatment for total hip arthroplasty and a 75-day course for total knee arthroplasty may be a safe approach [9]. When the implant is removed, a prospective single-center, unblinded, randomized trial involving 38 two-stage prosthetic removal procedures and 55 orthopedic implant infections, had shown that there was no statistically significant difference in the rates of clinical or microbiological remission between patients treated 4 compared with 6 weeks of systemic antibiotic therapy [10].

However, to the best of our knowledge, there are no well-designed studies comparing the best antibiotic treatment regimen and duration in IOM when the implant is retained. Most of the studies published so far involve few patients, mix different orthopedic devices, and a wide variety of fractures. A retrospective case-control study conducted in patients with PJI and IOM after a fracture failed to demonstrate that a longer duration of antibiotic treatment is associated with a lower rate of reinfection one year after debridement (11). However, prolonged antibiotic regimens, usually 3 months or more, are used in IOM. In two papers published by the same group, the success rate when DAIR is performed under optimal conditions is high, with a duration of antibiotic therapy of about 12 weeks (12-13). This has been shown in a recent meta-analysis that included 276 patients with IOM and found a cure rate of 86% when DAIR is performed <3 weeks after material implantation, between 82-89% in infections of 3-10 weeks, and 67% in infections of >10 weeks. But the heterogeneity was high, with a long duration of antibiotic therapy, always above 12 weeks, regardless of the time of infection (14).

Finally, a Cochrane Database Systematic Review of antibiotics for treating chronic osteomyelitis in adults concluded that evidence to support recommendations was of low quality (15), and a later randomized clinical trial (16) found no difference in cure rate when comparing oral vs. intravenous therapy in osteoarticular infections. This trial included 1054 patients, 247 with implant retention (including prostheses), and compared 6 weeks of intravenous treatment with 6 weeks of oral treatment, although most patients received treatment beyond 6 weeks (median 78 days for intravenous treatment and 71 for oral treatment). There were no differences on serious adverse events (27.7% in the intravenous group and 26.2% in the oral group).

Rationale for the design of the randomized trial

As explained above, we found no clinical trials evaluating the optimal duration of antibiotic therapy in IOM when the implant is retained. Because there are antibiotics that have been shown to be effective for the treatment of implant-associated infections, mainly in PJI, these antibiotics could be used in these infections. Finding the shortest duration of antimicrobial treatment is a priority in infectious diseases, as a way to reduce exposure to antibiotics and helping to control antimicrobial resistance and avoiding adverse events and unnecessary costs. The non-inferiority approach is

justified because the use of shorter courses of treatment would benefit patients by reducing the selective pressure of broad-spectrum antibiotics commonly used in these infections. A superiority trial could be performed using a composite outcome variable that includes, for example, colonization and/or superinfection by multidrug-resistant bacteria or serious adverse events, but it would require a very large sample size that would make it unfeasible for this project. It is well known that classical trials may not adequately reflect clinical practice, since they are usually conducted in selected centers with highly experienced investigators and selected participants who do not really represent the majority of patients to whom the results would be extrapolated, so the benefits could be overestimated, and the harm underestimated. This led to the idea that more pragmatic trials demonstrating the real-world effectiveness of the intervention in broader patient groups, are required [17]. In addition, it can be particularly important in the evaluation of antibiotics, since the prognosis of the infection depends not only on the treatment itself, but also on the characteristics of the patient, the severity of the infection, the type of fracture, the microorganism, and the different aspects of surgical management (debridement, implant removal, soft tissue coverage...). Therefore, we propose a pragmatic trial. We used the PRECIS-2 tool to evaluate the level of pragmatism of our design [18] and followed the recommendations of extension of the CONSORT document for this type of trials [19]. To determine the duration of antibiotic treatment, we decided to classify IOM according to the age of the implant or the onset of infection symptoms, since it takes into account the biofilm formation and the state of fracture consolidation [4]. Briefly, this IOM classification includes: a) Early infection (<2 weeks): the bacteria may already have formed a biofilm which may still be in an "immature" phase, the bone does not show signs of osteomyelitis or osteolysis despite the presence of bacteria, and bone healing/consolidation is in the “soft callus or inflammatory stage”; b) Delayed infection (2-10 weeks): biofilms is mature and more resistant to antibiotic treatment, although normal bone healing takes up to 10 weeks ("hard callus stage" is situated between 3 and 16 weeks), the presence of bacteria weakens the callus formation. Bacterial invasion and inflammation of the bones ("osteomyelitis") often occurs; c) Late infection (> 10 weeks): mainly caused by low virulence microorganisms such as *S. epidermidis*, although bone consolidation may occur in some cases, severe inflammation and osteolysis with osteomyelitis lead to instability of the osteosynthesis. In contrast to PJI,

as we discussed above, in IOM the primary objective is not the complete eradication of the infection, but the healing and stabilisation of the fracture, as the material can be removed once the fracture has healed. Successful management of IOM includes fracture consolidation, restoration of the soft tissue envelope, return to function, prevention of residual chronic infection and eradication of infection. The surgical strategy will therefore depend on the onset of symptoms and the healing and stabilisation of the fracture, although the type of implant (plate, nail...), the location of the fracture (diaphyseal, articular), the condition of the skin and soft tissues, and the patient's situation play an important role. In early infections, consolidation can be achieved without removal the material if the osteosynthesis is stable. In these cases, a thorough surgical debridement should be performed, with sampling for microbiology, followed by empirical antibiotic therapy and then targeted at the isolated microorganisms. The duration of antibiotic treatment is unclear, and although 12 weeks is recommended based on a low-quality clinical trial (8), it will depend on early diagnosis and debridement, the severity of the infection, the patient's condition, and the skin and soft tissue status. Based on PJI studies, 8 weeks of antibiotic treatment seems to be sufficient time in case of debridement. In delayed infections, when removal of the material is not possible because the fracture is not healed, and provided the fracture is stable and debridement is performed to remove necrotic tissue, haematomas and decrease the bacterial load, 12 weeks of treatment may be sufficient. Late infections without removal the implant are likely to require suppressive treatment until the fracture has healed or until the material can be removed (5).

We decided to use a composite primary endpoint to include very relevant variables (clinical cure, radiological healing, definitive soft tissue coverage) which address overall recovery from infection, but we excluded the variable functional status because it may not always be related to infection.

In summary, most studies and recommendations on the management of orthopedic implant-associated infections have focused on PJI, and while they share similarities with IOM, there are major differences in terms of risk factors, diagnosis, treatment, and prevention. While the extrapolation of knowledge from PJI to the field of IOM has helped orthopedic surgeons and infectologists in their management, there are large gaps and unanswered questions that represent a new challenge for research in this

field. One of these challenges, and focusing on the field of infection treatment, is the search for the best surgical procedure according to the age of the implant and fracture healing, to determine the duration of antibiotic treatment and whether it should be maintained until fracture healing, and which is the most appropriate treatment in each of the scenarios. The identification of conditions in which the duration of the antimicrobial treatment can be shortened, and in case of prolonged treatments, which antimicrobial has the best safety profile and less impact on the induction of bacterial resistances is of special interest, due to the frequent appearance of toxicities and the increase of bacterial resistances and superinfections by multidrug-resistant bacteria.

5.- HYPOTHESIS AND OBJECTIVES OF THE CLINICALTRIAL

5.1 Working hypothesis

In infection associated with osteosynthesis material (IOM) of long bone fracture, a short antibiotic treatment is equally effective as a prolonged antibiotic treatment in patients selected for surgery with implant retention. Antibiotic duration can be selected according to the time of diagnosis of the infection:

a) In patients with early infections (<2 weeks from OM implantation) in whom early debridement is performed, it is possible to shorten the duration of antibiotic treatment to 8 weeks (versus 12 weeks);

b) In patients with delayed infections (2-10 weeks from OM implantation) in whom it is not possible to remove the implant due to instability/lack of fracture healing, it is possible, after surgical debridement, to shorten the duration of antibiotic treatment to 12 weeks (versus maintaining antibiotic treatment until fracture healing).

c) In patients with late infections (>10 weeks), healing is not possible until the implant is removed, but when removal is not possible, antibiotic treatment can be maintained until the fracture healing or implant removal.

5.2 Objectives

Main objective:

To evaluate whether, after performing surgical debridement in patients with IOM from a long bone fracture, a short antibiotic treatment is as effective as a prolonged treatment.

Secondary objectives:

a) To evaluate the efficacy and safety of different antimicrobials used in IOM.

b) To evaluate the development of antimicrobial resistance during antibiotic treatment.

c) To evaluate the need for new surgeries during follow-up.

d) To provide information on the functional prognosis and the quality of life of the patients according to each of the therapeutic strategies.

e) To evaluate the consumption of healthcare resources in each type of strategy

f) Evaluation of different surgical reconstruction strategies (bone and soft tissue) performed in order to recover lost functionality (degree of mobility and autonomy).

6.- TRIAL DESIGN

6.1 Trial variables

6.1.1 Primary endpoint.

The primary outcome variable is a composite variable that includes:

a) Clinical cure in the Test of Cure (TOC);

b) Radiological healing;

c) Definitive soft tissue coverage at the TOC.

The TOC will be performed 12 months after the end of antibiotic therapy.

Clinical cure is defined as:

- The absence of clinical signs and symptoms of infection (persistence of symptoms of infection, relapse of infection after a period without symptoms, or superinfection by a different microorganism) without antibiotic therapy and with CRP <10 mg/L (unless another cause justifies a highest CRP value)
- If the patient dies, when the death was not related to the infection
- No need for chronic suppressive antibiotic treatment to "control" the infection

Radiological healing is defined as the presence of radiological signs of fracture consolidation (plain radiography or CT) of the infected bone. Pseudarthrosis or absence of consolidation of a fractured bone is defined as the lack of complete union in the 9 months following osteosynthesis surgery or when it has not shown progression towards fracture callus formation in 3 consecutive months on serial radiographs [22]. Given the subjectivity that can sometimes occur in the assessment of the healing criteria, a blinded investigator will evaluate the concordance of the clinical and radiological healing criteria performed by the local investigator.

The main variable will also be evaluated by subgroups:

- By type of infection: early and delayed.
- By type of fracture: open (Gustilo and Anderson classification) or closed.
- Type of osteosynthesis material: endomedullary nail, plates, screws...
- According to the aetiology of the infection: infections by different microorganisms, infections by multidrug-resistant microorganisms.
- According to the type of patient: comorbidities, elderly people, renal insufficiency, etc.

6.1.2 Secondary outcomes

- Appearance of adverse events (frequency and severity).
- Development of antibiotic resistance during treatment.
- Development of secondary infections.
- Recurrence rate (relapses and reinfections)
- Need for new surgeries (debridement, material removal, covering, amputation).
- Functional status (defined as the restauration of limb function prior to fracture) and quality of life.
- Consumption of healthcare resources
- Evaluation of different surgical reconstruction strategies (bone and soft tissue) performed in order to recover lost functionality (degree of mobility and autonomy).

The assessment of the patient's functional status is complex given the number of fractures and bones involved. To facilitate this, the following has been chosen to be performed at TOC:

- <u>Functional status</u>: Normal or reduced compared to the situation prior to fracture. Lower limbs mobility is classified as: unassisted walk; with 1 cane; with 2 canes; with walker; "home alone ambulation"; no ambulation. For the upper limbs mobility, we will use the Quick-DASH questionnaire (See Annex IV).
- To evaluate <u>health outcomes</u> from the patient's perspective, we will use the SF-12 questionnaire and for patients over 70 years of age, the Barthel scale will be used, which is more appropriate for this age group. (See annex V).

6.2 Design

Pragmatic, multicenter, non-inferiority, open-label, randomized trial comparing different durations of antibiotic therapy in osteosynthesis material infections (IOM) after long bone fractures treated with debridement and material retention. The CONSORT

(Consolidated Standards of Reporting Trials) guidelines for pragmatic designs will be used.

6.3 Randomization procedure

Candidate patients will be detected by daily review of admissions to the Traumatology Units, both from the Emergency Department and from outpatient clinics, as well as in the daily wards rounds of the Traumatology wards.

The screening will involve the Traumatology team, the nursing team and the Infectious Diseases/Internal Medicine team. Patients who meet all the inclusion criteria, but some exclusion criteria will be considered "screening failures", and the reason for exclusion will be recorded in a specific section of the Case Report Form (CRF).

Patients who sign the informed consent form will be randomized centrally using an automated online system. The randomization list will be obtained using Epidat 4.0 software. Assignment to the experimental/control arm will be generated automatically after online randomization in a system included in the same electronic CRF (eCRF) designed for the study. The proportion of experimental (short treatment) / control (long treatment) arms will be 1:1 and will be stratified according to whether they are early or delayed infections, and by hospital. Randomization by hospital is justified to avoid the center effect due to the variability in clinical practice among centers.

Patients must be randomized no later than 72 h after knowing the microorganism causing the IOM in samples obtained in DAIR, therefore in approximately 7-10 days following debridement. Antibiotic administered after surgical debridement will be carried out empirically according to the protocols of each hospital. The duration of intravenous treatment will be at the discretion of the investigator, but it is advised that in the absence of sepsis or post-surgical complications it should not exceed 7 days.

Antibiotics given before surgery will be allowed in both groups, but will not be governed by the trial protocol. All antibiotics administered in the 2 weeks prior to surgery and their duration will be recorded.

Once intravenous treatment is considered to be discontinued, the antibiotic treatment administered orally will be agreed upon by the Traumatology-Infectious Diseases/Internal Medicine-Microbiology team. Given the absence of controlled studies carried out specifically in IOM, it will be performed according to the prosthetic joint infection guidelines [20]. Adjunctive oral antibiotics such as rifampin are allowed at the discretion of the infectious disease or microbiology specialist, reflecting usual practice. In the switch to the oral route, antibiotics with good bioavailability should be used [20], and if no options are available, outpatient parenteral antimicrobial therapy can be used. In case of intercurrent infection unrelated to IOM, up to 7 days of concomitant antibiotic treatment is allowed.

The fact that antibiotic treatment is chosen by the treating physician introduces a challenge for the analysis. However, there is already experience with this approach, for example in oral switch in osteomyelitis or endocarditis (OVIVA and POET trials) [16, 21].

6.4 Masking

There are no masking techniques since it is an open study. Therefore, no blind rupture procedure is applicable either.

6.5 Trial treatments

The antimicrobial agent selected to treat the IOM will be at the discretion of the physician responsible for the inclusion of the patient, provided that it is appropriate for the isolated microorganism, so any of the antibiotics commonly used in routine clinical practice may be used to this end. In any case, the indications, doses and instructions for administration used will be those recommended in the technical sheet or by the local and international scientific societies.

In all cases, it will be verified that the antimicrobial treatment is appropriate for the isolated microorganism and the patient's infection, and that the dose is correct based on the recommendations mentioned.

The duration of treatment will depend on the arm to which the patient is assigned:

Experimental Arm: Short Targeted (appropriate) Antimicrobial Treatment:

- In early infection (<2 weeks from OM implantation): 8 weeks of treatment.
- In delayed infection (2-10 weeks from OM implantation): 12 weeks of treatment.

Control arm: Targeted long (appropriate) antimicrobial treatment:

- In early infection (<2 weeks from OM implantation): 12 weeks of treatment.
- In delayed infection (2-10 weeks from OM implantation): until fracture consolidation.

6.6 Follow-up of patients

All patients will be evaluated: the day of recruitment (day 0, visit 1), the day of switching from intravenous to oral antibiotic treatment (days 7±3, visit 2) or at discharge if the patient goes home with oral antibiotic, at one month (day 28 ± 7, visit 3) for safety of treatment, at 8 weeks (±7 days) (visit 4) for the withdrawal of antibiotic treatment in the group of early infections randomized to the short regimen, at 12 weeks (±7 days) (visit 5) for the withdrawal of antibiotic treatment in the arm of early infections randomized to the long regimen and in the arm of delayed infections with the short regimen, at 6 months (±2 weeks) (visit 6), and at 12 months (±1 month) (visit 7), for evaluation only of patients with delayed infections and a long course of antibiotics who are still receiving treatment, and at one year after completion of antibiotic treatment (±1 month) (test of cure, visit 8). If any event occurs between visits (relapses, new hospitalization or surgery, adverse effects, antibiotic changes), additional visits will be performed and collected.

6.7 Criteria ending the study or discontinuing treatment

Early discontinuation of the clinical trial may occur due to a decision by the regulatory authorities, due to a change in the opinion of the Clinical Research Ethics Committee, due to problems related to the safety of the drug or due to inefficacy data.

The investigator, as well as the sponsor, reserve the right to discontinue the study at any time for reasonable medical and/or administrative reasons.

6.8 Permitted Medications and Treatments

The use of any antibiotics used in routine clinical practice for the treatment of IOM is allowed, with the usual doses and instructions for administration described in the technical sheet or that are recommended by local and international scientific societies in each case.

No restrictions are established for the use of other drugs, however, interactions with drugs, contraindications, and warnings of the technical sheets of the investigational antibiotic used in the trial must be taken into account. The antibiotic therapy administered in the 15 days before surgery, the complementary antibiotic to targeted therapy (such as rifampin), and antibiotics required for an infection other than IOM, will be collected as concomitant medication.

6.9 Storage and dispensing of medication

Medicines authorized by the National Health System will be used, through the usual prescription and dispensing routes of each center.

6.10 End of trial

The day of the final visit of the last patient included in the study will be considered the end of the trial.

7.- SELECTION CRITERIA

Patients > 14 years old, with an infection of the osteosynthesis material (IOM) implanted after a long bone fracture diagnosed during the study inclusion period, and who are treated by surgical debridement, antibiotics and implant retention (DAIR).

IOM is considered when at least one microbiological and one clinical criterion are met:

- Microbiological criterion: Isolation of the same microorganism (same species and antibiogram) in ≥2 tissue samples taken intraoperatively (≥1 in the presence of virulent microorganisms such as *Staphylococcus aureus*) or from the combination of cultures taken preoperatively by deep aspiration plus intraoperative cultures
- Clinical criteria:
    - Presence of pus around the implant.
    - Presence of a fistulous tract that communicates with the osteosynthesis material
    - Presence of histopathological signs of acute inflammation in the peri-implant tissues taken at the time of debridement.

7.1 Inclusion criteria

1. Age greater than or equal to 14 years.
2. Stabilized fracture, even if it is not consolidated.
3. Controlled infection (no signs or symptoms of sepsis).
4. Early or delayed infection.
5. Availability of active antibiotics against the isolated microorganism.

6. Absence of bone exposure. Patients who initially had bone exposure, but during surgical debridement a bone coverage was performed by any method (skin approximation, grafting, vacuum therapy), can be included in the criteria.

7. Patients who have signed the informed consent, in the case of minors, signature of the legal guardians and assent of the minor.

8. If there is a possibility of pregnancy (in women of childbearing age (See definition in section 7.4) or paternity, accept the use of a highly effective birth control method recommended by the Clinical Trial Facilitation Group (CTFG):https://legemiddelverket.no/Documents/Godkjenning/Klinisk%20utpr%C3%B8ving/2014_09_HMA_CTFG_Contraception_guidance%20Version%201.1.pdf) during the treatment phase of the trial.

7.2 Exclusion criteria

1. Late infections
2. IOM in a fracture of a bone other than long.
3. Infections of the revision osteosynthesis material or occurring after previous surgeries.
4. Patients unlikely to complete follow-up for at least 1 year after completion of antibiotic treatment.
5. Pregnant or breastfeeding women.
6. Patients in whom there may be drug interactions or contraindications described in the technical data sheets of the investigational drugs used in this trial.
7. Infections caused by mycobacteria, fungi, and parasites (since these infections are treated with different drugs and different durations).

8. Patients in whom the debridement involves replacement of all the material in the same surgical time (since these patients require a duration of antibiotic treatment of less than 8 weeks in all cases).

7.3 Withdrawal criteria

In accordance with the Declaration of Helsinki (Annex V), patients have the right to withdraw from the study at any time and for any reason and may do so personally or through their representative.

7.3.1 Withdrawal by efficacy criteria

When the experimental group has a lower-than-expected cure rate at the planned interim analysis.

7.3.2 Withdrawal due to safety criteria

When any adverse event occurs that, in the clinician's opinion, requires withdrawal from the study. When in the control arm a higher rate of adverse effects, superinfections and multidrug resistance is observed with the same cure as the experimental arm in the planned interim analysis.

7.3.3 Withdrawal due to non-compliance or violation of the rules contained in the protocol

The participant may be withdrawn from the study at the discretion of the investigator when he/she ceases to comply with the rules of the trial or when there is a loss of follow-up.

7.3.4 Follow-up of patients withdrawn early

In the event of early withdrawal of a patient from the trial, the researcher will provide the main reason for the suspension and as indicated by the GCP regulations, the procedures will be followed according to the usual treatment protocols for their pathology at the discretion of the responsible clinician.

7.4 Definitions

Early infection (<2 weeks from the implantation of the osteosynthesis material): those diagnosed in the first 2 weeks after the osteosynthesis material implantation surgery.

Delayed infection (2-10 weeks from the implantation of the osteosynthesis material): those diagnosed between the 2nd and 10th week after the osteosynthesis material implantation surgery.

Late infection: those diagnosed after the 10th week of the osteosynthesis material implantation surgery.

Clinical cure is defined as:

- Absence of clinical signs and symptoms of infection (persistence of symptoms of infection, relapse of infection after a symptom-free period, or superinfection by a different organism) without antibiotic therapy and with CRP <10 mg/L (unless another cause justify a higher CRP value)
- If the patient dies, when the death was not related to the infection
- No need for chronic suppressive antimicrobial therapy to "control" the infection
- In cases in which the osteosynthesis material is removed once the fracture has consolidated, when the intraoperative cultures and the sonicated culture of the implant are negative

Radiological consolidation is defined as the presence of radiological signs of fracture consolidation (plain radiography or CT) of the infected bone. Pseudarthrosis or lack of consolidation of a fractured bone is defined as the absence of complete fracture union in the 9 months after osteosynthesis surgery or when no progression towards the formation of fracture callus has been shown in 3 consecutive months in radiographs series [22].

Persistence of infection: no disappearance of clinical signs and symptoms of IOM, produced by the same microorganism that produced the initial infection.

Relapse of infection: reappearance of signs or symptoms of IOM after a period without symptoms, by the same microorganism that produced the original infection.

Superinfection: re-infection produced with a different microorganism than the one that caused the initial infection.

Clinical failure: When any of the following occurs:

- Absence of clinical and microbiological cure of the infection.
- Absence of radiological consolidation
- Exposure of bone and/or osteosynthesis material
- Infection-related death.

Fertile age: According to the World Health Organization (WHO), the reproductive stage of women is defined as between 15 and 49 years of age. In this delimitation there are exceptions such as early pregnancies or pregnancies at later ages because fertility varies according to each woman. But the usual thing is that from the age of 14, most women have begun to ovulate, so they would begin to be fertile, and after the age of 44 the biological possibilities of being able to be mothers naturally are very low.

8.- SUBJECTS TREATMENT

Patients will be classified according to the age of the implant, that is, according to the time elapsed from the implantation of the osteosynthesis material until the time of diagnosis of the infection:

A. Early infections: those diagnosed in the first 2 weeks after the surgery of the osteosynthesis material implantation.

B. Delayed infections: those diagnosed between the 2nd and 10th week after the surgery of the osteosynthesis material implantation.

C. Late infections: those diagnosed from the 10th week after the surgery of the osteosynthesis material implantation.

Patients with early or delayed infections undergoing DAIR will be randomized to the short treatment arm (8 weeks in early infections or 12 weeks in delayed infections) or the long treatment arm (12 weeks in early infections or antibiotics until fracture healing in delayed infections). Patients with late infections (>10 weeks) are excluded and therefore will not be randomised. Screening failure will be considered.

Patients in all groups will be followed for at least 12 months after antibiotic withdrawal.

Early infection (< 2 weeks) or

Delayed infection (2-10 weeks)

RANDOMIZATION 1:1

**Experimental arm**	**Control arm**
Short antibiotic treatment	*Long antibiotic treatment*
8 weeks (early infection)	12 weeks (early infection)
12 weeks (delayed infection)	Until fracture healing (delayed infection)

8.1 Treatment duration and settings

All patients will be evaluated: the day of recruitment (day 0, visit 1), the day of switching from intravenous to oral antibiotic treatment (days 7±3, visit 2) or at discharge if the patient goes home with oral antibiotic, at one month (day 28 ± 7, visit 3) for safety of treatment, at 8 weeks (±7 days) (visit 4) for the withdrawal of antibiotic treatment in the group of early infections randomized to the short regimen, at 12 weeks (±7 days) (visit 5) for the withdrawal of antibiotic treatment in the group of early infections randomized to the long regimen and in the group of delayed infections with the short regimen, at 6 months (±2 weeks) (visit 6), and at 12 months (±1 month) (visit 7), for evaluation only of patients with delayed infections and a long course of antibiotics who are still receiving treatment, and at one year after completion of antibiotic treatment (±1 month) (test of cure, visit 8). If any event occurs between visits (relapses, new hospitalization or surgery, adverse effects, antibiotic changes), additional visits will be performed and collected.

8.2 Rescue medication

Rescue medication is not foreseen. Since the antibiotics to be used are those of standard practice, if the antibiotic initially administered is not effective or an adverse effect occurs, it can be changed according to clinical guidelines or standard practice, without this being considered a failure or violation of the protocol. The alternative may be intravenous treatment on an outpatient or inpatient regimen. If there is no alternative and the antibiotic treatment must be withdrawn, the patient will be dropped from the study, but will continue to be followed according to the protocol and will be included in the intention-to-treat analysis. Changes in treatment, the reason, and adverse reactions will be recorded in the CRF.

8.3 Calendar of visits and evaluations

Procedures	Screening (up to 72 hours after knowing the IOM aetiology)	Visit 1 Day 0	Visit 2 Day 7 (+/-3 days)	Visit 3 Day 28 (+/-7 days)	Visit 4 8 weeks (+/-7 days)	Visit 5 12 weeks (+/-7 days)	Visit 6 6 months (+/- 2 weeks)	Unscheduled visit	Visit 7 12 months (+/- 1 month)	Visit 8, after12 months of finishing antibiotic treatment[4]
**Inclusion/exclusion criteria**	X									
**Informed consent**	X									
**Randomization**		X								
**Medical history / anamnesis**		X								
**qSOFA[2] calculation and temperature measurement**		X						X		
**Surgical wound exploration**<br><br>**Pain scale (from 1 to 10)**		X	X	X	X	X	X	X	X	X
**Fractured bone X-ray**		X		X	X	X	X	X	X	X
**Functional status assessment[3]**							x	X	X	X
**SF-12 questionnaire or Barthel scale (if >70 years)**		X					X	X	X	X
**Hematology/ biochemistry**		X	X	X	X	X	X	X	X	X
**Cultures**		x	X	X	X	X	X	X	X	X
**New surgery**			X	X	X	X	X	X	X	X
**Dispensing and administration of medication.**		X	X	X	X	X	X	X	X	X
**Concomitant medication**			X	X	X	X	X	X	X	X
**Adverse effects**			X	X	X	X	X	X	X	X

[1]Patients should be randomized no later than 72 h after knowing the microorganism causing the IOM, preferably from samples obtained at DAIR.

[2]qSOFA: Assess mental status, respiratory rate and blood pressure. If qSOFA ≥2, calculate SOFA.

[3]Functional status: Normal or reduced compared to the situation prior to the fracture. Lower extremity mobility is classified as: walks unassisted; with 1 cane; with 2 canes; with walker; "wandering home alone"; no wandering). For the mobility of the upper limbs, the Quick-DASH questionnaire will be used (See Annex IV).

[4]If patients with delayed infections randomized to the long treatment regimen (until fracture union) had 6 months or more of antibiotic treatment, they will be evaluated 18 months after the start of the treatment (or from the date of first surgical debridement after diagnosis of infection).

8.4 Procedures to perform per visit

The follow-up of the patients in the present study consists of 8 scheduled visits. In this protocol, the visits appear in chronological order and the day or interval of days in which they must be carried out appears in parentheses. The visits will preferably be carried out face-to-face. In the case of elderly patients or those unable to attend visits, if there are no incidents in the surgical wound or adverse effects, it can be done by telephone. Regardless of the type of visit, patients will be instructed to keep the empty antibiotic cartons or tablets in order to check the batch and expiration date. If the patient develops symptoms compatible with infection once the treatment has finished (visit 4 or 5 depending on the branch) and before visit 8 (12 months after the end of the treatment), an UNSCHEDULED VISIT will be carried out in less than 48-72 hours. To do this, the researcher must inform the patient about the symptoms of recurrence of infection and must provide a contact telephone number and/or an address to contact in that case.

Even if the trial is suspended or the patient leaves it, follow-up should continue to assess the test of cure 12 months after completion of antimicrobial treatment.

8.4.1 Screening visit.

- Identification of cases. Evaluation of clinical and microbiological criteria for infection of osteosynthesis material.
- Evaluation of the inclusion/exclusion criteria.
- Signing of the informed consent.

8.4.2 Selection Visit (Visit 1) (Day 0).

In the selection visit or visit 1, certain actions must be carried out as a step prior to inclusion, as well as the collection of data prior to inclusion. The procedures that will be carried out during this visit are detailed below:

- Randomization. It should not be performed later than 72 h after knowing the aetiology of IOM, preferably after obtaining the samples in the surgical debridement performed to treat the infection, that is, within 7-10 days of surgical debridement to treat the infection.

- Randomization will be performed by type of infection (early or delayed) and by center.

- Demographic data: age, sex, origin (address, social health residence).

- Clinical history/anamnesis including: comorbidities, allergies, Charlson and ASA index, type of fracture and affected bone (OTA classification and Gustilo and Anderson classification in open fractures), day of the fracture, procedures prior to the OM implantation (debridement of open wounds, stabilization with orthosis or external fixation), date of OM implantation and type of material, day of onset of symptoms of infection, signs and symptoms of infection, date of infection diagnosis. Surgical treatment received to treat the infection (debridement).

- Calculation of qSOFA (Quick SOFA Score for Sepsis identification) and SOFA if necessary, measurement of temperature and pain scale. See annex VI.

- Examination of the surgical wound: local inflammatory signs (edema, redness, cellulitis, ecchymosis), fistula (yes/no), wound drainage (amount: mild, moderate, significant; appearance: serous, hematic, purulent), dehiscence of suture (yes/no), exposure of osteosynthesis material or bone, state of the graft or skin coverage if performed.

- X-ray of the fractured bone (closest to debridement surgery).

- Completion of the SF-12 health questionnaire, or Barthel in people over 70 years of age.
- Collect blood count, biochemical data (with transaminases, bilirubin, and CRP). Pregnancy test in women of childbearing age
- Medication administration (day 1 of treatment). The empirical treatment received for IOM will be recorded, that is, the antibiotic treatment from the time the debridement is performed until the results of the cultures are known. If, after knowing the results of the cultures, it is decided to continue with the same antibiotic, the end date will be the date on which it is definitively withdrawn (to start, for example, sequential oral treatment). If the microorganism is known at the time of debridement, it will be considered from the beginning as directed treatment.
- Concomitant medication review.

8.4.3 Visit 2. Change from intravenous to oral treatment (Day 7 +/-3).

- From the 5th day of targeted intravenous treatment, an alternative oral route is allowed to continue until the regimen is completed, depending on the type of infection and the experimental or control arm. The duration of intravenous treatment will be carried out at the discretion of the investigator, but it is recommended in the absence of sepsis or post-surgical complications not to exceed 7 days.
- The oral antibiotic will be chosen according to clinical guidelines and usual practice. The choice will be based on relevant clinical variables: antimicrobial susceptibility, interactions, comorbidities, allergies, previous infections, or local epidemiology. Up to 7 days of treatment for an intercurrent infection unrelated to IOM is allowed without termination or deviation from the protocol. Adjuvant oral therapy, such as rifampin, reflecting current practice, is permitted.
- Clinical symptoms in relation to the patient clinical progress.

- Pain scale.
- Examination of the surgical wound: local inflammatory signs (edema, redness, cellulitis, ecchymosis), fistula (yes/no), wound drainage (amount: mild, moderate, significant; appearance: serous, hematic, purulent), dehiscence of suture (yes/no), exposure of osteosynthesis material or bone, state of the graft or skin coverage if performed.
- Blood extraction (complete blood count and biochemistry with transaminases, bilirubin, and CRP)
- Control of dispensing and administration of medication. Reasons for ending antibiotic treatment.
- Concomitant medication review.
- Assessment of adverse events.
- Need for new surgery and type.
- New cultures if done.

8.4.4. Visit 3 (Day 28 +/- 7).

- Clinical symptoms in relation to the patient clinical progress.
- Pain scale.
- Examination of the surgical wound: local inflammatory signs (edema, redness, cellulitis, ecchymosis), fistula (yes/no), wound drainage (amount: mild, moderate, significant; appearance: serous, hematic, purulent), dehiscence of suture (yes/no), exposure of osteosynthesis material or bone, state of the graft or skin coverage if performed.
- Radiography of the fractured bone: stability of the osteosynthesis, consolidation of the fracture.

- Blood extraction (complete blood count and biochemistry with transaminases, bilirubin, and CRP)
- Control of dispensing and administration of medication. Reasons for ending antibiotic treatment.
- Concomitant medication review.
- Assessment of adverse events.
- Need for new surgery and type.
- New cultures if done.

8.4.5 Visit 4 (8 weeks, +/- 7 days from recruitment). Withdrawal of antibiotics in the group of early infections in the short treatment arm.

- Clinical symptoms in relation to the patient clinical progress.
- Pain scale.
- Examination of the surgical wound: local inflammatory signs (edema, redness, cellulitis, ecchymosis), fistula (yes/no), wound drainage (amount: mild, moderate, significant; appearance: serous, hematic, purulent), dehiscence of suture (yes/no), exposure of osteosynthesis material or bone, state of the graft or skin coverage if performed.
- Radiography of the fractured bone: stability of the osteosynthesis, consolidation of the fracture.
- Blood extraction (complete blood count and biochemistry with transaminases, bilirubin, and CRP)
- Control of dispensing and administration of medication. Reasons for ending antibiotic treatment.
- Concomitant medication review.

- Assessment of adverse events.
- Need for new surgery and type.
- New cultures if done.

8.4.6 Visit 5 (12 weeks, +/- 7 days from recruitment). Withdrawal of antibiotics from the arm of early infections with long treatment and the arm of delayed infections with short treatment.

- Clinical symptoms in relation to the patient clinical progress.
- Pain scale.
- Examination of the surgical wound: local inflammatory signs (edema, redness, cellulitis, ecchymosis), fistula (yes/no), wound drainage (amount: mild, moderate, significant; appearance: serous, hematic, purulent), dehiscence of suture (yes/no), exposure of osteosynthesis material or bone, state of the graft or skin coverage if performed.
- Radiography of the fractured bone: stability of the osteosynthesis, consolidation of the fracture.
- Blood extraction (complete blood count and biochemistry with transaminases, bilirubin, and CRP)
- Control of dispensing and administration of medication. Reasons for ending antibiotic treatment.
- Concomitant medication review.
- Assessment of adverse events.
- Need for new surgery and type.
- New cultures if done.

8.4.7 Visit 6 (6 months, +/- 2 weeks from recruitment). In this visit, the patients who finished the antibiotic treatment (group of early infections, and group of delayed infections with a short regimen) and the group of delayed infections with a long regimen who may still be receiving antibiotics because the fracture has not yet consolidated will be evaluated. In this last group, the research team and the responsible medical team will assess whether additional surgical treatment is appropriate.

It will be collected:

- Clinical symptoms in relation to the patient clinical progress.
- Pain scale.
- Examination of the surgical wound: local inflammatory signs (edema, redness, cellulitis, ecchymosis), fistula (yes/no), wound drainage (amount: mild, moderate, significant; appearance: serous, hematic, purulent), dehiscence of suture (yes/no), exposure of osteosynthesis material or bone, state of the graft or skin coverage if performed.
- Radiography of the fractured bone: stability of the osteosynthesis, consolidation of the fracture.
- Completion of the SF-12 health questionnaire, or Barthel in people over 70 years of age.
- Completion of the Quick-DASH questionnaire in case of upper limb fracture or evaluation of lower limb mobility in case of lower limb bone fractures.
- Blood extraction (complete blood count and biochemistry with transaminases, bilirubin, and CRP)
- Control of dispensing and administration of medication. Reasons for ending antibiotic treatment.
- Concomitant medication review.

- Assessment of adverse events.
- Need for new surgery and type.
- New cultures if done.

8.4.8 Unscheduled visit

- If the patient experiences worsening of symptoms/signs of infection during antibiotic treatment or develops symptoms consistent with infection after completion of treatment (Visit 4 or 5 depending on arm) and before Visit 7 (12 months after completion of the same) an UNSCHEDULED VISIT must be carried out in a period of less than 48-72 hours. To do this, the researcher must inform the patient about the symptoms of recurrence of infection and must provide a contact telephone number and/or an address to contact in that case.
- Even if the trial had to be discontinued, patient follow-up should continue to assess the cure variable 12 months after completion.
- In this visit will be collected:
  - Clinical symptoms in relation to the patient clinical progress.
  - Calculation of qSOFA and temperature measurement.
  - Pain scale.
  - Examination of the surgical wound: local inflammatory signs (edema, redness, cellulitis, ecchymosis), fistula (yes/no), wound drainage (amount: mild, moderate, significant; appearance: serous, hematic, purulent), dehiscence of suture (yes/no), exposure of osteosynthesis material or bone, state of the graft or skin coverage if performed.
  - Radiography of the fractured bone: stability of the osteosynthesis, consolidation of the fracture.

- Any other imaging test may be requested if the responsible physician considers it necessary
- Completion of the SF-12 health questionnaire, or Barthel in people over 70 years of age.
- Completion of the Quick-DASH questionnaire in the case of an upper limb fracture or evaluation of the mobility of the lower limbs in the case of lower limb bone fractures if the visit occurs between 6 and 12 months.
- Blood extraction (complete blood count and biochemistry with transaminases, bilirubin, and CRP)
- Control of dispensing and administration of medication. Reasons for ending antibiotic treatment.
- Concomitant medication review.
- Assessment of adverse events.
- Need for new surgery and type.
- New cultures if done.

8.4.9 Visit 7 (12 months, +/- 1 month from recruitment).

- This visit will only be carried out in the group of patients with delayed infections with the long treatment regimen (until fracture consolidation).
- Clinical symptoms in relation to the patient clinical progress.
- Pain scale.
- Examination of the surgical wound: local inflammatory signs (edema, redness, cellulitis, ecchymosis), fistula (yes/no), wound drainage (amount: mild, moderate, significant; appearance: serous, hematic, purulent), dehiscence of

suture (yes/no), exposure of osteosynthesis material or bone, state of the graft or skin coverage if performed.

- Radiography of the fractured bone: stability of the osteosynthesis, consolidation of the fracture.
- Completion of the SF-12 health questionnaire, or Barthel in people over 70 years of age.
- Completion of the Quick-DASH questionnaire in case of upper limb fracture or evaluation of lower limb mobility in case of lower limb bone fractures.
- Blood extraction (complete blood count and biochemistry with transaminases, bilirubin, and CRP)
- Control of dispensing and administration of medication. Reasons for ending antibiotic treatment.
- Concomitant medication review.
- Assessment of adverse events.
- Need for new surgery and type.
- New cultures if done.

8.4.10 Visit 8 (12 months, +/- 1 month from withdrawal of antibiotic treatment).

Final evaluation visit at 12 months after completing the antibiotic treatment prescribed after randomization. Patients with delayed infections randomized to the long-treatment regimen (until fracture union) who had undergone 6 months or more of antibiotic treatment will be evaluated 18 months after the start of treatment (or randomization).

It will be collected:

- Clinical symptoms in relation to the patient clinical progress.

- Pain scale.

- Examination of the surgical wound: local inflammatory signs (edema, redness, cellulitis, ecchymosis), fistula (yes/no), wound drainage (amount: mild, moderate, significant; appearance: serous, hematic, purulent), dehiscence of suture (yes/no), exposure of osteosynthesis material or bone, state of the graft or skin coverage if performed.

- Radiography of the fractured bone: stability of the osteosynthesis, consolidation of the fracture.

- Completion of the SF-12 health questionnaire, or Barthel in people over 70 years of age.

- Completion of the Quick-DASH questionnaire in case of upper limb fracture or evaluation of lower limb mobility in case of upper limb bone fractures.

- Blood extraction (complete blood count and biochemistry with transaminases, bilirubin, and CRP)

- Control of dispensing and administration of medication. Reasons for ending antibiotic treatment.

- Concomitant medication review.

- Assessment of adverse events.

- Need for new surgery and type.

- New cultures if done.

<u>At this visit the primary and secondary objectives will be evaluated.</u>

9.- ASSESSMENT OF EFFICACY

9.1 Primary efficacy variable

The composite outcome variable "cure" will be used, which includes: a) clinical cure in the Test of Cure (TOC); b) radiological healing; c) definitive coverage of soft tissues in TOC. The TOC will be performed 12 months after completion of antibiotic treatment, except in patients with delayed infections randomized to the long-treatment regimen (until fracture union) who receive 6 months or more of antibiotic treatment, which will be evaluated 18 months after the start of the study (or randomization).

Clinical cure is defined as:

- The absence of clinical signs and symptoms of infection (persistence of symptoms of infection, relapse of infection after a symptom-free period, or superinfection by a different microorganism) without antibiotic therapy and with CRP <10 mg/L (unless another cause justifies a highest CRP value).
- If the patient dies, when the death was not related to the infection.
- No need for chronic suppressive antimicrobial therapy to "control" the infection.
- When the osteosynthesis material is removed once the fracture has consolidated, if the intraoperative cultures and the sonication of the osteosynthesis material are negative.

Radiological union is defined as the presence of radiological signs of fracture union (plain radiography or CT) of the infected bone. Pseudarthrosis or lack of union of a fractured bone is defined as the absence of union in the 9 months after osteosynthesis surgery or when it has not shown progression towards fracture callus formation in 3 consecutive months in serial radiographs [22].

Given the subjectivity that can sometimes occur in the evaluation of the cure criteria, a blinded investigator will assess the concordance of the clinical and radiological cure criteria performed by the local investigator.

The main variable will also be evaluated by subgroups:

- By type of infection: early and delayed.
- By type of fracture: open (Gustilo and Anderson classification) or closed.
- Type of osteosynthesis material: endomedullary nail, plates, screws...
- According to the aetiology of the infection: infections by different microorganisms, infections by multidrug resistant microorganisms.
- Depending on the type of patient: comorbidities, elderly people, kidney failure, etc.

9.2 Laboratory tests

Blood tests are contemplated at the initial visit and later as specified in the visit schedule. These analyses will be carried out locally in each center according to the standards of usual clinical practice. The following tests will be performed:

- Complete blood count including leukocyte count, neutrophil count, haemoglobin count, and platelet count.
- Blood biochemistry that includes the determination of sodium, potassium, creatinine, C-reactive protein, AST (GOT), ALT (GPT) and total bilirubin (if altered, also direct bilirubin).

9.3 Microbiological studies

Wound aspirate samples collected prior to debridement at the time of IOM diagnosis, and intraoperative samples taken at the time of debridement, will be sent to the microbiology laboratory. At least 3 intraoperative samples will be required, taken from deep tissues, bone, and peri-implant tissues. Tissue samples will be cultured for 7-10 days at 35ºC on blood agar in aerobic and anaerobic media, as well as in thioglycolate

broth. The samples may be incubated for up to 14 days in agreement with the clinician and the microbiologist in case of suspected infection by fastidious microorganisms or in patients with previous antimicrobial treatment. In the event that any orthopedic device is removed (for example, a loose screw from a plate), will be sonicated and the sonication fluid will be cultured [23].

In the event of diarrhea during antibiotic treatment, stool culture and detection of *Clostridioides difficile* toxin in stool will be performed.

10.- SECURITY ASSESSMENT

10.1 Security evaluations

The safety of all antibiotics used will be carefully followed in accordance with Regulation (EU) n. 536/2014 and Royal Decree 1090/2015.

The following clinical evaluations will be performed to assess the safety profile of the trial treatment.

10.2 Laboratory tests

Blood samples will be taken to perform a complete blood count and biochemistry (according to section 9.2) and faecal examination for *C. difficile* toxin detection (according to section 9.3).

10.3 Definitions

Adverse Event (AE):

An adverse event is any undesirable medical reaction experienced by the patient at any time during the study, whether or not considered related to the study treatment.

This definition includes the appearance of a new disease and the exacerbation of pre-existing disorders other than the indication under study.

Adverse Reaction (AR):

An AR is any harmful and unintended reaction to an investigational drug, regardless of the dose administered.

Serious Adverse Event (SAE) and Serious Adverse Reaction (SAR):

Serious AEs or ARs are those which, at any dose, may result in death, threaten the life of the subject, require hospitalization of the patient or prolong an existing hospitalization, cause permanent or significant disability or incapacity, or result in a congenital anomaly or malformation. Suspected AEs or ARs that are medically important are also considered serious, even if they do not meet the above criteria, including important medical events that require intervention to prevent the occurrence of one of the consequences described above. Likewise, all suspicions of transmission of an infectious agent through a medication will be reported as serious.

The concept “threatening the life of the subject” in the definition refers to the fact that, in the opinion of the investigator, the patient at the time of the AE or AR is at real risk of death; it does not refer to the fact that the AE/RA could hypothetically have caused death if it had been more intense.

The concept “requiring hospitalization” will exclude both planned hospitalizations for scheduled treatments and those that have been planned or are anticipated before starting the study in relation to a pre-existing medical situation.

Unexpected Adverse Reaction (RAU):

Any AR whose nature, intensity or consequences do not correspond to the reference safety information.

Serious and Unexpected Adverse Reaction (SUAR):

Any SAR (previously defined), whose nature, severity or consequences do not correspond to the reference safety information.

Causality Criteria:

- Associated AE: The temporal relationship of the AE with the study medication indicates a possible causal relationship and cannot be explained by factors such as the patient's clinical status or therapeutic interventions.
- Unrelated AE: The temporal relationship of the AE to the study medication indicates an unlikely causal relationship, or other factors (medication or concomitant conditions) or other therapeutic interventions provide a satisfactory explanation for the AE.

10.4 Reporting and collection of serious adverse events

The principal investigator or a collaborator should report to the FV-UICEC-HUVR Pharmacovigilance department all serious adverse events (as defined below), whether or not considered related or expected to the study treatment based on the following drugs: levofloxacin, moxifloxacin, rifampicin, trimethoprim-sulfamethoxazole, beta-lactams or other antibiotic used without an authorized indication for the study disease, within 24 hours (one working day), from the time they become known (Annex VII). Likewise, only serious unexpected adverse reactions (SUARs) should be reported within the same period for other drugs with an authorized indication for treatment of the study disease and extensive experience of use (see 10.3 Definitions). Serious adverse events occurring at any time from the patient's enrolment in the study (defined as the time the subject signs the informed consent) and up to 30 days after the subject completes or withdraws from the study must be reported. A subject is considered complete EITHER after the conclusion of the last visit or contact (e.g., telephone contact with the investigator or a collaborator), as indicated in the protocol evaluation schedule, OR after the last dose of study medication, whichever is later. Withdrawal is defined as the date a subject and/or the investigator determine that the subject can no longer meet the study requirements at any subsequent visits and evaluations.

The investigator will complete and sign the SAE notification form (See Annex VII) that will be sent by fax or email to:

Clinical Research and Clinical Trials Unit

Virgen del Rocío University Hospital

Dept. Pharmacovigilance

e-mail: pv_duratiom@screen.es

Avda. Manuel Siurot S/N

41013. Seville

Phone: 955 01 34 14

Fax: 955095338

FV staff will review the form received and, if appropriate, request additional information from the investigator. The investigator will provide information to the sponsor or to whoever assumes the tasks delegated by the sponsor (FV-UICEC-HUVR Unit) whenever requested and, in any case, when his initial assessment regarding severity or causality changes. To communicate the tracking information, the notification procedure described above will be followed.

The FV-UICEC-HUVR staff will keep a detailed record of all SAEs or those of special interest that are reported by the researchers.

In the event that a medication error occurs or the investigational drug is used outside the protocol provisions during the development of the study, the investigator will notify the FV-UICEC-HUVR within 24 hours of becoming aware of it. The circuit for the notification and the form will be the same as for the SAE.

Those AEs that meet the severity criteria described in the definitions section 10.1 will be considered serious. Clinically significant events that are not fatal or life-threatening or require hospitalization may be considered serious adverse drug experiences when, based on sound medical judgment, they may endanger the subject or require medical or surgical intervention to prevent one of the outcomes listed in this definition. Examples of such medical events are allergic bronchospasm requiring intensive

treatment at home or in an emergency unit, blood dyscrasias or seizures not resulting in hospitalization, or development of substance abuse or dependence.

Abnormalities in laboratory tests that meet severity criteria must also be reported unless otherwise indicated in this section of the protocol.

10.4.1 Exceptions to the collection of standard AE

Deterioration of study pathology may lead to uncertainty as to whether it is due to lack of efficacy of the trial medication, disease progression, or constitutes an AE. In these cases, unless the sponsor or the notifying physician considers that the study treatment contributed to the deterioration of the disease or local regulations establish otherwise, such deterioration will not be considered an AE but rather a loss of efficacy or disease progression if they fit the following definitions:

- Loss of Efficacy: Insufficient therapeutic effect reported as an efficacy result. Discontinuation due to insufficient therapeutic effect (ie, lack of efficacy) should not be recorded as AE. A clinical failure should not be recorded as an AE.
- Disease Progression: Disease progression can be viewed as a worsening of the subject's condition attributable to the disease for which the various trial treatments are being studied. This worsening may consist of an increase in the severity of the disease under study and/or an increase in the symptoms of the disease. If it is an expected progression, unless it is more severe in intensity or more frequent than expected for the study condition treated in the trial, it should not be recorded as an AE.

Any event or hospitalization that is prolonged due to disease progression should not be recorded as SAEs, unless the study drug is believed to have actively contributed to disease progression (insufficient therapeutic effect is not considered here).

Events that are unequivocally associated with disease progression should not be reported as AE/SAE during the active study period unless the outcome is fatal. The cause that caused the patient's death will be the one that is registered as SAE in the

CRF and notified by SAE form to FV-UICEC-HUVR within a period of 24 hours from its knowledge.

10.5 Reference security information.

Technical sheets of all the antibiotics commonly used in routine clinical practice for the treatment of IOM, with the usual doses and administration instructions described in the technical sheet or that are recommended by local and international scientific societies in each case with authorized indication.

10.6 Expedited notification of SUAR

The FV-UICEC-HUVR department is responsible for notifying the AEMPS and the Autonomous Communities where the trial is carried out, of all the SUARs that are collected in the study, following the procedure indicated in current legislation.

The FV-UICEC-HUVR Pharmacovigilance department will be in charge of making the notification through Eudravigilance_CTM.

Likewise, it must also notify the competent body of each of the Autonomous Communities where the test is carried out of suspected SUARs occurring in the health centers of your Community. For this, the SUAR notification form will be used.

The maximum period for notification of an individual case of suspected SUAR will be 15 calendar days from the moment in which the promoter became aware of it. When the suspicion of SUAR has caused the death of the patient, or put his life in danger, the promoter will send the information within a period of 7 calendar days from the moment in which he becomes aware of it. He will complete said information, if possible, in the following 8 days.

This information should include an evaluation of the importance and implication of the findings, including previous relevant experience with the same or similar drugs.

10.7 Expedited notification of other relevant safety information

The FV-UICEC-HUVR department will notify as soon as possible and no later than 15 days after it becomes aware of any information that could modify the benefit/risk ratio of the investigational drug (for example: increase in the percentage occurrence of expected SAEs, SUARs occurring after the completion of a clinical trial, new events related to the conduct of the trial or the development of the investigational medicinal product, any recommendations of the data monitoring committee relevant to the safety of the subjects, etc).

10.8 Notification to investigators

The sponsor will present to the investigators the safety information that could impact the safety of the patients included in the study as soon as possible.

In addition, the researcher will be informed throughout the study about any aspect of safety, including modifications to the protocol due to safety reasons.

10.9 Pregnancy

Subjects will be instructed to notify the investigator of pregnancy if it occurs.

In the event that any pregnancy occurs during the development of the study, the researcher will notify the sponsor or whoever assumes the tasks delegated by the sponsor within 24 hours of becoming aware of it.

Likewise, a follow-up of the pregnancy will be carried out to document its outcome and the state of health of the newborn. If the outcome of the pregnancy meets the criteria for SAE or if the newborn has a serious event, the procedures described for the notification of SAE will be followed.

The notification will be made using the specific form (see attachment VIII) which will be sent by fax or email to the same contact that will receive the SAE notifications.

11.- STATISTICS

11.1 Calculation of the sample size

According to a recent publication [14], it is estimated that the clinical cure of IOM when DAIR is performed, regardless of the duration of antibiotic treatment, is 86-100% in early IOM and 82-89% in delayed IOM. We estimate a cure rate of 85% for all early and delayed infections, both in long and short courses of antimicrobial therapy. In order to reject the null hypothesis with 80% power and a 5% one-sided significance level for a 10% noninferiority margin with a 1:1 assignment, 158 patients in each study arm (316 in total) are needed. Considering a 15% loss rate, 182 patients per arm would be required (364 in total).

11.2 Statistical analysis

The primary analysis will be the absolute comparison with one-sided 95% confidence interval of the proportion of patients reaching the composited outcome variable "cure" in the Intention-to-treat (ITT) population. Secondary analysis will include the comparison of the composite variable components (clinical cure, radiological healing and soft tissue coverage) and secondary variables in the ITT, Per protocol (PP) and Clinically evaluable (CE) populations, after excluding incorrectly assigned patients. For continuous secondary outcomes, parametric and nonparametric tests were used as appropriate.

The primary outcome will also be analyzed in the following subgroups: type of infection (early or delayed), type of fracture (open or closed), type of fractured bone, type of osteosynthesis material (endomedullary or not), type of microorganism (staphylococcus, gram-negative bacillus, other), quinolone use, age ≥70 years, renal insufficiency. Finally, a multivariate analysis will be performed in order to control the potential effect of variables other than duration of antibiotic therapy on the primary outcome by logistic regression.

11.3 Intermediate analysis

A preliminary analysis (interim analysis) will be performed when the first 75 patients have been included and monitored to ensure that there are no reasons for safety or efficacy that make it necessary to stop the trial.

11.4 Definitions of Study Analysis Populations

- Intention-to-treat (ITT) population: all randomized patients.
- Per protocol population (PP): all patients who received the complete antibiotic treatment regimen according to the inclusion group.
- Clinically evaluable population (CEP): all patients with evaluation of success in the cure test at 12 months after completion of treatment.

11.5 Independent Evaluation Committee

To avoid a possible bias due to the open nature of the trial, the evaluation of the results will be carried out by an independent committee blinded (not part of the study as investigators) with respect to the assignment of treatment. This committee will be made up of 3 expert researchers who are not participating as researchers in this study and who will reach their conclusions by consensus.

The main responsibilities of the independent committee are:

1) Periodic review and evaluation of accumulated study data for the safety of participants, progress and conduct of the study, and efficacy where appropriate, and

2) Make recommendations to the study coordination team regarding the continuation, modification or termination of the trial. For this purpose, a clear definition of the meeting calendar will be made.

The ability to recommend study termination is solely at the discretion and judgment of the Data and Safety Monitoring Board (DSMB), without any influence from the trial investigators or any other party.

The DSMB may make a binding recommendation to terminate the study if there is a substantial ethical concern as a result of any of the following:

a) Excessive AEs in the intervention arm (in terms of frequency and/or severity). Termination of the trial may be recommended if it is considered that continuation may expose participants to unacceptable risk of harm.

b) Intermediate analysis that provides statistical evidence of a positive effect; the interim analysis performed with 75 participants should show a difference in the rate of the primary variable in favour of the intervention with a type I error <0.003.

c) Intermediate analyzes that provide statistical evidence of futility. A conditional power approach computed using Mehta and Pocock's method ≤20% will be considered low enough to recommend trial termination on the basis of futility.

12.- ETHICAL ASPECTS

The trial will be carried out in accordance with the principles that emanate from the Declaration of Helsinki (see annex IX), and according to the current legal regulations (Royal Decree 1090/2015), and will not start until the approval of the reference CEIC has been obtained. the conformity of the directors of the Institutions, and the authorization of the Spanish Agency for Medicines and Health Products.

The investigator must comply with all the requirements of the protocol. In the event of a temporary deviation from the protocol, the investigator or other physician responsible for the patient should contact the monitor as soon as possible in order to discuss the situation and agree on an appropriate course of action. The investigator will document the deviation from the protocol and the circumstances that required it.

12.1 Informed consent

The patient must give their consent before being admitted to the clinical study. The doctor must explain the nature, purposes, and possible consequences of the clinical trial, in a way that is understandable to the patient. The information provided by the doctor must also be recorded. During obtaining and documenting it, the researchers will comply with the relevant legislation (article 4 of Royal Decree 1090/2015), the standards of good clinical practice and the ethical principles that have their origin in the Declaration of Helsinki.

The subject of the study will grant their consent, signing the corresponding model. The study includes minor patients, for whom the signature of the consent by their legal guardians will be required and, since they are over 12 years of age, they will also be required to sign their own consent for their participation in the study.

The investigator will not initiate any research related to the trial until the patient's consent has been obtained.

12.2 Data protection

The treatment, communication and transfer of personal data of all participating subjects will comply with the provisions of Regulation (EU) 2016/679 of the European Parliament and of the Council of April 27, 2016, regarding the protection of natural persons with regard to the processing of personal data and the free circulation of these data and Organic Law 3/2018, of December 5, on the Protection of Personal Data and the guarantee of digital rights. In accordance with the provisions of the aforementioned legislation, the patient may exercise their rights of access, modification, opposition and cancellation of data, for which they must contact their study doctor.

The anonymity of the subjects participating in the study will be maintained at all times. Thus, the data collected for the study will be identified by a code and only the researcher and collaborators will be able to relate said data to the patient and her clinical history. Therefore, the identity of the patient will not be revealed to any person except for exceptions: personnel authorized by the sponsor, when required, to check

the data and procedures of the study, but always maintaining their confidentiality in accordance with current legislation or in case of medical emergency or legal requirement (health authorities: Spanish Agency for Medicines and Health Products and Local Clinical Trials Committee).

The data from this study will be used only for the specific purposes of the study.

12.3 Responsibilities of study participants

The participating subject must follow the indications of the researchers and communicate any eventuality to them. The subject will be duly informed of the prohibitions or restrictions to which he/she must adhere during the trial.

Failure to comply with these recommendations will imply abandonment of the study.

All subjects participating in the study have the right to withdraw from the study at any time, withdrawing their consent, without having to justify this decision and without this being detrimental to their clinical follow-up. If this occurs, the investigator will attempt to have the subject perform all necessary evaluations to ensure that no adverse events occur and to ensure appropriate follow-up in the event that any problems have occurred.

12.4 Monitoring and auditing

The study will be monitored through local visits, telephone calls and periodic inspection of the CRFs frequently enough to verify the following:

- Rate of inclusion of patients, compliance with the rules of the protocol procedures, integrity and accuracy of the data entered in the notebooks, verification against the original documents and occurrence of adverse events.
- Monitoring visits will be made by study monitors. It is understood that these monitors will be able to access the medical records of the patients after requesting

it from the investigator. The investigator will dedicate sufficient time to these visits and will facilitate access to all the documentation to authorized persons.

- The study may be audited by an independent organization. Likewise, members of the CEIC will be able to monitor it.

12.5 Premature termination or suspension of the study

If the trial is terminated prematurely or suspended, the sponsor must promptly inform the investigator and regulatory authorities of the termination or suspension and the reason for it. The sponsor or investigator must promptly inform the EC and provide the reason for the termination or suspension, as specified by the relevant regulatory requirements.

12.6 Study Documentation

The documentation related to the study (protocol, CRF, informed consent, authorizations...) will be filed in a safe place and easily accessible by the research team. All information contained in clinical, histological, biochemical reports, observations or other activities is necessary for the reconstruction and evaluation of the study.

13.- FINANCING AND INSURANCE

13.1 Financing

The project has received funding through a public call for Independent Clinical Research from the Instituto de Salud Carlos III with file number ICI21/00014.

13.2 Insurance

The promoter has contracted a civil liability insurance policy in accordance with the requirements specified in article 9 of RD 1090/2015. This policy will cover all possible damages that the subject may suffer as a result of the administration of the product under study.

14.- PUBLICATION POLICY

These will comply with the provisions of Royal Decree 1090/2015 of December 4, which regulates Clinical Trials with medicines, the Ethics Committee for Research with medicines and the Spanish Registry of Clinical Studies, article 42, which pick up the following text:

"1. The promoter is obliged to publish the results, both positive and negative, of the authorized clinical trials, preferably in scientific journals before being disclosed to the non-health public, regardless of the obligations to publish the results report in the Spanish Registry of clinical studies (REec) and what is established in this regard in Regulation (EU) n. º 536/2014 of the European Parliament and of the Council, of April 16, 2014.

2. When studies and research work on medicines are made public, aimed at the scientific community, the funds obtained by the author, by or for their realization, and the source of financing shall be stated.

3. The anonymity of the subjects participating in the trial will be always maintained.

4. Undetermined efficacy of treatment will not be prematurely or sensationally disclosed, nor will it be exaggerated. Intermediate results that may compromise the reliability of the final trial results will not be published.

5. The advertising of medicines for human use in research is strictly prohibited, as established in the consolidated text of the Law on guarantees and rational use of medicines and health products, in Royal Decree 1416/1994, of June 25 , which regulates the advertising of medicines for human use, in Royal Decree 1907/1996, of

August 2, on advertising and commercial promotion of products, activities or services with intended health purposes, and in Law 34/ 1988, of November 11, General of Publicity.

6. In all cases, to make public the general results of the investigations once completed, the guidelines of the European Commission and, where appropriate, the instructions of the Spanish Agency for Medicines and Health Products will be followed.

7. When a substudy of a clinical trial end at a later date than the rest of the trial, it will be necessary for the summary of its results to be published in the year following its completion, without this implying a delay in the presentation of the results of the rest of the trial."

ANNEX I. BIBLIOGRAPHY

1. Metsemakers WJ, et al. Infection after fracture fixation of the tibia: analysis of healthcare utilization and related costs. Injury 2017;48:1204e10.
2. Olesen UK, et al. The cost of infection in severe open tibial fractures treated with a free flap. Int Orthop 2017;41:1049e55.
3. Bezstarosti H, et al. Insights into treatment and outcome of fracture-related infection: a systematic literature review. Arch Orthop Trauma Surg 2019;139:61e72.
4. Metsemakers WJ, et al. Infection after fracture fixation: Current surgical and microbiological concepts. Injury 2018;49(3):511-522.
5. Trampuz A, Zimmerli W. Diagnosis and treatment of infections associated with fracture-fixation devices. Injury 2006;37 Suppl 2:S59-66.
6. Morgenstern M, et al. Staphylococcal orthopedic device-related infections in older patients. Injury 2016;47(7):1427-34.
7. Escudero-Sanchez R, et al. Suppressive antibiotic therapy in prosthetic joint infections: a multicentre cohort study. Clin Microbiol Infect 2020;26(4):499-505.
8. Zimmerli W, et al. Role of rifampin for treatment of orthopedic implant-related staphylococcal infections: a randomized controlled trial. Foreign-Body Infection Study Group. JAMA 1998;279(19):1537-41.
9. Yen HT, et al. Short-course versus long-course antibiotics in prosthetic joint infections: a systematic review and metaanalysis of one randomized controlled trial plus nine observational studies. J Antimicrob Chemother 2019;74(9):2507-2516.
10. Benkabouche M, et al. Four versus six weeks of antibiotic therapy for osteoarticular infections after implant removal: a randomized trial. J Antimicrob Chemother 2019;74(8):2394-2399.
11. Spitzmuller R, et al. Duration of antibiotic treatment and risk of recurrence after

surgical management of orthopedic device infections: a multicenter case-control study. BMC Musculoskelet Disord 2019;20(1):184.

12. Tschudin-Sutter S, et al. Validation of a treatment algorithm for orthopedic implant-related infections with deviceretention-results from a prospective observational cohort study. Clin Microbiol Infect 2016;22(5):457.e1-9.

13. Kuehl R, et al. Time-dependent differences in management and microbiology of orthopedic internal fixation-associated infections: an observational prospective study with 229 patients. Clin Microbiol Infect 2019;25(1):76-81.

14. Morgenstern M, et al. The influence of duration of infection on outcome of debridement and implant retention in fracture-related infection. Bone Joint J. 2021;103-B(2):213-221.

15. Conterno LO, et al. Antibiotics for treating chronic osteomyelitis in adults. Cochrane Database System Rev 2013;(9): CD004439.

16. Li HK, et al.; OVIVA Trial Collaborators. Oral versus Intravenous Antibiotics for Bone and Joint Infection. N Engl J Med. 2019;380(5):425-436.

17. Ford I, Norrie J. Pragmatic Trials. N Engl J Med 2016; 375: 454-63.

18. Loudon K, et al. The PRECIS-2 tool: designing trials that are fit for purpose. BMJ 2015; 350:h2147.

19. Zwarenstein M, et al. Improving the reporting of pragmatic trials: an extension of the CONSORT statement. BMJ 2008; 337:a2390.

20. Ariza J et al; Spanish Network for the Study of Infectious Diseases and SEIMC. Executive summary of management of prosthetic joint infections. Clinical practice guidelines by SEIMC. Sick Infecc Microbiol Clin. 2017;35(3):189-195.

21. Iversen K et al. Partial Oral versus Intravenous Antibiotic Treatment of Endocarditis. N Engl J Med. 2019;380(5):415-424.

22. Bell A, Templeman D, Weinlein JC. Nonunion of the Femur and Tibia: An Update. Orthop Clin North Am. 2016;47(2):365-75.

23. Trampuz A et al. Sonication of removed hip and knee prostheses for diagnosis of infection. N Engl J Med. 2007;357(7):654-63.

24. Rockwood CA Jr, et al. Rockwood and Green's fractures in adults. Vol 1, 4th ed. Philadelphia, PA: Lippincott-Raven; nineteen ninety six.

25. Borgstrom F, et al.; International Osteoporosis Foundation. Fragility fractures in Europe: burden, management and opportunities. Arch Osteoporos. 2020;15(1):59.

ANNEX II. LIST OF PARTICIPATING CENTERS

CENTER	PRINCIPAL INVESTIGATOR
H.U. Virgen Macarena	Dr. Mª Dolores del Toro López (IP)
H.U. Virgen del Rocío	Dr. Jose Manuel Lomas Cabezas
H.U. Regional Málaga	Dr. Beatriz Sobrino Díaz
H.U. Clinic de Barcelona	Dr. Laura Morata Ruiz
H.U. Bellvitge	Dr. Óscar Murillo Rubio
H. of Santa Creu i Sant Pau	Dr. Natividad de Benito
H.U. Ramón y Cajal	Dr. Javier Cobo Reinoso
H.U. Costa del Sol	Dr. Alfonso del Arco Jiménez
H.U. Vall d'Hebron	Dr. Mª Dolores Rodríguez Pardo
H.U. 12 de Octubre	Dr. Jaime Lora-Tamayo Morillo-Velarde
H.U. Marqués de Valdecilla	Dr. Marta Fernández Sampedro
H.U. Virgen de Valme	Dr. Juan E. Corzo Delgado
H.U. Puerto Real	Dr. Alberto Romero Palacios
H. del Mar	Dr. Luisa Sorlí Redó
H.U. Virgen de la Victoria	Dr. Enrique Nuño Álvarez
H.U. Son Espases	Dr. Helem H. Vílchez
H.U. Lozano Blesa	Dr. José Ramón Paño Pardo
H. Parc Taulí	Dra. Eva Van den Eynde Otero

H. San Pedro	Dr. José Ramón Blanco Ramos
H.U. Príncipe de Asturias	Dr. José María Barbero Allende
H.U. La Paz	Dra. Alicia Rico Nieto
H.U. de Cruces	Dra. Laura Guio Carrión
H.U. El Bierzo	Dr. Alberto Bahamonde Carrasco

ANNEX III. Executive summary of management of prosthetic joint infections. Clinical practice guidelines by the Spanish Society of Infectious Diseases and Clinical Microbiology (SEIMC)

Document downloaded from http://www.elsevier.es/, day 11/05/2019. This copy is for personal use. Any transmission of this document by any media or format is strictly prohibited.

Enferm Infecc Microbiol Clin. 2017;35(3):189–195

ELSEVIER

Enfermedades Infecciosas y Microbiología Clínica

www.elsevier.es/eimc

Consensus statement

Executive summary of management of prosthetic joint infections. Clinical practice guidelines by the Spanish Society of Infectious Diseases and Clinical Microbiology (SEIMC)

Javier Ariza (Coordinator)[a], Javier Cobo (Coordinator)[b,*], Josu Baraia-Etxaburu[c], Natividad Benito[d], Guillermo Bori[e], Javier Cabo[f], Pablo Corona[g], Jaime Esteban[h], Juan Pablo Horcajada[i], Jaime Lora-Tamayo[j], Oscar Murillo[k], Julián Palomino[l], Jorge Parra[m], Carlos Pigrau[n], José Luis del Pozo[o], Melchor Riera[p], Dolores Rodríguez[n], Mar Sánchez-Somolinos[q], Alex Soriano[r], M. Dolores del Toro[s], Basilio de la Torre[t], on behalf of the Spanish Network for the Study of Infectious Diseases and the Sociedad Española de Enfermedades Infecciosas Microbiología Clínica (SEIMC)

[a] Servicio de Enfermedade Infecciosas, Hospital Universitari de Bellvitge, IDIBELL, Barcelona, Spain
[b] Servicio de Enfermedades Infecciosas, Hospital Universitario Ramón y Cajal, IRYCIS, Madrid, Spain
[c] Servicio de Enfermedades Infecciosas, Hospital Universitario de Basurto, Bilbao, Spain
[d] Unidad de Enfermedades Infecciosas, Hospital de la Santa Creu i Sant Pau, Institut d'Investigació Biomèdica Sant Pau. Universitat Autònoma de Barcelona, Barcelona, Spain
[e] Servicio de Cirugía Ortopédica y Traumatología, Hospital Clinic, Barcelona, Spain
[f] Servicio de Cirugía Ortopédica y Traumatología, Hospital Universitari de Bellvitge, Barcelona, Spain
[g] Servicio de Cirugía Ortopédica y Traumatología, Hospital Universitari Vall d'Hebron, Barcelona, Spain
[h] Departamento de Microbiología Clínica, Hospital Universitario Fundación Jiménez Díaz, Madrid, Spain
[i] Servicio de Enfermedades Infecciosas, Hospital del Mar, IMIM, Barcelona, Spain
[j] Unidad de Enfermedades Infecciosas, Hospital Universitario 12 de Octubre, Madrid, Spain
[k] Servicio de Enfermedades Infecciosas, Servicio de Hospital Universitari de Bellvitge, Barcelona, Spain
[l] Unidad Clínica de Enfermedades Infecciosas, Microbiología y Medicina Preventiva, Hospital Universitario Virgen del Rocío, Sevilla, Spain
[m] Servicio de Enfermedades Infecciosas, Hospital Clínico San Cecilio de Granada, Spain
[n] Servicio de Enfermedades Infecciosas, Hospital Universitari Vall d'Hebron, Barcelona, Spain
[o] Área de Enfermedades Infecciosas y Microbiología Clínica, Clínica Universitaria de Navarra, Spain
[p] Servicio de Medicina Interna-Infecciosas, H. Son Espases, Palma de Mallorca, Spain
[q] Servicio de Microbiología y Enfermedades Infecciosas, Hospital General Universitario Gregorio Marañón, Spain
[r] Servicio de Enfermedades Infecciosas, Hospital Clínic, Barcelona, Spain
[s] Unidad Clínica de Enfermedades Infecciosas, Microbiología y Medicina Preventiva, Hospital Universitario Virgen Macarena, Sevilla, Spain
[t] Servicio de Cirugía Ortopédica y Traumatología, Hospital Universitario Ramón y Cajal, Madrid, Spain

Keywords:
Prosthetic joint infection
Arthroplasty infection
Guidelines

A B S T R A C T

The incidence of prosthetic joint infection (PJI) is expected to increase in the coming years. PJI has serious consequences for patients, and high costs for the health system. The complexity of these infections makes it necessary to organize the vast quantity of information published in the last several years. The indications for the choice of a given surgical strategy and the corresponding antimicrobial therapy are specifically reviewed.

The authors selected clinically relevant questions and then reviewed the available literature in order to give recommendations according to a pre-determined level of scientific evidence. The more controversial aspects were debated, and the final composition was agreed at an ad hoc meeting. Before its final publication, the manuscript was made available online in order that all SEIMC members were able to read it and make comments and suggestions.

© 2016 Elsevier España, S.L.U. and Sociedad Española de Enfermedades Infecciosas y Microbiología Clínica. All rights reserved.

* Corresponding author.
E-mail address: javier.cobo@salud.madrid.org (J. Cobo).

http://dx.doi.org/10.1016/j.eimc.2016.08.012
0213-005X/© 2016 Elsevier España, S.L.U. and Sociedad Española de Enfermedades Infecciosas y Microbiología Clínica. All rights reserved.

Document downloaded from http://www.elsevier.es/, day 11/05/2019. This copy is for personal use. Any transmission of this document by any media or format is strictly prohibited.

Resumen ejecutivo de tratamiento de las infecciones de prótesis articulares. Guia clínica práctica de la Sociedad Española de Enfermedades Infecciosas y Microbiologia Clínica

Palabras clave:
Infección de prótesis articular
Infección de artroplastia
Guía clínica

R E S U M E N

Se prevé un incremento de la incidencia de infección de las prótesis articulares (IPA) en los próximos años. Las IPA plantean graves consecuencias para los pacientes y un alto coste el sistema sanitario. La complejidad de estas infecciones hace que sea necesario organizar la inmensa cantidad de información publicada en los últimos años. En estas guías se revisan específicamente las indicaciones para la elección de una estrategia quirúrgica dada y el tratamiento antimicrobiano correspondiente.

Los autores seleccionaron las preguntas clínicamente relevantes y revisaron la literatura disponible con el fin de proporcionar recomendaciones de acuerdo con un grado de evidencia científica predeterminada. Los aspectos más controvertidos fueron debatidos y la redacción final se acordó en una reunión ad hoc. Antes de su publicación, el manuscrito estuvo abierto a comentarios y sugerencias de los miembros de la Sociedad Española de Enfermedades Infecciosas y Microbiología Clínica.

© 2016 Elsevier España, S.L.U. y Sociedad Española de Enfermedades Infecciosas y Microbiología Clínica. Todos los derechos reservados.

Introduction

The incidence of prosthetic joint infection (PJI) is expected to increase in the years to come. The occurrence of a PJI dramatically raises the economic costs of an arthroplasty and it is also catastrophic for the patient. The algorithm proposed by Zimmerli a decade ago represents a notable step forward in the management of these infections, and subsequent publications have confirmed its clinical usefulness. The vast quantities of data on PJI published in recent years, along with the inherent complexity of these infections, make it necessary to organize and analyze the available information.

The initiative of the present guidelines comes from the Spanish Network for the Study of Infectious Diseases (REIPI, http://reipi.org). The guideline focuses on the management of PJI by classifying all the possible therapeutic scenarios according to clinical presentation. The indications for the choice of a given surgical strategy and a particular antimicrobial therapy are specifically reviewed.

A "choice chart" was set up for the creation of these guidelines, including five possible clinical scenarios, which raised several clinical questions of interest (Fig. 1). A review of the literature published since 1970 was performed in order to answer these questions with a predetermined degree of scientific evidence.

These guidelines are addressed to professionals of orthopedic surgery, infectious disease specialists, internal medicine physicians, microbiologists, and all other health professionals responsible for the everyday management of patients with PJI. They may also be useful for other specialists who participate less frequently in the treatment of these patients, such as geriatricians, rheumatologists, physical therapy specialists, and plastic surgeons. The whole document is available in the online version.[1]

Initial assessment of a patient with PJI

What are the goals of treatment?

The aims of the treatment of a patient with PJI are to eradicate the infection, alleviate the pain and, at the same time, restore the joint's function. This makes PJI different from other infections in which the eradication of the infection alone may be sufficient for evaluating a given therapeutic strategy. In the case of PJI, all three goals must be considered in combination, since sometimes achieving one of these targets (i.e., eradication of the infection) may interfere with another (i.e., achieving a satisfactory functional outcome). This situation increases the complexity of the management of these patients, has a deep impact on the therapeutic decisions, and makes the interpretation of the literature difficult, since there is no standardized definition of therapeutic success.

What should the care of patients with PJI involve?

- *Due to the complexity of patients with PJI, they should be attended at multidisciplinary units.* **(C-III)**

What are the medical and surgical options for patients with PJI?

The main medical and surgical strategies to be considered in a patient with PJI are:

- Attempted eradication with implant retention and antibiotics (Debridement, antibiotics and implant retention: DAIR)
- Attempted eradication with implant removal and antibiotics
  - With prosthesis replacement (in a 1-step or a 2-step exchange procedure).
  - Without prosthesis replacement (arthrodesis or resection arthroplasty).
- Implant retention and long-term suppressive antibiotics (SAT), without attempted eradication.

What are the critical aspects influencing the choice of a particular medical and surgical strategy in a given patient?

The decision regarding the most appropriate medical and surgical strategy for a given patient should consider features of the prosthesis, the patient's baseline condition, his/her previous functional performance, life expectancy, desires and expectations, and also the surgical risk involved. Tsukayama's and Zimmerli's classifications of PJI are both helpful for guiding medical and surgical decisions in a given patient. These classifications are based on similar criteria, which take into account pathogenic aspects, the time of infection, and the diagnostic circumstances.

When is attempted eradication with implant retention (DAIR) indicated? What are the results?

- The best candidates for attempting eradication treatment with implant retention are those who:

Document downloaded from http://www.elsevier.es/, day 11/05/2019. This copy is for personal use. Any transmission of this document by any media or format is strictly prohibited.

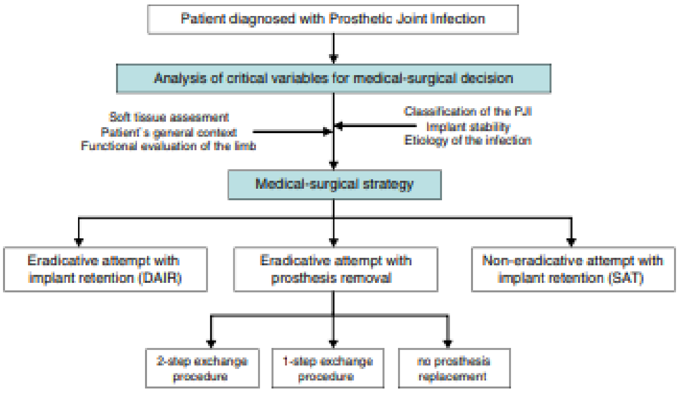

Fig. 1. Choice chart of medical-surgical stretegies for prosthetic joint infections.

  - have an early post-surgical (up to three months after the placement of the prosthesis) or hematogenous infection (**A-II**), with a stable implant, and surrounding skin and soft tissues in good condition.
  - have a short duration of symptoms (≤3 weeks) (**B-II**).
  - can be treated with rifampin (staphylococcal infections) or fluoroquinolones (infections caused by GNB) (**A-II**).
- Some patients who do not strictly meet the above criteria may still benefit from this strategy, but its implementation should be considered on an individualized basis, since there is a higher likelihood of failure (**B-II**).

In what cases of PJI should a strategy including the removal of the prosthesis be offered? What results are to be expected?

- The prosthesis should be removed in cases of chronic PJI (**A-II**).
- A 2-step exchange procedure is recommended in patients with chronic PJI (**A-II**).
- In patients with acute PJI who are not candidates for eradication treatment with implant retention, a 2-step exchange procedure is recommended (**B-II**).
- The performance of a 1-step exchange procedure may be considered in non-immunosuppressed patients if they have good bone stock, if the prosthetic surrounding soft tissues are in good condition, and if the infection is caused by microorganisms susceptible to antibiotics with good activity against sessile (biofilm-embedded) bacteria (**B-II**).
- In patients with acute PJI in whom the removal of the prosthesis is not very complex, a 1-step exchange procedure is recommended as long as the causative microorganisms are susceptible to antibiotics with good activity against biofilm-embedded bacteria (**C-III**).

In what cases of PJI should implant retention without attempted eradication be considered? What results should be expected?

Suppressive antimicrobial therapy (SAT) is seen as an alternative strategy for cases of PJI in which the surgical treatment cannot be performed or will be insufficient for eradicating the infection. The following conditions need to be met for the indication of SAT:

- Identification of the microorganism causing the infection.
- Availability of oral antibiotics which are not toxic when administered over long periods of time. Possibility of a close follow-up of the patient.

In addition, it should be considered that pain due to looseness or implant instability will be not reverted by SAT.

- Treatment with SAT may be considered in situations in which medical and surgical strategies are unlikely to cure the patient, and non-toxic long-term antimicrobials are available (**B-II**).
- Treatment with SAT is not indicated in acute PJI managed early, with appropriate debridement and optimized antimicrobial therapy (**E-II**).

Attempted eradication without implant removal

When should eradication with prosthesis retention be attempted, and what surgical technique should be used?

- Surgical debridement must be performed promptly by an expert surgical team, with the patient in the best possible condition (**C-III**).
- The surgical approach must be performed by open arthrotomy. Arthroscopy should only be considered in selected cases, and performed by expert surgeons (**A-II**).
- The surgical debridement must be aggressive, methodical and exhaustive.
  - If feasible, the removable components of the prosthesis should be exchanged (**B-II**).
  - Copious irrigation (≥9 L of saline) is recommended with no additives, performed by a low-pressure system (**C-III**).

What empirical and definitive antimicrobial treatment is recommended

See Table 1

Prior considerations regarding planktonic and sessile bacteria in the setting of PJI, and their importance in antimicrobial therapy. Foreign-body infections are characterized by the presence of sessile (biofilm-embedded) bacteria in a stationary phase of growth. However, it is also important to consider planktonic bacteria (in a logarithmic phase of growth) in these infections, especially when they are acute. An optimized initial antibiotic treatment with good activity against rapidly-growing planktonic bacteria should be provided. Once the most inflammatory component of the infection and the initial bacterial inoculum have been reduced, the treatment can focus on the biofilm-embedded bacteria.

Document downloaded from http://www.elsevier.es/, day 11/06/2019. This copy is for personal use. Any transmission of this document by any media or format is strictly prohibited.

Table 1
Empirical and targeted antimicrobial therapy in the eradicative attempt of management with implant retention.

	Recommended therapy	Alternative in patients allergic to β-lactams	Recommended duration
**Initial phase of treatment (planktonic bacteria)**			
Empirical treatment			
	Vancomycin or Daptomycin or Cloxacillin iv[a] + Ceftazidime or Cefepime or Meropenem iv	Vancomycin or Daptomycin iv + Aztreonam iv	Until the results of cultures are available
Targeted treatment			
MSSA/MSSE[*]	(Cloxacillin or Cefalozin) ± Daptomycin iv	Daptomycin + Fosfomycin iv	7–14 days
MRSA/MRSE[*]	Vancomycin (alone) or Daptomycin + (Cloxacillin or Fosfomycin) iv	Daptomycin + Fosfomycin iv	7–14 days
Streptococcus spp	Ceftriaxone or Penicillin iv	Vancomycin iv	7 days
E. faecalis	Ampicillin ± Ceftriaxone iv	Vancomycin or Teicoplanin iv	7 days
Gram-negative bacilli	β-lactam iv[**,†]	Ciprofloxacin iv	7 days
**Sequential phase treatment (biofilm-embedded bacteria)**			
Staphylococcus spp			
Treatment of choice			
	Rifampin + Levofloxacin po	–	Until completing 8 weeks
Alternatives without fluoroquinolones			
	Rifampin po + (Daptomycin or Fosfomycin) iv	–	2–4 weeks, then oral treat
	Rifampin + (LNZ, Fusidic, CMX, Clindamycin, or Minocyclin) po	–	Until completing 8 weeks of treat
Alternatives without rifampin			
	Daptomycin iv + (Fosfomycin or Cloxacillin) iv	–	2–6 weeks, then oral treat
	Daptomycin iv + (LNZ or CMX or Levofloxacin) po	–	2–6 weeks, then oral treat
	Levofloxacin + (LNZ, CMX, Clindamycin or Fusidic) po	–	Until completing 8 weeks of treat
	LNZ + (CMX or Fusidic) po	–	Until completing 8 weeks of treat
	Clindamycin + Fusidic po	–	Until completing 8 weeks of treat
	Levofloxacin or Moxifloxacin or CMX or LNZ po	–	Until completing 8 weeks of treat
Streptococcus spp			
	(Ceftriaxone or Penicillin iv) ± Rifampin po	Vancomycin iv ± Rifampin po	2–6 weeks, then oral treat
	Amoxicillin ± Rifampin po	Levofloxacin ± Rifampin po	Until completing 8 weeks of treat
	Levofloxacin ± Rifampin po	–	Until completing 8 weeks of treat
E. faecalis			
	Ampicillin ± Ceftriaxone iv	Vancomycin or Teicoplanin iv	2–6 weeks, then oral treat
	Amoxicillin ± Rifampin po	LNZ ± Rifampin po	Until completing 8 weeks of treat
E. faecium			
	Vancomycin or Teicoplanin iv		2–6 weeks, then oral treat.
	Linezolid po		Until completing 8 weeks of treat
Gram-negative bacilli			
Treatment of choice			
	Ciprofloxacin po	–	Until completing 8 weeks of treat
Alternatives without fluoroquinolones			
	β-lactam iv ± Colistin iv or β-lactam iv ± Fosfomycin iv	Aztreonam iv ± Colistin iv	6 weeks, then oral treat
	CMX	–	Until completing 8 weeks of treat
Alternatives against multi-drug resistant Gram-negative bacilli			
	β-lactam (CI) iv + Colistin iv β-lactam (CI) iv + Fosfomycin iv	Aztreonam iv (CI) + Colistin iv	6 weeks

Abbreviations: x: during; MRSA: methicillin-resistant *S. aureus*; MSSA: methicillin-susceptible *S. aureus*; MRSE: methicillin-resistant *S. epidermidis* (and other coagulase-negative staphylococci); MSSE: methicillin-susceptible *S. epidermidis* (and other coagulase-negative staphylococci). CMX: co-trimoxazole; Fusidic: fusidic acid; LNZ: linezolid; CI: continuous infusion; iv: intravenous treatment; po: *per os* (oral route); treat: treatment.

Recommended doses (assuming normal renal function): Cloxacillin, 2 g/4 h iv; Vancomycin, 1 g/12 h iv; Daptomycin, 8–10 mg/kg/24 h iv; Ceftazidime, 2 g/8 h iv; Aztreonam, 2 g/8 h iv; Cefepime, 2 g/8–12 h iv; Meropenem 1–2 g/8 h iv; Ertapenem, 1 g/24 h iv; Ceftriaxone 2 g/24 h; Ampicillin: 2 g/6 h iv; Amoxicillin, 1 g/8 h po; Rifampin, 600 mg/24 h po; Levofloxacin, 500–750 mg/24 h po; Moxifloxacin, 400 mg/24 h po; Ciprofloxacin, 400 mg/12 h iv or 750–1000 mg/12 h po; Linezolid, 600 mg/12 h po; Fusidic Acid, 500 mg/8 h po; Fosfomycin, 2 g/6 h iv; Colistin, 6–9 millions IU/d (8–12 h) iv; Co-trimoxazole 800/160 mg/8 h po; Clindamycin, 600 mg/6–8 h po; Minocycline, 200 mg/d po.

[a] The choice of a particular anti-staphylococcal agent may be conditioned by the presence of bloodstream infection, especially in hematogenous infections.

[†] The choice of a particular β-lactam agent against Gram-negative bacilli depends on the species and mechanisms of resistance: ceftriaxone is the treatment recommended for *Enterobacteriaceae*, except if they produce chromosomal β-lactamases (i.e., AMPc) or plasmidic extended-spectrum β -lactamases (ESBL); in these cases, the use of ertapenem will be preferred; in infections caused by *P. aeruginosa*, an anti-pseudomonal β-lactam is recommended.

[*] Consider adding Rifampin after the 5th day of treatment.

[**] Consider combining an anti-pseudomonal β-lactam plus ciprofloxacin in PJI caused by *P. aeruginosa*.

- After surgical debridement, antibiotics with good activity against rapidly-growing planktonic bacteria should be provided, ideally based on β-lactams, lipopeptides, or glycopeptides (**B-III**).
- This initial treatment must be administered intravenously for at least 7 days before switching to an optimized antimicrobial therapy focused on the treatment of biofilm-embedded bacteria (**C-III**).

Staphylococcal infections.

- Initial treatment (antibiotics against planktonic bacteria)
  - Methicillin-susceptible strains: cloxacillin (or cefazolin) (**B-II**), or cloxacillin + daptomycin (**C-III**).
  - Methicillin-resistant strains: daptomycin + cloxacillin, or daptomycin + fosfomycin(**C-III**), or vancomycin (**B-II**).

Document downloaded from http://www.elsevier.es/, day 11/05/2019. This copy is for personal use. Any transmission of this document by any media or format is strictly prohibited.

- Subsequent treatment (against biofilm-embedded bacteria)
  - Treatment of choice: rifampin + levofloxacin (**A-II**).
  - If fluoroquinolones cannot be used: combinations of rifampin with co-trimoxazol (**B-II**), linezolid (**B-II**), clindamycin (**B-II**), fusidic acid (**B-II**), or daptomycin (**B-III**).
  - If rifampin cannot be used: combinations of daptomycin with fosfomycin (**B-III**), cloxacillin (**B-III**), linezolid (**B-III**), co-trimoxazol (**C-III**), or levofloxacin (**C-III**); or combinations of 2 oral antibiotics or monotherapy with levofloxacin (**B-III**), or moxifloxacin (**B-III**), co-trimoxazol (**BIII**), or linezolid (**B-III**).

Streptococcal infections.

- For initial treatment (planktonic phase): penicillin or ceftriaxone (**B-II**).
- Subsequent treatment (biofilm-embedded bacteria): penicillin or ceftriaxone (**B-II**), followed by amoxicillin (**BII**), either in combination with rifampin or not (**B-III**); alternatively, levofloxacin (**B-III**) either in combination with rifampin or not (**B-III**), or monotherapy with clindamycin or linezolid in the case of allergy to fluoroquinolones (**C-III**).

Infections caused by Enterococcus faecalis.

- The treatment of choice is ampicillin, followed by oral amoxicillin (**B-II**).
- It can be administered in combination with ceftriaxone (**B-III**) or rifampin (**B-III**).
- Teicoplanin or linezolid are possible alternatives (**C-III**).

Infections caused by GNB.

- For initial treatment (planktonic phase): a β-lactam (a 3rd-generation cephalosporin for Enterobacteriaceae, a carbapenem for ESBL or AMP β-lactamase producing GNB, and an anti-pseudomonal β-lactam for *P. aeruginosa*) (**B-III**).
- Subsequent treatment (biofilm-embedded bacteria):
  - Treatment of choice: a fluoroquinolone (ciprofloxacin) (**A-II**).
  - If fluoroquinolones cannot be used (due to resistance, toxicity, etc.): continue treatment with a β-lactam (**B-III**) combined or not with colistin (**B-III**) or fosfomycin (**C-III**), or monotherapy with co-trimoxazole (**C-III**).

Culture-negative PJI.

- If possible, the use of antibiotics prior to a valid sampling (i.e. joint aspirate, and/or intraoperative cultures) should be avoided (**B-III**).
- The antimicrobial treatment must be active against the most prevalent microorganisms. The need for antibiotic activity against multi-drug resistant microorganisms must be considered in accordance with the patient's clinical and epidemiological context (**C-III**).
- If antibiotics have been administered prior to the sampling and they are considered as potentially responsible for the absence of microbiological diagnosis, the antimicrobial spectrum of this treatment should be considered when choosing the new antibiotic regime (**C-III**).

What is the optimal duration of the antimicrobial treatment?

- For acute staphylococcal PJI managed with rifampin and levofloxacin, an 8-week schedule of treatment after debridement appears sufficient for most patients (**B-I**).
- For PJI caused by other microorganisms treated with antibiotics with good activity against biofilm-embedded bacteria (i.e. ciprofloxacin for PJI caused by GNB), 8 weeks is also a reasonable duration (**B-III**).
- In other clinical scenarios, the most appropriate duration of treatment remains uncertain. A variable period between 8 and 12 weeks may be adequate (**B-III**).
- Monitoring of CRP during the follow-up is advisable; the persistence of high values is suggestive of treatment failure (**B-III**), but its total normalization must not be a condition for deciding the end of therapy (**B-II**).

How should patients be followed up and for how long?

- During antimicrobial therapy, a close follow up of observance and potential adverse events of the treatment is recommended, performed by a clinician with expertise in antibiotics (**C-III**).
- During the first 6 months after the end of a treatment aiming at eradication, patients must be followed up closely (**B-III**).
- The frequency of follow-up visits may decrease afterwards. Follow-up should last at least one year (**B-III**).

Attempted eradication with prosthesis removal and a 2-step exchange procedure

What is the role of systemic antimicrobial treatment? What is the most appropriate length and route?.

- The two-step exchange procedure should include a targeted intravenous antimicrobial treatment for 4 to 6 weeks (**A-II**), or 1–2 weeks of intravenous antibiotics followed by oral antimicrobials with good bioavailability for a total duration of 6 weeks (**B-II**).
- In chronic PJI caused by CNS, "universal" anti-staphylococcal antimicrobial therapy (i.e. glycopeptides, daptomycin, or linezolid) may be considered after the first-step surgery (prosthesis removal), because this carries a lower rate of positive cultures during the second-step surgery (re-implantation) (**C-III**).
- Shortening the systemic antimicrobial treatment could be considered for cases of PJI due to low-virulent microorganisms, such as CNS or Propionibacterium acnes, as long as the first-step surgery has included a thorough and exhaustive debridement of the joint, and a cement spacer loaded with antibiotics active against the microorganism responsible for the infection has been used (**B-II**).
- When samples taken during the second-step surgery yield a microorganism, a new 4–6 week course of antibiotics is recommended (**B-II**).

Is rifampin necessary in staphylococcal infections managed with a 2-step exchange procedure?.

- At present, it is not clear whether rifampin should be administered to treat staphylococcal infection managed with a two-step exchange procedure.
  - The indication of rifampin in a chronic non-inflammatory infection should be based on the thoroughness of the surgical debridement (**C-III**).
  - Rifampin is recommended in cases with a significant inflammatory presentation, especially those caused by *S. aureus* (**C-III**).

What is the role of local antimicrobial treatment (cement spacers)? Which kind should be used?

- Antibiotic-loaded spacers are recommended in the two-step exchange procedure (**B-II**).
- The dose of local antibiotic ranges between 0.5 and 4 g of vancomycin, and 0.25 and 4.8 g of gentamycin or tobramycin (per every 40 g of acrylic cement) (**C-III**).

Document downloaded from http://www.elsevier.es/, day 11/05/2019. This copy is for personal use. Any transmission of this document by any media or format is strictly prohibited.

- The use of combined local antibiotics (vancomycin-gentamicin) is recommended until further evidence specifically addressing this topic is available (**C-III**).
- In PJI caused by multi-drug resistant microorganisms, spacers may be still used as long as they are loaded with antibiotics active against these microorganisms (**C-III**).

When is the best time to perform the second-surgical step?

- In the two-step exchange procedure, an antibiotic-free period of 2–8 weeks and clinical stability before the second-step surgery is recommended (**C-III**).
- The monitoring of ESR and/or CRP is recommended. The persistence of values above the normal range does not necessarily indicate the persistence of the infection, and sore-implantation should not be delayed (**B-II**). However, significant changes in these serum markers may imply the persistence of the infection or a superinfection (**C-III**).

Is it necessary to take new samples for microbiological analysis before and/or during the second-step surgery? How should the results be interpreted?

- Sampling of tissues and the cement spacer during the second-step surgery of a two-step exchange procedure is recommended in order to guarantee the sterility of the surgical site where the new prosthesis is to be placed (**B-II**).
- Culture of the joint aspirate before the second-step surgery is not systematically recommended, although it may be of some use when the clinical and analytical evaluation of the patient suggests poor evolution, or in difficult-to-treat episodes caused by multi-drug resistant microorganisms or fungi (**C-II**).
- Cultures of samples taken during the second-step surgery may be considered as positive if ≥1 or ≥2 of them yield a microorganism, depending on its pathogenicity (**C-III**).

What is the best prophylaxis for the second-step surgery and how long should it be prescribed?

- Wide-spectrum antibiotic prophylaxis including nosocomial microorganisms that may potentially cause superinfection of the new prosthesis is recommended for the second-step surgery of a 2-step exchange procedure (**C-III**).
- "Preemptive treatment" including microorganisms that could be isolated during the second-step surgery (usually multi-drug resistant SNC) is recommended: vancomycin (or another glycopeptide or lipopeptide) during the first 5 days after re-implantation or until confirmation that the samples taken during the second-step surgery yield no microorganisms (**C-III**).

Attempted eradication with prosthesis removal and a 1-step exchange procedure

What is the antimicrobial treatment for patients undergoing a 1-step exchange procedure?

Recommendations

- Beginning an antimicrobial therapy 3–5 days prior to the 1-step exchange procedure is recommended if the etiological diagnosis has already been made, especially in infections caused by *S. aureus* or GNB (**C-II**).
- Regardless of the decision regarding when to start antibiotics, an appropriate antimicrobial prophylaxis throughout the procedure must be guaranteed (**A-I**).
- If no antimicrobial therapy has been initiated before the procedure, it should be delayed until the intraoperative sampling has been performed (**C-III**).

How long should antimicrobial treatment last?

- A minimum of 7 days of intravenous antibiotics with activity against the microorganisms causing the infection is recommended (dosage summarized in Table 1), followed by oral antibiotics for a total of 4–8 weeks (**B-II**).

What is the role of the local antimicrobial treatment (cement)?

- If it has been decided to use a cemented prosthesis, a local antibiotic with activity against the microorganism causing the infection is recommended. If the etiology is unknown at the moment of the exchange procedure, the combination of vancomycin plus gentamycin is recommended (**C-III**).

What is the treatment for the 'Positive intraoperative cultures' (PIOC) category of Tsukayama's classification?

- In the case of PIOC (Tsukayama's classification) an antimicrobial treatment of 4–6 weeks is recommended. There is no need for further surgery. The same protocol is followed as in cases of PJI managed with a 1-step exchange procedure (**B-III**).

What is the treatment for cases in which no new prosthesis is to be inserted after the removal of the infected one?

- For cases in which the infected prosthesis is not to be replaced after its removal, the same antibiotics as those used for DAIR may be administered (see table) (**B-II**).
- In these cases, the length of therapy may be shortened to 4 to 6 weeks (**C-III**).

  Implant retention and long-term suppressive antibiotics (SAT) without attempted eradication.

Is it necessary to perform a surgical debridement before initiating SAT?

- A surgical debridement before beginning SAT is recommended, if feasible (**C-III**).
- Obtaining a valid sample for culture before starting SAT is particularly important (**C-III**).

What are the most appropriate antibiotics for SAT? Are combinations of antimicrobials convenient or necessary? What is the role for rifampin?

- For the choice of the specific antibiotic for SAT, the antimicrobial susceptibility of the microorganism causing the infection, the safety of the drug and the observance of the treatment must be considered. Except for the initial stages of SAT, these aspects must prevail over the optimization of the antimicrobial treatment (**C-III**).
- Except for some particular cases, the use of combinations (and therefore the use of rifampin) is not recommended (**D-III**).

Is it necessary to administer intravenous antibiotics at the beginning of SAT?

- In cases undergoing surgical debridement, an initial intravenous treatment for at least 7 days is recommended. Nevertheless, prolonged intravenous treatment is not necessary when deciding on SAT management (**C-III**).

Is it possible to have defined periods with no antimicrobial treatment?

- If it is necessary to stop or change the antibiotics due to the occurrence of adverse events, long periods without antibiotics are not recommended (**D-III**).

Is SAT safe? What about its effect on the microbiota?

- The prescription and control of a SAT must be performed by an expert in antimicrobial therapy, who will periodically follow up

Document downloaded from http://www.elsevier.es/, day 11/05/2019. This copy is for personal use. Any transmission of this document by any media or format is strictly prohibited.

the clinical evolution of the infection and assess the possible occurrence of adverse events (**B-III**).

- The use of linezolid is discouraged in SAT due to high risk of toxicity, which limits its prolonged administration (**E-I**).
- The use of β-lactams, or low doses of co-trimoxazole, is recommended. Alternatively, other antimicrobials such as minocycline or clindamycin may be administered (**C-III**).

Conflict of interests

JA has served as speaker for and has received fees for advisory boards from Pfizer and Novartis.

JC has served as speaker for Astellas, AstraZeneca, MSD, Novartis and Angellini, and has received fees for advisory boards from Astellas, Pfizer, AstraZeneca and MSD.

JE has recived fees for lectures from Laboratorios Leti and support for attending conferences from Pfizer, bioMérieux, Alere, Laboratorios Leti and Novartis.

JPH has served as speaker for MSD, Astellas, Novartis, Pfizer, and Astra Zeneca and also has received fees for advisory boards from MSD, Astellas, Novartis, Pfizer, Angelini, Basilea and Astra Zeneca.

CP has received fees from Pfizer, MSD, Astellas, Novartis, Zambon, Salvat, and Mefasa-pharma.

DRP has received lecture fees, travel support for attending meetings and fees for advisory boards from Novartis, Astellas, Merck and Pfizer.

AS has received fees as speaker from Pfizer, Novartis, MSD and Astellas.

NB declares having received honoraria from Pfizer, Novartis, MSD, Astellas and Astra-Zeneca for development of educational presentations, consultancy tasks and/or for the payment of travel/accommodation for scientific purposes.

The rest of the authors declare no conflict of interests.

Acknowledgments

We thank Michael Maudsley for reviewing the English version, and Juan Manuel García-Lechuz for his critical review of the document.

J.L.-T holds a clinical research contract "Sara Borrell" (CD14/00176) from the Instituto de Salud Carlos III, Spanish Ministry of Economy and Competitiveness.

Supported by Plan Nacional de I+D+i and *Instituto de Salud Carlos III, Subdirección General de Redes y Centros de Investigación Cooperativa, Ministerio de Economía y Competitividad*, Spanish Network for Research in Infectious Diseases (REIPI RD12/0015) – co-financed by European Development Regional Fund "A way toachieve Europe" ERDF.

Appendix A. Supplementary data

Supplementary data associated with this article can be found, in the online version, at doi:10.1016/j.eimc.2016.08.012.

ANNEX IV. QUICK DASH QUIZ

*Quick*DASH

Please rate your ability to do the following activities in the last week by circling the number below the appropriate response.

	NO DIFFICULTY	MILD DIFFICULTY	MODERATE DIFFICULTY	SEVERE DIFFICULTY	UNABLE
1. Open a tight or new jar.	1	2	3	4	5
2. Do heavy household chores (e.g., wash walls, floors).	1	2	3	4	5
3. Carry a shopping bag or briefcase.	1	2	3	4	5
4. Wash your back.	1	2	3	4	5
5. Use a knife to cut food.	1	2	3	4	5
6. Recreational activities in which you take some force or impact through your arm, shoulder or hand (e.g., golf, hammering, tennis, etc.).	1	2	3	4	5

	NOT AT ALL	SLIGHTLY	MODERATELY	QUITE A BIT	EXTREMELY
7. During the past week, *to what extent* has your arm, shoulder or hand problem interfered with your normal social activities with family, friends, neighbours or groups?	1	2	3	4	5

	NOT LIMITED AT ALL	SLIGHTLY LIMITED	MODERATELY LIMITED	VERY LIMITED	UNABLE
8. During the past week, were you limited in your work or other regular daily activities as a result of your arm, shoulder or hand problem?	1	2	3	4	5

**Please rate the severity of the following symptoms in the last week.** *(circle number)*	NONE	MILD	MODERATE	SEVERE	EXTREME
9. Arm, shoulder or hand pain.	1	2	3	4	5
10. Tingling (pins and needles) in your arm, shoulder or hand.	1	2	3	4	5

	NO DIFFICULTY	MILD DIFFICULTY	MODERATE DIFFICULTY	SEVERE DIFFICULTY	SO MUCH DIFFICULTY THAT I CAN'T SLEEP
11. During the past week, how much difficulty have you had sleeping because of the pain in your arm, shoulder or hand? *(circle number)*	1	2	3	4	5

*Quick*DASH **DISABILITY/SYMPTOM SCORE** = $\left(\left[\frac{\text{(sum of n responses)}}{n}\right] - 1\right) \times 25$, where n is equal to the number of completed responses.

A *Quick*DASH score may not be calculated if there is greater than 1 missing item.

ANNEX V. SF-12 QUESTIONNAIRE AND BARTHEL INDEX

HEALTH QUESTIONNAIRE S-12

1. Would you say your health is: 1) excellent, 2) very good, 3) good, 4) fair, 5) poor

The following questions ask about activities or things that you might do on a typical day. Does your current health limit you from doing those activities or things? If so, how much?

2. Moderate efforts (such as moving a table, vacuuming, bowling, or walking for more than an hour): 1) Yes, it limits me a lot; 2) Yes, it limits me a bit; 3) No, it does not limit me
3. Going up several floors by stairs: 1) Yes, it limits me a lot; 2) Yes, it limits me a bit; 3) No, it does not limit me

During the past 4 weeks, have you had any of the following problems at work or in your daily activities due to your physical health?

4. Did you do less than you wanted to do? 1) Yes; 2) No
5. Did you have to stop doing some of the tasks at your job or in your physical activity? 1) Yes, 2) No

During the past 4 weeks, have you had any of the following problems at work or in your daily activities because of an emotional problem, such as being sad, depressed, or nervous?

6. Did you do less than you wanted to do because of some emotional problem? 1) Yes; 2) No
7. Did you have to stop doing some of your daily tasks because of an emotional problem? 1) Yes, 2) No
8. During the last 4 weeks, to what extent has pain made it difficult for you to do your usual work (including work outside the home and housework)?

   1) not at all, 2) a little, 3) fair, 4) quite a bit, 5) a lot

The questions that follow are about how you have been feeling and how things have been for you during the last 4 weeks. For each question, answer the one that is most similar to what you have felt. During the past 4 weeks, for how long…

9. …felt calm and peaceful? 1) always, 2) almost always, 3) often, 4) sometimes, 5) only sometimes, 6) never
10. …had a lot of energy? 1) always, 2) almost always, 3) often, 4) sometimes, 5) only sometimes, 6) never
11. …felt down and sad? 1) always, 2) almost always, 3) often, 4) sometimes, 5) only sometimes, 6) never
12. During the past 4 weeks, how often have physical health or emotional problems made it difficult for you to do social activities (such as visiting friends or family)? 1) always, 2) almost always, 3) sometimes, 4) only sometimes, 5) never

BARTHEL INDEX OF ACTIVITIES OF DAILY LIVING

THE Patient Name: ____________________________

BARTHEL Rater Name: ____________________________

INDEX Date: ____________________________

Activity Score

FEEDING

0 = unable

5 = needs help cutting, spreading butter, etc., or requires modified diet

10 = independent

BATHING

0 = dependent

5 = independent (or in shower)

GROOMING

0 = needs to help with personal care

5 = independent face/hair/teeth/shaving (implements provided)

DRESSING

0 = dependent

5 = needs help but can do about half unaided

10 = independent (including buttons, zips, laces, etc.)

BOWELS

0 = incontinent (or needs to be given enemas)

5 = occasional accident

10 = continent

BLADDER

0 = incontinent, or catheterized and unable to manage alone

5 = occasional accident

10 = continent

TOILET USE

0 = dependent

5 = needs some help, but can do something alone

10 = independent (on and off, dressing, wiping)

TRANSFERS (BED TO CHAIR AND BACK)

0 = unable, no sitting balance

5 = major help (one or two people, physical), can sit

10 = minor help (verbal or physical)

15 = independent

MOBILITY (ON LEVEL SURFACES)

0 = immobile or < 50 yards

5 = wheelchair independent, including corners, > 50 yards

10 = walks with help of one person (verbal or physical) > 50 yards

15 = independent (but may use any aid; for example, stick) > 50 yards

STAIRS

0 = unable

5 = needs help (verbal, physical, carrying aid)

10 = independent

TOTAL (0–100):

ANNEX VI. CALCULATION OF qSOFA AND CALCULATION OF SOFA

The Quick SOFA and SOFA will be calculated in case of qSOFA ≥ 2 through the following Links:

https://www.samiuc.es/qsofa-quick-sofa-score-para-identificacion-de-la-sepsis/

https://www.samiuc.es/sofa-score/

qSOFA (Quick SOFA) Score for Sepsis

Is the patient in the ICU?	○ Yes	○ No
Altered Mentation	○ Yes	○ No
Respiratory rate (breaths per minute)		(0 to 60)
Systolic blood pressure (mmHg)		(0 to 300)

Sequential Organ Failure Assessment Score (SOFA)

pO2 / FiO2	
Creatinine (mg/dL)	
Diuresis (L/d)	
Bilirubin (mg/dL)	
Mean arterial pressure (mm Hg)	
Platelets (thousands/mm3)	
Glasgow Coma Scale	

DA equal to or greater than 5 mcg/Kg/min or DB:	○
DA at more than 5 mcg/Kg/min or NA at equal to or less than 0.1 mcg/Kg/min:	○
DA at more than 15 mcg/Kg/min or NA at more than 0.1 mcg/Kg/min:	○

SOFA score

SOFA SCORE

Sequential Organ Failure Assessment (SOFA) Score

Parameter	Score: 0	Score: 1	Score: 2	Score: 3	Score: 4
PaO2/FIO2	< 400 mm Hg (53.3 kPa)	< 400 mm Hg (53.3 kPa)	< 300 mm Hg (40 kPa)	< 200 mm Hg (26.7 kPa) with respiratory support	< 100 mm Hg (13.3 kPa) with respiratory support
Platelets	≥ 150 × 10^3/mcL (≥ 150 × 10^9/L)	< 150 × 10^3/mcL (< 150 × 10^9/L)	< 100 × 10^3/mcL (< 100 × 10^9/L)	< 50 × 10^3/mcL (< 50 × 10^9/L)	< 20 × 10^3/mcL (< 20 × 10^9/L)
Bilirubin	< 1.2 mg/dL (20 micromole/L)	1.2–1.9 mg/dL (20–32 micromole/L)	2.0–5.9 mg/dL (33–101 micromole/L)	6.0–11.9 mg/dL (102–204 micromole/L)	> 12.0 mg/dL (204 micromole/L)
Cardiovascular	MAP ≥ 70 mm Hg	MAP < 70 mm Hg	Dopamine < 5 mcg/kg/minute for ≥ 1 hour *or* Any dose of dobutamine	Dopamine 5.1–15 mcg/kg/minute for ≥ 1 hour *or* Epinephrine ≤ 0.1 mcg/kg/minute for ≥ 1 hour *or* Norepinephrine ≤ 0.1 mcg/kg/minute for ≥ 1 hour	Dopamine > 15 mcg/kg/minute for ≥ 1 hour *or* Epinephrine > 0.1 mcg/kg/minute for ≥ 1 hour *or* Norepinephrine > 0.1 mcg/kg/minute for ≥ 1 hour
Glasgow Coma Scale score*	15 points	13–14 points	10–12 points	6–9 points	< 6 points
Creatinine	< 1.2 mg/dL (110 micromole/L)	1.2-1.9 mg/dL (110–170 micromole/L)	2.0-3.4 mg/dL (171–299 micromole/L)	3.5-4.9 mg/dL (300–400 micromole/L)	> 5.0 mg/dL (440 micromole/L)
Urine output	—	—	—	< 500 mL/day	< 200 mL/day

* A higher score indicates better neurologic function.

FIO2 = fractional inspired oxygen; kPa = kilopascals; MAP = mean arterial pressure; PaO2 = arterial oxygen partial pressure.

Adapted from Singer M, Deutschman CS, Seymour CW, et al: The third international consensus definitions for sepsis and septic shock (sepsis-3). *JAMA* 315:801–810, 2016. doi:10.1001/jama.2016.0287

Evaluation system of the appearance and evolution of Multiple Organ Failure in ICU patients. Each of the organs is scored from 0 to 4. The score is the sum of all the isolated evaluations of the organs. A score other than zero and less than 3 is evaluated as organ dysfunction, while higher scores indicate organ failure. Different works have indicated its usefulness in prognosis. An increase in the SOFA score during the first 48 hours after admission predicts a mortality greater than 49%. In SOFA > 15 points, the expected mortality is greater than 90%. It has also been shown to be useful in deciding whether or not to continue the therapeutic effort.

References:

Moreno R, Vincent JL, Matos R, et al: The use of maximum SOFA Score to quantify organ dysfunction/failure in intensive care. Results of a prospective, multicenter study. Intensive Care Medicine 1999; 25(7): 686-696

Cabré L, Mancebo JF, Solsona P, et al.: Multicenter study of the multiple organ dysfunction syndrome in intensive care units: the usefulness of Sequential Organ Failure assessment scores in decision making. Intensive Care Med 2005; 31(7): 927-933

Arts DG, de Kreizer NF, Vroom MB, de Jonge E.: Reliability and accuracy of Sequential Organ Failure Assessment (SOFA) scoring. Crit Care Med 2005; 33(9):1988-1993

Vincent JL, de Mendonca A, Cantraine F, et al.: Use of the SOFA score to assess the incidence of organ dysfunction/failure in intensive care units: Results of a multicenter, prospective study. Crit Care Med 1998; 26(11):1793-1800.

ANNEX VII. VISUAL ANALOGUE SCALE (VAS) OF PAIN

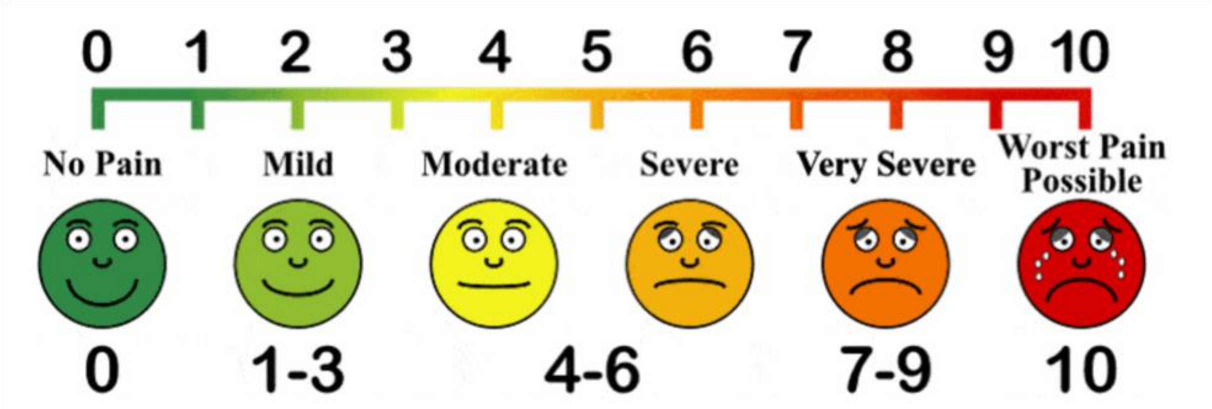

ANNEX VIII. SAE NOTIFICATION FORM

Unión Europea<br>Fondo Europeo de Desarrollo regional "Una manera de hacer Europa"<br>SCReN Spanish Clinical Research Network ISCIII	SERIOUS ADVERSE EVENT (SAE) NOTIFICATION FORM	Protocol code:
	Case number: ________<br>Notification number: ________	EudraCT No.:

Subject code: ________	REPORT TYPE: ☐ Initial ☐ Follow-up

1. CENTER INFORMATION

Center number:	Principal Investigator:	
Person notifying the SAE:		Phone:
Fax:	Mail:	

2. SUBJECT INFORMATION

Sex	Age (At the beginning of the AE)	Age group (fill in only if the age of the subject is unknown)	Date of birth (dd-mmm-yyyy)	Weig ht (kg)	Height (cm)
☐ Male<br>☐ Female	________<br>☐ Years ☐ Months ☐ Days	☐ Neonate ☐ Infant<br>☐ Child ☐ Teenayer<br>☐ Adult ☐ Old man	__-___-____		

3. ADVERSE EVENT.

Serious adverse event (Specify diagnosis or syndrome, if known. If unknown, include signs and symptoms.)**:**

....

....

DESCRIPTION OF THE ADVERSE EVENT(Provide all the information about the circumstances, the sequence, the diagnosis and the treatment of the AE)

Start date (dd-mmm-yyyy): __-___-____	End date: (dd-mmm-yyyy): __-___-____
Severity criteria:<br>☐ Death<br>☐ life threatening<br>☐ Requires or prolongs hospitalization<br>☐ Permanent or significant disability Birth<br>☐ defect or congenital anomaly Clinically<br>☐ relevant	Outcome (status at time of notification):<br>☐ Recovered<br>☐ In recoverynot<br>☐ recovered<br>☐ Recovered with deadly<br>☐ aftermath<br>☐ Unknown

In case of death, fill in the following information:

Date of death: __-___-____ Cause:________

Autopsy performed: ☐ Yes ☐ No ☐ Unknown

Indicate the cause of death according to the autopsy:________

Unión Europea<br>Fondo Europeo de Desarrollo regional<br>"Una manera de hacer Europa"<br>SCReN Spanish Clinical Research Network ISCIII	SERIOUS ADVERSE EVENT (SAE) NOTIFICATION FORM	Protocol code:
	Case number: ____________<br>Notification number: ____________	EudraCT No.:

4. INVESTIGATION PRODUCT/DRUG (Indicate the treatment(s) assigned to the subject after randomization)
If you need more space, use copies of this page and check this box ☐

Medicine	Dosage and units	Frequency	Via	Start date (dd-mmm-yyyy)	Ending date (dd-mmm-yyyy) (if you continue check the box)	Casual relationship
					☐	☐ Related<br>☐ Not related
					☐	☐ Related<br>☐ Not related
					☐	☐ Related<br>☐ Not related

Action taken with medication in response to AE	☐ Medication withdrawal<br>☐ Temporary interruption of medication<br>☐ Unknown	☐ Dose decrease<br>☐ Dose increase	☐ Without changes<br>☐ Does not apply
Did the AE resolve when the medication was stopped or the dose was reduced??	☐ Yes ☐ No	☐ Unknown	☐ No apply
Did the AE reappear when the medication was reintroduced?	☐ Yes ☐ No	☐ Unknown	☐ No apply

5. CONCOMITANT MEDICATION(Include those concomitant and background treatments that you have taken in the two weeks prior to the start date of the AE. Do not include the treatment administered to treat the AE or that administered after the start date of the AE)
If you need more space, use copies of this page and check this box ☐

Medicine	Daily dose(units)	Frequency	Via	Start date (dd-mmm-yyyy)	Ending date(dd-mmm-yyyy)(if it continues check the box)	causal relationship	Indication
					☐	☐ Related<br>☐ Not related	
					☐	☐ Related<br>☐ Not related	
					☐	☐ Related<br>☐ Not related	

Action taken with medication in response to AE	☐ Medication withdrawl<br>☐ Temporary interruption of medication<br>☐ Unknown	☐ Dose decrease<br>☐ Dose increase	☐ Without changed<br>☐ No apply
Did the AE resolve when the medication was stopped or the dose was reduced?	☐ Yes ☐ No	☐ Unknown	☐ No apply
Did the AE reappear when the medication was reintroduced?	☐ Yes ☐ No	☐ Unknown	☐ No apply

6. ALTERNATIVE CAUSE

Is there any chance that the AE is related to some other cause other than medication?
☐ Yes ☐ No If so, specify (expand information in the description section of the SAE if necessary):

____

____

Unión Europea<br>Fondo Europeo de Desarrollo regional<br>"Una manera de hacer Europa"<br>SCReN Spanish Clinical Research Network ISCIII	SERIOUS ADVERSE EVENT (SAE) NOTIFICATION FORM	Protocol code:
	Case number: ____________________<br>Notification number: ____________________	EudraCT No.:

7. RELEVANT MEDICAL HISTORY
If you need more space, use copies of this page and check this box ☐

Pathological history	Start date (dd-mmm-yyyy)	Ending date (dd-mmm-yyyy)	(If you continue check the box)
			☐
			☐
			☐
			☐

Complementary information of the Medical History data

8. LABORATORY DATA AND OTHER COMPLEMENTARY EXPLORATIONS: (list only relevant test results to document the reported SAE)
If you need more space, use copies of this page and check this box ☐

TEST CARRIED OUT	DATE TEST (dd-mmm-yyyy)	RESULT (Unitss)	Range Reference	COMMENT

Complementary information of laboratory data and other examinations:

Signature of the reporting investigator	Date of notification
Signature of the Pharmacovigilance Manager	Date of receipt of the notification

IMMEDIATELY FAX TO UICEC HUVR PHARMACOVIGILANCE NODE

pv duratiom@screnes

(FAX: 955095338)

ANNEX IX. PREGNANCY NOTIFICATION FORM

Spanish Clinical Research Network (SCReN)	PREGNANCY NOTIFICATION FORM

PROTOCOL CODE ______________________ SUBJECT Code: ___________________

EUDRA-CT No. __________________________________

REPORT TYPE:☐Initial☐Tracking Notification No.: ____________________
(Fill in only if it is a follow-up notification)

CENTER INFORMATION

Center number:		Principal investigator:	
Reporting person:			Phone:
Fax:		Mail:	

PATIENT INFORMATION

SEX	BIRTH DATE (dd-mmm-yyyy)	RACE	HEIGHT (cm)	WEIGHT (kg)	TYPE OF CONTRACEPTIVE USED
☐Men<br>☐Woman		☐White<br>☐black<br>☐Asian<br>☐Other:____			Specify:_______<br>**Was the contraceptive method used as directed?**<br>☐YES☐NO☐Unknown

INFORMATION ABOUT THE MOTHER (partner of a male patient)*(if applicable)*

BIRTH DATE (dd-mmm-yyyy)	RACE	HEIGHT(cm)	WEIGHT (kg)	TYPE OF CONTRACEPTIVE USED
	☐White<br>☐black<br>☐Asian<br>☐Other:_______			Specify:_______<br>**Was the contraceptive method used as directed?**<br>☐YES☐NO☐Unknown

PREGNANCY INFORMATION

DATE OF LAST MENSTRUATION: _____________<br>(dd-mmm-yyyy)	EXPECTED DATE OF DELIVERY:__________<br>(dd-mmm-yyyy)

RELEVANT MEDICAL HISTORY (Include family illnesses, risk factors, or conditions that may have affected the outcome of the pregnancy. If none, mark as N/A)

SOP-PFV09/01.1

Spanish Clinical Research Network SCReN	PREGNANCY NOTIFICATION FORM

PROTOCOL CODE ______________________________ SUBJECT NUMBER: ________________________

OBSTETRIC HISTORY (Provide details of all previous pregnancies, whether they were carried to term or not)

	Gestation week	Outcome including any abnormalities
1.		
2.		
3.		
4.		

TREATMENTS (include all treatments that the mother has taken from 1 month before becoming pregnant until delivery)

MEDICINE	INDICATION	PATTERN POSOLOGY	START DATE/ GESTATION WEEKS (dd-mmm-yyyy/__ __)	ENDING DATE/ GESTATION WEEKS (dd-mmm-yyyy/__ __)	INVESTIGATION MEDICATION (yes/no)

Indicate whether specific tests such as amniocentesis, ultrasound, serum alpha-fetoprotein, genetic screening, etc. have been performed during the pregnancy.

☐YES ☐NO ☐Unknown

If so, specify the test and its result:

Test: ____________________________ Date: ________________

Result: ___________________________

Test: ____________________________ Date: ________________

Result: ___________________________

Test: ____________________________ Date: ________________

Result: ___________________________

PROTOCOL CODE ______________________________ SUBJECT NUMBER: ________________________

SOP-PFV09/01.1

Unión Europea
Spanish Clinical Research Network

PREGNANCY NOTIFICATION FORM

PRENATAL INFORMATION

PREGNANCY OUTCOME

DATE OF DELIVERY, ABORTION, OR STILLBORN: ____________________ (dd-mmm-yyyy)

NUMBER OF BIRTHS: ☐SINGLE DELIVERY ☐MULTIPLE BIRTH (______)

SON	SEX	APGAR		TYPE OF DELIVERY		GESTATION WEEK	BIRTH WEIGHT	HEIGHT AT BIRTH	OUTCOME*	MALF. CONGENITAL		NEONATAL DEATH (specify cause)
		1 MIN	5 MINUTES	VAG	CAESAREAN SECTION					YES	NO	
1.							g	cm				
2.							g	cm				
3.							g	cm				
4.							g	cm				

*OUTCOME: SELECT THE APPROPRIATE NUMBER OF THE PREGNANCY OUTCOME AND WRITE IT IN THIS COLUMN:

1. BORN LIVE
2. SPONTANEOUS ABORTION (<20 WEEKS GESTATION)
3. PREMATURE FETAL DEATH (20-27 WEEKS OF GESTATION)
4. LATE FETAL DEATH (AT LEAST 28 WEEKS GESTATION)
4. INDUCED ABORTION
5. MATERNAL DEATH RESULTING IN FETAL DEATH
6. ECTOPIC PREGNANCY

ASSESSMENT OF SEVERITY (OF PREGNANCY OUTCOME)

Was severe? ☐Yes* ☐No (*If you meet seriousness criteria, fill out the corresponding SAE Form)

If yes, check all that apply:

- ☐Mother's death Date of death: ____________
- ☐Fetal death/newborn death Date of death: ____________
- ☐The patient's life has been in danger
- ☐Hospitalization/ Prolongation of hospitalization
- ☐Produces persistent or significant disability/incapacity
- ☐Congenital malformation
- ☐Other clinically relevant events

SOP-PFV09/01.1

Unión Europea<br>Spanish Clinical Research Network	**PREGNANCY NOTIFICATION FORM**

PROTOCOL CODE ______________________ SUBJECT NUMBER: ____________________	
**ASSESSMENT OF CAUSALITY (OF PREGNANCY OUTCOME)**	
Possible causes of the outcome. Check all that apply:<br>☐Underlying/concomitant disease - specify ________________<br>☐Possibly associated with trial drugs - specify ________________<br>☐Possibly associated with other concomitant/previous medications - specify ________________<br>☐Directly related to test procedures – specify ________________<br>☐Other alternative explanation - specify ________________	
**ADDITIONAL INFORMATION**	
**Signature of the reporting investigator**	**Date**
**Signature of the Pharmacovigilance Manager**	**Date**

ANNEX X. HELSINKI DECLARATION OF THE WORLD MEDICAL ASSOCIATION

Ethical principles for medical research involving human beings

Adopted by the 18th World Medical Assembly, Helsinki, Finland, June 1964 and amended by the

29th World Medical Assembly, Tokyo, Japan, October 1975

35th World Medical Assembly, Venice, Italy, October 1983

41st World Medical Assembly, Hong Kong, September 1989

48th General Assembly Somerset West, South Africa, October 1996

52nd General Assembly, Edinburgh, Scotland, October 2000

Note of Clarification of Paragraph 29, added by the General Assembly of the WMA, Washington 2002

Note of Clarification of Paragraph 30, added by the General Assembly of the WMA, Tokyo 2004

59th General Assembly, Seoul, Korea, October 2008

64th General Assembly, Fortaleza, Brazil, October 2013

A. INTRODUCTION

1. The World Medical Association (WMA) has promulgated the Declaration of Helsinki as a proposal for ethical principles for medical research involving human subjects, including the investigation of identifiable human material and information.

The Declaration must be considered as a whole, and one paragraph must be applied with consideration of all other relevant paragraphs.

2. As mandated by the WMA, the Declaration is intended primarily for physicians. The WMA encourages others involved in human medical research to adopt these principles.

B. GENERAL PRINCIPLES

3. The Declaration of Geneva of the World Medical Association links the doctor with the formula "carefully and above all for the health of my patient", and the International Code of Medical Ethics affirms that: "The doctor must consider the best for the patient when providing medical care.

4. The physician's duty is to promote and ensure the health, well-being and rights of patients, including those who participate in medical research. The knowledge and conscience of the doctor must be subordinated to the fulfillment of that duty.

5. The progress of medicine is based on research, which must ultimately include studies in humans.

6. The main purpose of medical research in human beings is to understand the causes, evolution and effects of diseases and to improve preventive, diagnostic and therapeutic interventions (methods, procedures and treatments). Even the best proven interventions need to be continually evaluated through research to be safe, efficacious, effective, accessible, and of quality.

7. Medical research is subject to ethical standards that serve to promote and ensure respect for all human beings and to protect their health and individual rights.

8. Although the main objective of medical research is to generate new knowledge, this objective should never take precedence over the rights and interests of the person participating in the research.

9. In medical research, it is the physician's duty to protect the life, health, dignity, integrity, right to self-determination, intimacy and confidentiality of the personal information of people who participate in research. The responsibility for the protection of people taking part in research must always rest with a doctor or other health professional and never with the research participants, even if they have given their consent.

10. Physicians should consider the ethical, legal, and legal norms and standards for research involving human subjects in their own countries, as well as current international norms and standards. No national or international ethical, statutory or legal requirement should be allowed to diminish or eliminate any protection measures for people participating in research established in this Declaration.

11. Medical research must be conducted in a way that minimizes potential harm to the environment.

12. Medical research involving human subjects should be conducted only by persons with appropriate scientific and ethical education, training, and qualifications. Research in healthy patients or volunteers requires the supervision of a competent and appropriately qualified physician or other healthcare professional.

13. Groups that are underrepresented in medical research should have appropriate access to participate in research.

14. Physicians who combine medical research with medical care must involve their patients in research only to the extent that this proves a justified potential preventive, diagnostic or therapeutic value and if the physician has good reason to believe that the participation in the study will not adversely affect the health of patients taking part in the research.

15. Appropriate compensation and treatment must be ensured for people who are harmed during their participation in research.

C. RISKS, COSTS AND BENEFITS

16. In the practice of medicine and medical research, most interventions involve some risk and cost.

Medical research involving human beings should only be carried out when the importance of its objective is greater than the risk and costs to the person participating in the research.

17. All medical research involving human beings must be preceded by a careful comparison of the risks and costs for the individuals and groups participating in the research, in comparison with the foreseeable benefits for them and for other individuals or groups affected by the research disease under investigation.

Measures must be implemented to minimize risks. Risks must be continuously monitored, evaluated and documented by the investigator.

18. Physicians should not engage in human research studies unless they are confident that the risks have been adequately assessed and can be satisfactorily managed.

When the risks involved outweigh the expected benefits, or if there is conclusive evidence of definitive results, physicians should consider whether to immediately continue, modify, or discontinue the study.

D. VULNERABLE PERSONS AND GROUPS

19. Some groups and individuals subject to research are particularly vulnerable and may be more likely to be abused or further harmed.

All groups and individuals must receive specific protection.

20. Medical research in a vulnerable group is only justified if the research responds to the health needs or priorities of this group and the research cannot be carried out in a non-vulnerable group. In addition, this group will be able to benefit from the knowledge, practices or interventions derived from the research.

E. SCIENTIFIC REQUIREMENTS AND RESEARCH PROTOCOLS

21. Medical research involving human beings should conform to generally accepted scientific principles and should be supported by a thorough knowledge of the scientific literature, other relevant sources of information, as well as properly conducted laboratory and animal

experiments, where appropriate. Care must also be taken for the welfare of the animals used in the experiments.

22. The design and method of any human study must be clearly described and justified in a research protocol.

The protocol must always refer to the ethical considerations that may be applicable and must indicate how the principles set forth in this Declaration have been considered. The protocol must include information on funding, sponsors, institutional affiliations, potential conflicts of interest and incentives for study persons, and information on provisions for treating or compensating persons who have been harmed as a result of their participation in the research.

In clinical trials, the protocol should also describe appropriate arrangements for post-trial stipulations.

F. RESEARCH ETHICS COMMITTEES

23. The research protocol must be submitted for consideration, comment, advice and approval to the relevant research ethics committee before the study begins. This committee must be transparent in its operation, must be independent of the investigator, the sponsor or any other type of undue influence, and must be duly qualified. The committee must consider the laws and regulations in force in the country where the research is carried out, as well as the current international norms, but they must not be allowed to diminish or eliminate any of the protections for the people who participate in the research established in this Statement.

The committee has the right to control trials in progress. The investigator has the obligation to provide control information to the committee, especially on any serious adverse incident. No amendment to the protocol should be made without the consideration and approval of the committee. After the study is finished, the investigators must submit a final report to the committee summarizing the study's results and conclusions.

G. PRIVACY AND CONFIDENTIALITY

24. All kinds of precautions must be taken to protect the privacy of the person participating in the research and the confidentiality of their personal information.

H. INFORMED CONSENT

25. The participation of persons capable of giving informed consent in medical research must be voluntary. Although it may be appropriate to consult family members or community leaders, no person capable of giving informed consent should be included in a study unless they freely agree.

26. In medical research involving human beings capable of giving informed consent, each potential individual must receive adequate information about the objectives, methods, sources of financing, possible conflicts of interest, institutional affiliations of the investigator, estimated benefits, foreseeable risks and inconveniences derived from the experiment, post-study stipulations and all other pertinent aspects of the investigation. The potential person must be informed of the right to participate or not in the research and to withdraw their consent at any time, without exposing themselves to reprisals. Special attention should be paid to the specific information needs of each potential individual, as well as the methods used to deliver the information.

After ensuring that the individual has understood the information, the physician or other appropriately qualified person should then seek, preferably in writing, the individual's voluntary and informed consent. If consent cannot be given in writing, the process for achieving it must be formally documented and witnessed.

All people involved in medical research should have the option of being informed about the general results of the study.

27. When asking for informed consent for participation in research, the doctor must take special care when the potential individual is linked to him by a dependent relationship or if he consents under pressure. In such a situation, informed consent must be requested by a suitably qualified person who has nothing to do with that relationship.

28. When the potential individual is unable to give informed consent, the physician must seek the informed consent of the legal representative. These people should not be included in research that is not likely to benefit them, unless the research is intended to promote the health of the group represented by the potential individual and this research cannot be conducted on people capable of giving informed consent and research involves only minimal risk and cost.

29. If a potential individual participating in the research considered incapable of giving informed consent is able to give his assent to participate or not in the research, the doctor must ask for it, in addition to the consent of the legal representative. The potential individual's disagreement must be respected.

30. Research on individuals who are not physically or mentally capable of giving consent, for example unconscious patients, can be carried out only if the physical/mental condition that prevents giving informed consent is a necessary characteristic of the research group. In these circumstances, the doctor must request the informed consent of the legal representative. If such a representative is not available and if the research cannot be delayed, the study may be conducted without informed consent, provided that the specific reasons for including individuals with a disease that does not allow them to give informed consent have been stipulated in the protocol of the research and the study has been approved by a research ethics committee.

31. The physician must fully inform the patient of the aspects of the care that are related to the investigation. A patient's refusal to participate in research or their decision to withdraw should never adversely affect the doctor-patient relationship.

32. For medical research using identifiable human material or data, such as research on material or data contained in biobanks or similar repositories, the physician must seek informed consent for collection, analysis, storage, and reuse. There may be exceptional situations in which it will be impossible or impracticable to obtain consent for such research. In this situation, the research can only be carried out after being considered and approved by a research ethics committee.

I. PLACEBO USE

33. The potential benefits, risks, costs, and effectiveness of any new intervention should be evaluated by comparing it with the best proven interventions, except in the following circumstances:

- When there is no proven intervention, the use of a placebo, or no intervention, is acceptable or
- When for compelling scientific and methodological reasons, the use of any intervention less effective than the best proven, the use of a placebo or no intervention is necessary to determine the efficacy and safety of an intervention.
- and patients who receive any intervention less effective than the best proven intervention, placebo, or no intervention, will not incur additional risks, serious adverse events, or irreversible harm as a result of not receiving the best proven intervention.

Extreme care must be taken to avoid abusing this option.

J. POST TRIAL STIPULATIONS

34. Prior to the clinical trial, sponsors, investigators, and host country governments should provide post-trial access to all participants who still need an intervention that has been identified as beneficial in the trial. This information should also be provided to participants during the informed consent process.

K. REGISTRATION AND PUBLICATION OF THE RESEARCH AND DISSEMINATION OF RESULTS

35. All research studies with human subjects must be registered in a publicly available database before accepting the first person.

36. Researchers, authors, sponsors, directors and editors all have ethical obligations with respect to the publication and dissemination of the results of their research. Researchers have a duty to make the results of their human research available to the public and are responsible for the completeness and accuracy of their reports. All parties must accept the ethical standards for the delivery of information. Both negative and positive results must be published

or otherwise made publicly available. The publication must cite the source of financing, institutional affiliations and conflicts of interest. Research reports that do not adhere to the principles outlined in this Statement should not be accepted for publication.

L. INTERVENTIONS NOT PROVEN IN CLINICAL PRACTICE

37. When proven interventions do not exist in the care of a patient or other known interventions have proven ineffective, the physician, after seeking expert advice, with the informed consent of the patient or an authorized legal representative, may be allowed to use interventions not proven, if, in their judgment, this gives any hope of saving life, restoring health or alleviating suffering. Such interventions should be further investigated to assess their safety and efficacy. In all cases, such new information must be recorded and, where appropriate, made available to the public.

PATIENT INFORMATION SHEET

**STUDY TITLE**	A MULTICENTER RANDOMIZED OPEN AND PRAGMATIC PHASE 3 CLINICAL STUDY TO EVALUATE THE EFFICACY AND SAFETY OF DIFFERENT DURATIONS OF ANTIMICROBIAL TREATMENT OF INFECTIONS ASSOCIATED WITH OSTEOSYNTHESIS MATERIAL AFTER LONG BONE FRACTURE.
**STUDY CODE**	DURATIOM
**EudraCT**	2021-003914-38
**PROMOTER**	Andalusian Public Foundation for the Management of Health Research in Seville (FISEVI)
**PRINCIPAL INVESTIGATOR**	
**CENTER**	

INTRODUCTION

We are writing to inform you about a research study in which you are being invited to participate. The study has been approved by the Clinical Drug Trials Committee and the Spanish Agency for Medicines and Health Products, in accordance with current legislation, Royal Decree 1090/2015, of December 4, which regulates clinical trials with medicines.

Our intention is that you receive the correct and sufficient information so that you can assess and decide whether or not you want to participate in this study. To this end, read this information sheet carefully and we will clarify any doubts you may have. In addition, you can consult with the people you consider appropriate.

VOLUNTARY PARTICIPATION

We invite you to participate in this study because you have been diagnosed with an infection related to the surgical intervention in which a material has been implanted to stabilize the fractured bone and facilitate its consolidation (formation of the fracture callus), which is called infection associated with the osteosynthesis material after a long bone fracture. When this type of infection occurs, in addition to the surgical treatment that your traumatologist considers, it is necessary to administer antibiotic treatment that will depend on the type of infection and international recommendations, and that includes different guidelines and doses of antibiotics according to what the recommendations indicate technical sheets of each of them.

In these cases and depending on when the infection appears or is diagnosed, experts recommend treating:

- if the infection is precocious or early (that is, it appears in the first 2 weeks after surgery): between 8 and 12 weeks with antibiotics directed against the microorganisms isolated in the cultures.

- if the infection is delayed (that is, it appears between the 2nd and 10th week after

surgery): antibiotics directed against the microorganisms isolated in the cultures will be administered for a time that may vary between 12 weeks or until the fracture is consolidated (the fracture callus has formed).

As you can see, this supposes a great variability in the duration of antibiotic treatments, and there are no accurate data that tell us what duration is better, if a short treatment, 8 or 12 weeks with adequate antibiotics (depending on whether it is a early or delayed infection), or a long treatment of 12 weeks or until the fracture heals (depending on whether it is an early or delayed infection). This study is designed to obtain data on the efficacy and safety of different alternatives, and for this reason, we would like you to participate in it.

You should know that your participation in this study is voluntary and that you can decide not to participate or change your decision and withdraw your consent at any time, without altering your relationship with your doctor or causing any harm to your treatment.

THE PURPOSE OF THE STUDY

This study aims to find out if we can improve the treatment of infections associated with osteosynthesis material implanted in long bone fractures, optimizing the duration of antibiotic treatment after surgical cleaning of the wound. Prolonging the duration of treatment excessively when the infection is already cured has the risk that you receive antibiotics unnecessarily, exposing you to the adverse effects that these drugs have and the risk of developing infections due to resistant bacteria. In this study we will compare the efficacy and safety of antibiotic treatments administered in a short or long regimen depending on the type of infection.

For this study to be safe, we will make sure that you are receiving an appropriate antibiotic treatment for the bacteria that causes the infection, we will carry out the necessary diagnostic tests. Likewise, you will receive a close follow-up for a maximum of 12 months after the end of the treatment, to confirm the favorable evolution.

STUDY OVERVIEW

This study will be carried out in more than 17 hospitals throughout Spain and requires the participation of 364 patients with the same diagnosis as you.

The study consists of two possible antibiotic treatment duration guidelines:

- In the first group (experimental group) antibiotic treatment will be withdrawn after 8 weeks of treatment in case you have an early infection, provided that the signs of infection have disappeared and the source of the infection is well controlled, or of 12 weeks of treatment in case of a delayed infection.
- In the other group (control group) antibiotic treatment will be withdrawn after 12 weeks of treatment in case you have an early infection, provided that the signs of infection have disappeared and the source of the infection is well controlled, or long-term treatment until the fracture is healed (fracture callus has formed) in case you have a delayed infection.

The assignment by groups will be carried out by "random assignment", in such a way that the probability of belonging to one group and another is the same. It's like tossing a coin,

the probability of heads is the same as tails, the same happens with these groups. However, both you and the doctor will always know which group you belong to.

If you decide to participate in this study, we will follow you a maximum of 12 months after completing the assigned treatment. They will carry out all the tests that are normally carried out while you are hospitalized and during the evolutionary follow-up of your infection.

The study includes approximately 8 visits in which the study doctor will carry out the necessary complementary tests and explorations within the usual follow-up of your disease,

- On the first (screening) visit; Your physician will review your eligibility for the study and provide you with the information sheet and informed consent for your consent to participate and follow the planned procedures and visits.
- On visit day 1 of the study, assignment to short or long antibiotic treatment will be made depending on the group in which it has been included.
- In the following visits, your doctor will review your clinical status and the usual monitoring of this infection, in addition to checking if there have been any side effects of the treatment you are receiving and carrying out questionnaires to assess your quality of life and the functionality of the fractured limb. These visits may be face-to-face or by telephone, depending on your needs.
- The last visit will be face-to-face (12 months after finishing the antibiotic treatment)

Procedures	Screening (up to 72 hours after knowing the IMO aetiology)	visit 1 day 0	Visit 2 Days 7 (+/-3 days)	visit 3 Day 28 (+/-7 days)	visit 4 8 weeks (+/-7 days)	visit 5 12 weeks (+/-7 days)	visit 6 6 months (+/- 2 weeks)	unscheduled visit	visit 7 12 months (+/- 1 month)	Visit 8, 12 months after completion of antibiotic treatment4
Inclusion/exclusion criteria	X									
Informed consent	X									
Medical history / anamnesis		X								
Hematology/ biochemistry		X	X	X	X	X	X	X	X	X
Dispensing and administration of medication.		X	X	X	X	X	X	X	X	X
Adverse effects			X	X	X	X	X	X	X	X

COLLECTION AND USE OF BIOLOGICAL SAMPLES

Your participation in this clinical trial entails the collection and use of biological samples (blood, and samples from the source of the infection) for research purposes, for which Law 14/2007 on biomedical research and Royal Decree 1716/2011 will be observed. , regulations that guarantee respect for the rights that assist you. By signing this document, reviewed and favorably evaluated by the Drug Research Ethics Committee that has approved this clinical trial, you agree to the use of your samples for the purposes of this study.

The samples necessary for the study are part of those normally obtained in the diagnosis, treatment and follow-up of this type of infection, and are obtained during the usual follow-up of your illness, for which we ask for your consent.

BENEFITS AND RISKS OF YOUR PARTICIPATION IN THE STUDY

If the hypothesis is true, this trial will serve to improve the antibiotic treatment of patients like you who have this type of infection, preventing them from receiving prolonged and unnecessary antibiotic treatments that could cause adverse effects. It is possible that you will not obtain any health benefit from participating in this study, however, the data obtained may be useful for future patients who are inyour situation.

All the drugs that will be used in this study are approved by the Spanish Agency for Medicines and Health Products, duly marketed, and are part of the antibiotics that are used in routine clinical practice. Due to the different clinical situations included in this study, different antibiotics or the combination of several drugs listed below will be used, depending on the type of patient: ampicillin, amoxicillin/clavulanic acid, cefazolin, cefepime, ceftazidime, ceftriaxone, cloxacillin, daptomycin, meropenem, teicoplanin, vancomycin for intravenous administration; and for sequential oral administration amoxicillin, amoxicillin/clavulanic acid, ciprofloxacin, clindamycin, cotrimoxazole, levofloxacin, linezolid, moxifloxacin, rifampin,or others according to international recommendations.

Most of these antibiotics have side effects, some of which can be life-threatening. Adverse effects that you could experience as a result of the administration of these drugs include, among others: digestive discomfort, skin rash, allergic reactions, muscle discomfort, blood and hepatobiliary disorders, kidney problems (including kidney failure), and neurological type. In any case, the risk of suffering any of these adverse effects due to your participation in this study is not greater than that which you would have if you received the usual treatment established for your disease.

In addition, all side effects or undesirable episodes that occur during the study will be controlled and monitored, so we ask that you let the study doctors know if you have any discomfort or a new finding appears.

ALTERNATIVE TREATMENTS

Being a study in which the medication used is the usual one, if you were not to participate in the trial, the medications you would receive are the same ones that are offered to you in the study.

TREATMENT OF PERSONAL DATA

As of May 25, 2018, the new legislation in the EU on personal data is fully applicable, specifically Regulation (EU) 2016/679 of the European Parliament and of the Council of April 27, 2016 on Data Protection (RGPD). Thus,It is important that you know the following information:

In addition to the rightsthat you already know (access, modification, opposition and cancellation of data) you can now also limit the processing of data that is incorrect, request a copy or transfer to a third party (portability) the data that you have provided for the study. To exercise your rights, contact the principal investigator of the study. We remind you that the data cannot be deleted, even if you stop participating in the trial to ensure the validity of the research and comply with legal duties and drug authorization requirements. Likewise, you have the right to contact the Data Protection Agency ifwill not be satisfied.

Both the Center andthe Promoter are respectively responsible for the processing of your data and undertake to comply with the data protection regulations in force. The data collected for the study will be identified by a code, so that information that can identify you is not included, and only your study doctor / collaborators will be able to relate said data to you and your medical history. Therefore, your identity will not be disclosed to any other person except the health authorities, when required or in cases of medical emergency. The Research Ethics Committees, the representatives of the Health Authority in matters of inspection and the personnel authorized by the Promoter, may only access to verify the personal data,

The Researcher and the Promoter are obliged to keep the data collected for the study for at least 25 years after its completion. Subsequently, your personal information will only be kept by the health care center and by the sponsor for other scientific research purposes if you have given your consent to do so, and if permitted by applicable law and ethical requirements.

If we transfer your encrypted data outside the EU to our group entities, service providers or scientific researchers who collaborate with us, the participant's data will be protected with safeguards such as contracts or other mechanisms by data protection authorities. data. If the participant wants to know more about it, they can contact the main researcher of the study or the Data Protection Delegate of the Ministry of Health of the Junta de Andalucía at Avenida de la Innovación, 5, 41020-Sevilla (dpd.csalud @juntadeandalucia.es).

INSURANCE

The promoter has contracted a civil liability insurance policy in accordance with the requirements specified in article 9 of RD 1090/2015. This policy will cover all possible damages that the subject may suffer as a result of the administration of the product under study.

OTHER RELEVANT INFORMATION

There is publicly available information about this study at https://reec.aemps.es/reec/public/web.html according to Spanish legislation, in addition there will also be ainternational registry www.clinicaltrials.com.

Any new information regarding the drugs used in the study and that may affect your willingness to participate in the study, that is discovered during your participation, will be communicated to you by your doctor as soon as possible.

You can withdraw from the study at any time without giving explanations, if you decide to withdraw your consent to participate in this study, no new data will be added to the database, and you may require the destruction of all previously retained identifiable samples to avoid performing new analyses.

You should also know that you may be excluded from the study if the sponsor, principal investigator, health authorities or the ethics committeethey consider it appropriate, either for safety reasons, due to any adverse event that occurs due to the medication under study or because they consider that it is not complying with the established procedures. In either case, you will receive an adequate explanation of the reason for your withdrawal from the study and will continue toreceiving necessary medical care.

By signing the attached consent form, you agree to comply with the study procedures that have been outlined to you.

When your participation ends, you will receive the best treatment available and that your doctor considers the most appropriate for your illness.

QUESTIONS

If you have any doubts or questions regarding the study or the disease, do not hesitate to tell your doctor or your team.. PYou can contact Dr/Dra. __________________________________on the phone ______________.

They will be willing to answer all your doubts and questions before, during, and after the study.

Reminder: A completed copy of this form, as well as an original of the informed consent, must be provided to the subject.

PARTICIPANT WRITTEN INFORMED CONSENT FORM

**STUDY TITLE**	A MULTICENTER RANDOMIZED OPEN AND PRAGMATIC PHASE 3 CLINICAL STUDY TO EVALUATE THE EFFICACY AND SAFETY OF DIFFERENT DURATIONS OF ANTIMICROBIAL TREATMENT OF INFECTIONS ASSOCIATED WITH OSTEOSYNTHESIS MATERIAL AFTER LONG BONE FRACTURE.
**STUDY CODE**	DURATIOM
**EudraCT**	2021-003914-38

I (name and surname of the participant),

…………………………………………………………………………………………………..

☐I have read the information sheet given to me about the study.

☐I was able to ask questions about the study.

☐I have received enough information about the study.

☐I have spoken with ……………………………………….

☐I understand that my participation is voluntary.

☐I understand that I can withdraw from the study:

- Whenever you want.
- Without having to explain.
- Without this affecting my medical care.

I will receive a signed and dated copy of this information and informed consent sheet.

I give my consent to participate in this clinical trial and I give my consent for access to the data and use of the same in the conditions detailed in this document.

Patient's signature Investigator Signature

Date: ___ /___/___ Date:___ /___/___

(Name, signature and date to be filled in by the participant)

REPRESENTATIVE/LEGAL GUARDIAN WRITTEN INFORMED CONSENT FORM

**STUDY TITLE**	A MULTICENTER RANDOMIZED OPEN AND PRAGMATIC PHASE 3 CLINICAL STUDY TO EVALUATE THE EFFICACY AND SAFETY OF DIFFERENT DURATIONS OF ANTIMICROBIAL TREATMENT OF INFECTIONS ASSOCIATED WITH OSTEOSYNTHESIS MATERIAL AFTER LONG BONE FRACTURE.
**STUDY CODE**	DURATIOM
**EudraCT**	2021-003914-38

I (name and surname of representative/legal guardian),

………………………………………………………………………………………………..

In the capacity of .... (specify relationship with the patient) of the (name and surname of the participant),

………………………………………………………………………………………………..

I declare that:

☐I have read the information sheet given to me about the study.

☐I was able to ask questions about the study.

☐I have received enough information about the study.

☐I have spoken with …………………………………….

☐I understand that your participation is voluntary.

☐I understand that you can withdraw from the study:

- Whenever you want.
- Without having to explain.
- Without this affecting my medical care.

The patient will receive a signed and dated copy of this information sheet and informed consent.

In my presence, the patient has been given all the pertinent information to his level of understanding and agrees to participate, for which I agree to participate in this clinical trial and give my consent for access to data and use of the same under the conditions detailed in this document.

For clinical studies in minors (if applicable):

We inform you that your child will be given an information sheet and informed assent adapted to their ability to understand that they must sign.

☐Parents (both)

In the event that only one of the parents authorizes, the authorizing parent must declare one of the following:

☐I hereby confirm that the other parent does not object to our child's participation in the study.

☐The signer is the sole legal guardian.

Signature of representative/legal guardian, Signature of investigator
family member or de facto related person
Date: ___ /___/___ Date: ___ /___/___
(Name, signature and date to be completed by
the legal representative, relative or
de facto linked person)

INFORMED CONSENT FORM BEFORE WITNESS

**STUDY TITLE**	A MULTICENTER RANDOMIZED OPEN AND PRAGMATIC PHASE 3 CLINICAL STUDY TO EVALUATE THE EFFICACY AND SAFETY OF DIFFERENT DURATIONS OF ANTIMICROBIAL TREATMENT OF INFECTIONS ASSOCIATED WITH OSTEOSYNTHESIS MATERIAL AFTER LONG BONE FRACTURE.
**STUDY CODE**	DURATIOM
**EudraCT**	2021-003914-38

I (name and surname of the witness),

…………………………………………………………………………………………………..

as a witness, I affirm that in my presence Mr/Ms (name and surname of the participant) ………………………………………………………………………… has been informed ……………...............

and you have read the information sheet given to you about the study so that:

☐You have been able to ask questions about the study.

☐You have received enough information about the study.

☐Have you talked to …………………………………….

☐You understand that your participation is voluntary.

☐You understand that you can withdraw from the study:

- Whenever you want.
- Without having to explain.
- Without this affecting my medical care.

The patient will receive a signed and dated copy of this information and informed consent sheet.

The patient freely gives his consent to participate in theclinical trial and gives his consent for access to the data and use of the same in the conditions detailed in this document.

Witness signature Investigator Signature

Date: ___ /___/___ Date:___ /___/___

(Name, signature and date to be filled in by the witness)

The study participant has indicated that they cannot read/write.

The Patient Information Sheet document has been read to, reviewed and discussed with by a member of the study staff, and the participant has been given the opportunity to ask questions or discuss it with others.

The witness must be an impartial person, outside the study.

Participating in a Clinical Trial

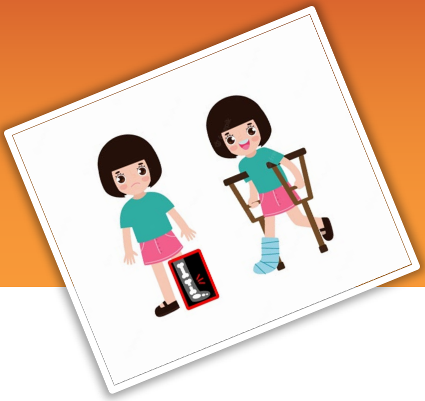

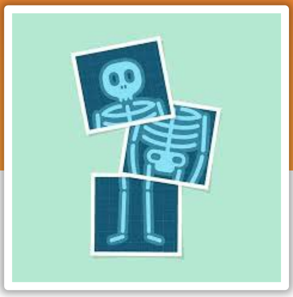

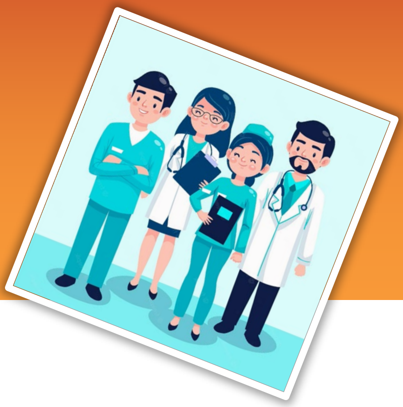

A MULTICENTER RANDOMIZED OPEN AND PRAGMATIC PHASE 3 CLINICAL STUDY TO EVALUATE THE EFFICACY AND SAFETY OF DIFFERENT DURATIONS OF ANTIMICROBIAL TREATMENT OF INFECTIONS ASSOCIATED WITH OSTEOSYNTHESIS MATERIAL AFTER LONG BONE FRACTURE

DuratIOM

Study Physician:____

Phone number:____________________

Email:____

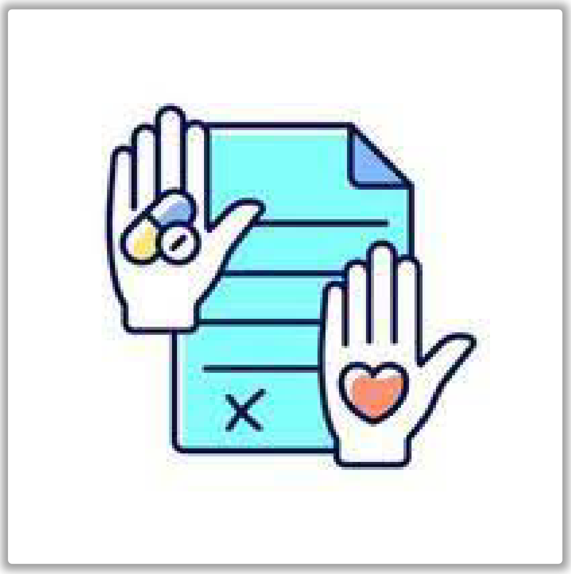

What is this document?

Your doctor has invited you to participate in a clinical trial in which you will receive a treatment for your infection. This treatment is the one we usually give you, which is the usual one, but you will be given the treatment either for as long as you usually lasts or for a shorter time. The time you get will be decided by a "raffle" among the participants.

In this document we will explain the study and what it means for you to participate in it. If after reading it you want to participate, you must sign at the end of the document. Your parents will also sign another document (informed consent) to indicate that they agree to your participation in the study.

It is important that you know that at any time you can change your mind and stop participating.

What is this study about?

After the surgery performed for the fracture of your bone, you have suffered an infection, so your doctor will prescribe some antibiotics to cure you.

In this study we want to investigate whether a shorter duration of antibiotic treatment is as effective and safe as the treatment used so far, which has a longer duration.

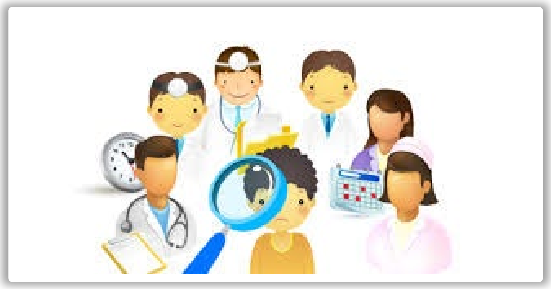

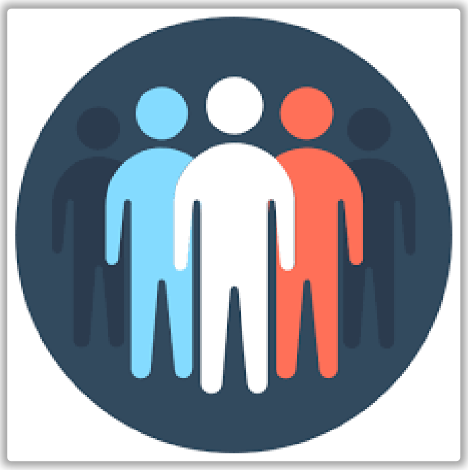

Who will this study help?

In your case, your infection will be treated equally, whether you have the short or long duration of antibiotic treatment.

In the case of other people, if this study gives the expected results, they will be able to receive antibiotic treatment for less time than the current duration of that same treatment, with the same safety and efficacy as the previous duration, but avoiding the side effects of antibiotics. prolonged treatments.

ask what you need

You can ask us what you need, for example, a word you don't understand or what you might feel during the study.

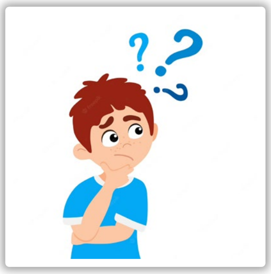

during the study

This study will involve 364 patients, including children between the ages of 14 and 18. They will be divided into 2 groups, one will receive the short treatment, for 8 weeks if the infection is early, and 12 weeks if it is delayed, and the other group will receive the long treatment, for 12 weeks if the treatment is early or until the fracture be cured in delayed infections.

Duration time

The study will last up to 12 months after finishing the antibiotic treatment and you will come to see us 8 times.

Tests to be performed

Questions related to your disease

Blood test

temperature measurement

fractured bone x-ray

end of study

The study will end 12 months after finishing the antibiotic treatment or when you and/or the doctor think you have to do it.

Adverse Events

The adverse events that could occur during the study are those expected with these antibiotic treatments, since commonly used drugs are used, and would be those indicated in the drug's package insert. In any case, we will ask you about any negative effect that you may have presented throughout the study and we will collect that information to study it.

Who will have access to my medical information?

We have informed your parents or carers about what personal data may be shared and with whom. Ask your doctor or caregivers how this information will be used.

Do I have to participate in this study?

You do not have to participate in this study if you do not want to. If you do not participate, the antibiotic treatment will be the same, but with a duration that could be longer. Your doctor will be able to inform you of all forms of treatment.

THANK YOU

We thank you for speaking with us and for considering the possibility of participating in it.

MINOR WRITTEN CONSENT FORM

Participant Signature

If you want to participate in the study, please sign, put your name and write today's date below.

You will receive a signed and dated copy of this document for you to keep.

Signature:	Date:
Name	

Signature of the person who informed the patient

I have explained the study in terms that he/she can understand and he/she agrees to participate in the study.

Signature: of the person reporting:	Date:
Reporting person's name:	
