# Supplementary material for: Efficacy and safety of different antimicrobial DURATions for the treatment of Infections associated with Osteosynthesis Material implanted after long bone fractures (DURATIOM): Protocol for a randomized, pragmatic trial
Source: PLoS One. 2023 May 22;18(5):e0286094. doi: 10.1371/journal.pone.0286094 (PMC10202272; doi:10.1371/journal.pone.0286094)
Supplement: S1 Fig — (DOCX) [file pone.0286094.s005.docx]

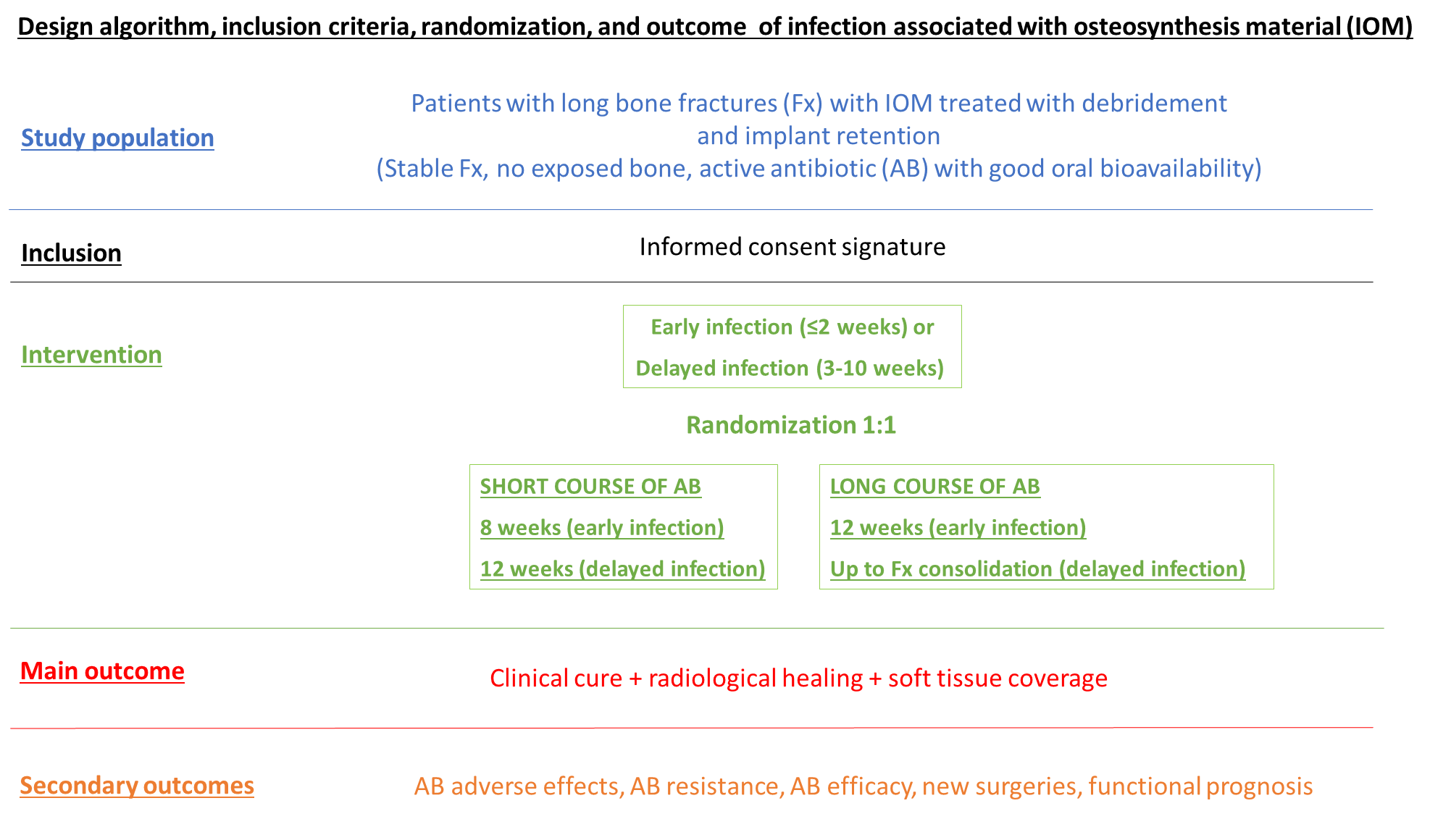
Figure S3. Design algorithm, inclusion criteria, randomization and outcome of infections associated with osteosynthesis material (IOM)

S3.Interventions and outcomes of the population of the study.
